# Discovery of Potential Neuroprotective Agents against Paclitaxel-Induced Peripheral Neuropathy

**DOI:** 10.1021/acs.jmedchem.1c01912

**Published:** 2022-03-02

**Authors:** Yi-Fan Chen, Chien-Huang Wu, Li-Hsien Chen, Hao-Wei Lee, Jinq-Chyi Lee, Teng-Kuang Yeh, Jang-Yang Chang, Ming-Chen Chou, Hui-Ling Wu, Yen-Po Lai, Jen-Shin Song, Kai-Chia Yeh, Chiung-Tong Chen, Chia-Jui Lee, Kak-Shan Shia, Meng-Ru Shen

**Affiliations:** † Institute of Biotechnology and Pharmaceutical Research, National Health Research Institutes, Miaoli County 35053, Taiwan, R. O. C.; ‡ Department of Pharmacology, College of Medicine, National Cheng Kung University, Tainan City 70101, Taiwan, R. O. C.; § Department of Obstetrics & Gynecology, National Cheng Kung University Hospital, College of Medicine, National Cheng Kung University, Tainan City 70101, Taiwan, R. O. C.; ∥ Institute of Basic Medical Sciences, College of Medicine, National Cheng Kung University, Tainan City 70101, Taiwan, R. O. C.

## Abstract

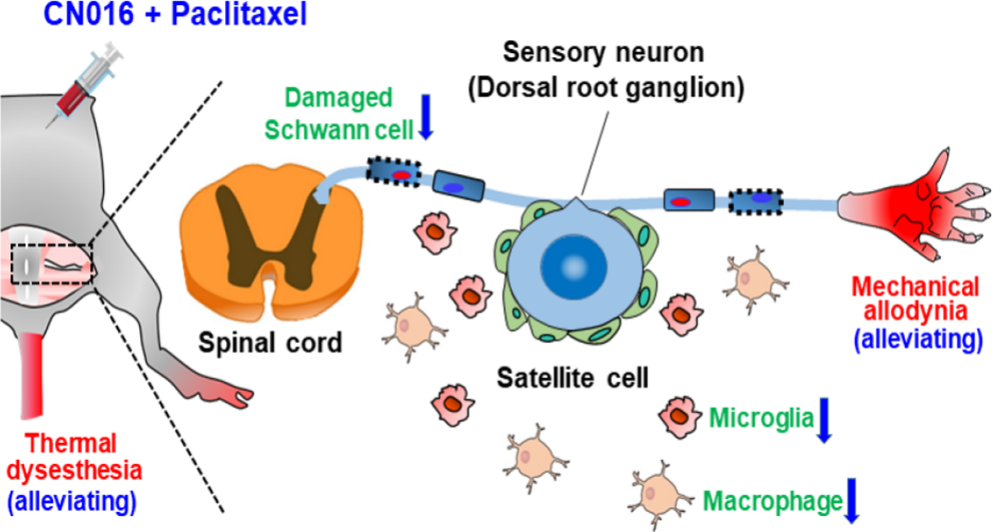

Chemotherapy-induced neurotoxicity is a common adverse effect of cancer treatment. No medication has been shown to be effective in the prevention or treatment of chemotherapy-induced neurotoxicity. Using minoxidil as an initial template for structural modifications in conjunction with an in vitro neurite outgrowth assay, an image-based high-content screening platform, and mouse behavior models, an effective neuroprotective agent CN016 was discovered. Our results showed that CN016 could inhibit paclitaxel-induced inflammatory responses and infiltration of immune cells into sensory neurons significantly. Thus, the suppression of proinflammatory factors elucidates, in part, the mechanism of action of CN016 on alleviating paclitaxel-induced peripheral neuropathy. Based on excellent efficacy in improving behavioral functions, high safety profiles (MTD > 500 mg/kg), and a large therapeutic window (MTD/MED > 50) in mice, CN016 might have great potential to become a peripherally neuroprotective agent to prevent neurotoxicity caused by chemotherapeutics as typified by paclitaxel.

## Introduction

Approximately, 19.3 million new cancer cases were diagnosed in 2020 and almost 10.0 million died from cancer worldwide. It is noteworthy that female breast cancer with an estimated 2.3 million new cases (11.7%) has overtaken lung cancer (11.4%) as the cancer occurred with the greatest frequency.^1^ Despite the sustained increases in cancer incidence, the survival rate and duration has also increased because of emerging new therapeutic modalities to treat cancer patients in recent decades.^2^ Hence, the management of side effects caused by various chemotherapeutic agents, such as neuropathy, becomes one of the important subjects for survivors after treatment.^3^

Paclitaxel is a first-line taxane-based chemotherapeutic agent treated for various malignancies such as breast, ovarian, and non-small cell lung cancers. The common side effects of paclitaxel include dizziness, neutropenia, diarrhea, and peripheral neuropathy. With the exception of neuropathy, these adverse events can be treated prophylactically or managed with agents that alleviate severity.^4^ Paclitaxel-induced peripheral neuropathy (PIPN) is in correlation with dose- and infusion-duration.^5^ Unfortunately, approximately 60–70% patients develop peripheral neuropathy after receiving paclitaxel which not only diminishes the quality of life but also frequently leads to discontinuation of treatment.^6^ Therefore, it is a critical clinical issue to develop effective neuroprotective drugs against PIPN.

PIPN mainly manifests as aberrant sensation and thermal perception abnormalities in the limbs. Patients suffering from PIPN experience numbness or tingling in hands, balance disorder, loss of tactile sensation, and thermo-sensation.^7,8^ Previous studies showed that multiple pathological mechanisms contributed to development of paclitaxel-induced neuropathy, involving metabolic dysregulation, covalent modification, organelle impairment, reactive oxygen species generation, propagation of inflammatory signals, dysfunction in axonal transport, disruption of intracellular [Ca]^2+^ homeostasis, and affected ion channel availability.^9−17^ Despite the fact that many pathways mediate neuropathy, the predominant mechanism underlying PIPN remains unclear. Because of the complexity of pathological mechanisms for development and maintenance of PIPN, there is no definite biomarker(s) to predict and evaluate the risk of PIPN, and no FDA-approved medication has been claimed to effectively treat cancer patients suffering from it.^18,19^ This unmet medical need urges us to develop novel neuroprotective agents for alleviation and/or rescue of PIPN.

Our previous studies revealed that minoxidil, a FDA-approved drug intended for the treatment of hypertension and alopecia, showed significant neuroprotective effects against paclitaxel-induced neurotoxicity both in vivo and in vitro but progressed into tachycardia and hypotension at high dosages.^20^ Besides, adverse effects such as hypertrichosis, lower-limb edema, and so forth have been regularly reported in the post-treatment assessment of oral minoxidil.^21^ This study started with using minoxidil as an initial scaffold for further structural modifications, culminating in the discovery of a novel series of neuroprotective compounds as represented by a potential drug candidate CN016, details of which are presented as follows.

## Results and Discussion

### Hit Generation by Screening Minoxidil Derivatives

**Figure 1:**
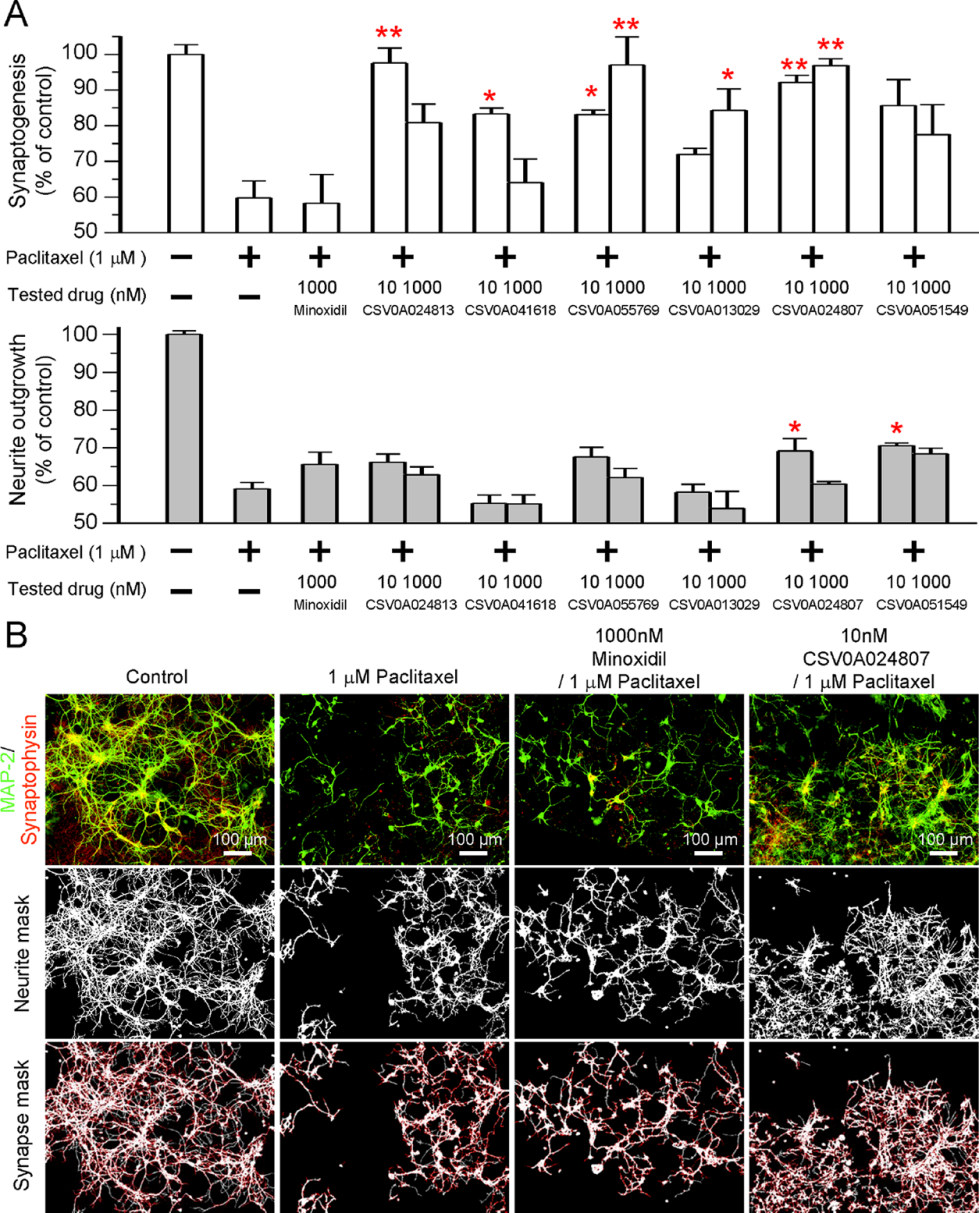
(A) Top six minoxidil-related derivatives exhibited neuroprotective effects on synaptogenesis or neurite outgrowth in primary cortical neurons against paclitaxel-induced neurotoxicity. Data represent the mean ± S.E.M. of at least three independent experiments. Statistical significance was analyzed using two-tailed Student’s *t*-test. *P < 0.05; **P < 0.01 vs paclitaxel treatment. (B) Representative images (top panels) show primary cortical neurons costained for the microtubule-associated protein 2 (MAP2, green) and synaptophysin (red) following various treatment regimens. The binary mask image (middle and bottom panels) was created from the MAP2 and the synaptophysin fluorescent signal. Scale bar, 100 μm.

**Chart 1:**
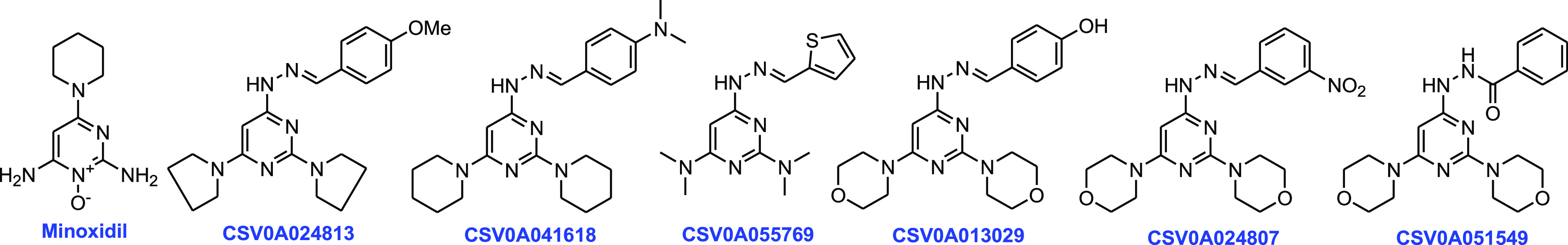
Hits Generated by Screening a Minoxidil-based Library

Herein, we wish to report that an in vitro neurite outgrowth assay of cortical neurons integrated with an image-based high-content screening (HCS) platform is used to evaluate a commercially available library of 78 minoxidil-related derivatives,^22^ leading to the identification of a series of analogues able to prevent or alleviate paclitaxel-induced neurotoxicity. The project began with screening compounds with the primary culture of cortical neurons from neonatal mice. More specifically, cerebral cortex neurons cultured from P0 C57BL/6J mouse pups were incubated with a test compound (10 and 1000 nM) prior to paclitaxel (1 μM) treatment. Subsequently, both neurite outgrowth and synaptogenesis were measured to evaluate the test compounds’ neuroprotective capacity. As illustrated in Chart 1 and Figure 1A, synaptogenesis inhibited by paclitaxel was significantly attenuated by CSV0A024813, CSV0A041618, CSV0A055769, CSV0A013029, and CSV0A024807, but only CSV0A051549 and CSV0A024807 showed a significant protective effect in the neurite outgrowth assay. However, no dose–response effects were observed for most of these compounds, presumably due to the nonspecific toxicity or a plateau effect occurring at lower concentrations. All activity data of 78 minoxidil derivatives, including inactive compounds, are provided in Table S1 for structure–activity relationship (SAR) analysis. Preliminary SAR indicates that a morpholine unit, positioned at both C2 and C6 positions, appears more effective than a five- or six-membered ring secondary amine or a dialkyl amine, implying that an extra polar interaction(s), such as hydrogen bonding, may arise from the morpholine oxygen atom. This unique feature is preserved for the core-structure screening of our in-house library in the following subject. Moreover, the substituent at the C4 position appears flexible in terms of structural diversity. Collectively, CSV0A024807, the only one displaying the best neuroprotective potency on both neurite outgrowth and synaptogenesis assays, was thus elected as a potential hit compound for further development.

Moreover, immunofluorescence images of primary cortical neuron culture and binary mask images also confirmed that neuronal damage caused by paclitaxel could be significantly rescued via pretreated CSV0A024807 (Figure 1B).

### Computer Screening of In-House Library Based on CSV0A024807 Core

Based on the core structure of CSV0A024807, structure-similarity screening of an in-house library of 150,000 compounds was then undertaken to search for a more advanced hit(s).

**Figure 2:**
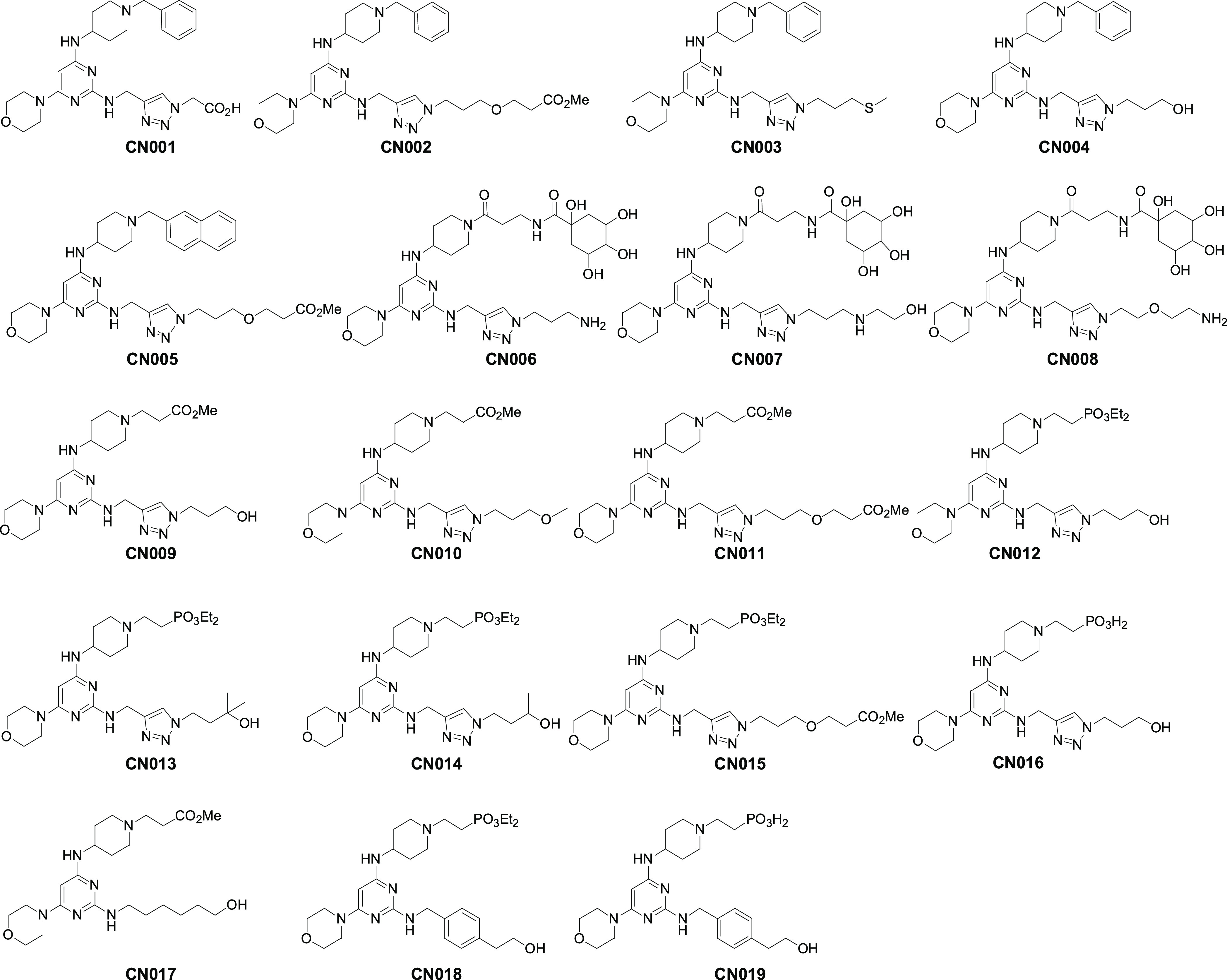
Screening an in-house library of 150,000 compounds based on hit CSV0A024807.

**Figure 3:**
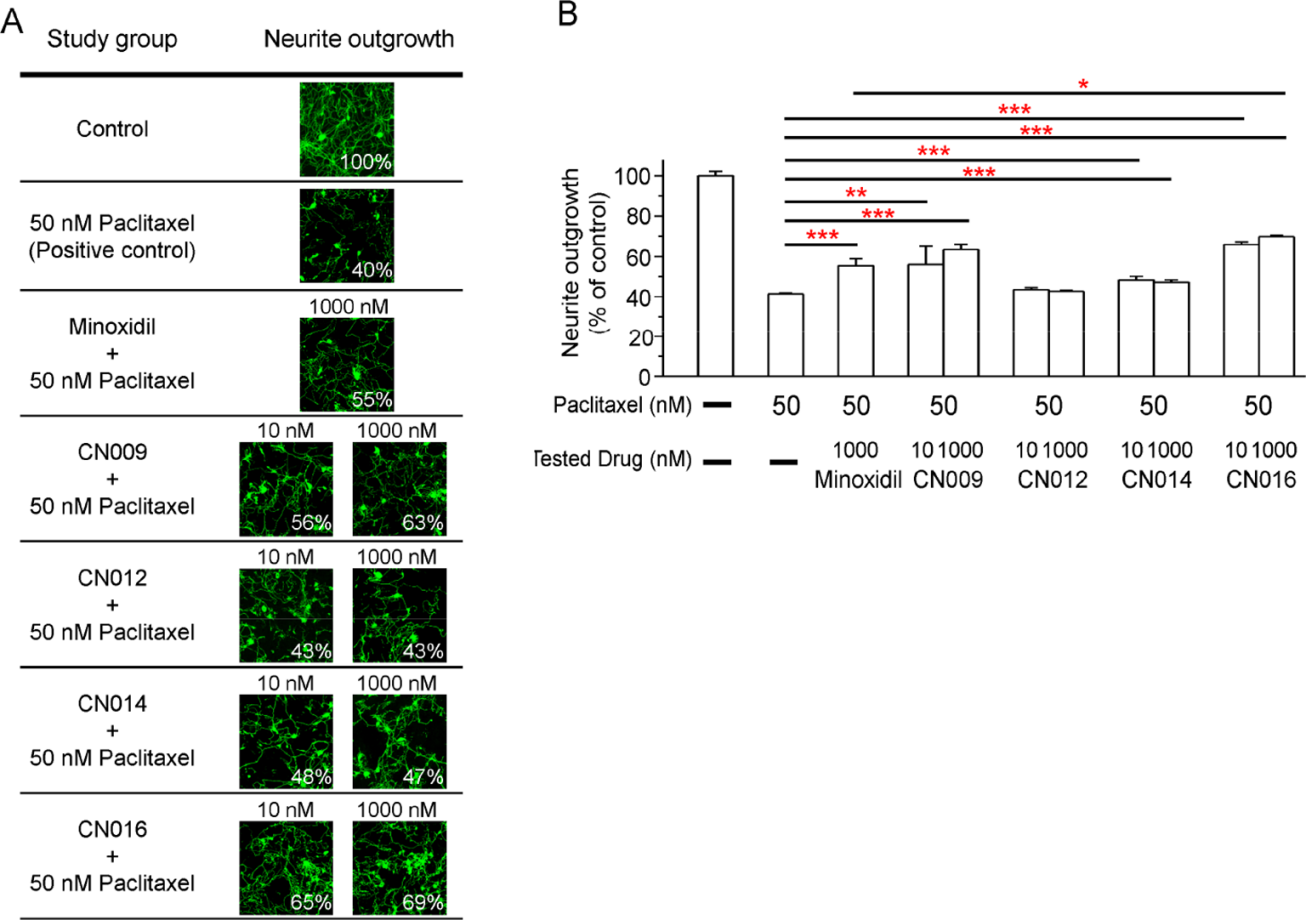
CSV0A024807-related hits tested on the neurite outgrowth after paclitaxel-induced neurotoxicity in mouse primary DRG neurons. (A) Representative images showing primary DRG neurons stained for β-III tubulin (green) following various treatment regimens. (B) Quantitative analyses of neurite outgrowth in DRG neurons. Data represent the mean ± S.E.M. from at least three different experiments. Statistical significance was analyzed using a two-tailed Student’s *t*-test. *P < 0.05. **P < 0.01. ***P < 0.001.

As a result, a class of structurally closely related pyrimidine compounds, originally designed to target G protein-coupled CXC chemokine receptor 4 (CXCR4) for disease indications such as peripheral stem-cell transplantation, hemorrhagic stroke, hepatocellular carcinoma, and so forth^23−27^ but failed to show any significant activities towards CXCR4 receptors, were unexpectedly detected (Figure 2). All these 19 compounds were then subjected to the neurite outgrowth assay of dorsal root ganglion (DRG) neurons paired with an image-based HCS to analyze whether they had neuroprotective potential. Quantification of neurite outgrowth under different regimens is shown in Figure 3, wherein DRG neurons incubated in 50 nM paclitaxel could cause 60% reduction in neurite outgrowth compared to the control (no drug treatment). Among them, only pretreated CN009, CN012, CN014, and CN016 exhibited protective efficacy against paclitaxel-induced neurotoxicity (for inactive compounds, see data in Table S2). Apparently, CN016 exhibited the most significant protection on neurons as seen in Figure 3B, wherein paclitaxel-induced damage on the neurite length was recovered by an increase of 24–28% compared to the positive control (paclitaxel-treated alone). After close examination, we found that though the diversity of this small-size library is limited, however, several useful information on SARs can be derived: (1) the C-2 linker appears to prefer a polar unit to a hydrophobic moiety as a terminal group (e.g., CN009 vs CN010); (2) the C-4 linker may prefer a phosphonate or ester terminal functionality containing multiple oxygens and disfavor an aromatic hydrocarbon unit such as a benzyl or naphthalene moiety (e.g., CN004 vs CN012); (3) the five-membered heterocyclic ring (e.g., a triazole ring) incorporated in the C-2 linker may be essential in that when it was substituted with a bisbenzyl ring or a linear linker, the corresponding compounds turned out to be inactive for neuroprotection (e.g., CN009 vs CN017, CN012 vs CN018, and CN016 vs CN019), presumably due to a different spatial orientation of the −CH_2_CH_2_OH side chain on two aromatic systems and a linear alkyl linker (see activity data in Table S2 for inactive compounds). Though CN009 and CN016 displayed almost equally neuroprotective effects, however, CN009 containing an ester group (i.e., CO_2_Me) at the terminal is considered metabolically unstable because of easy hydrolysis under catalysis with various esterases. Taken together, CN016 is selected as a promising lead for further development.

### Lead Optimization Based on CN016

**Scheme 1:**
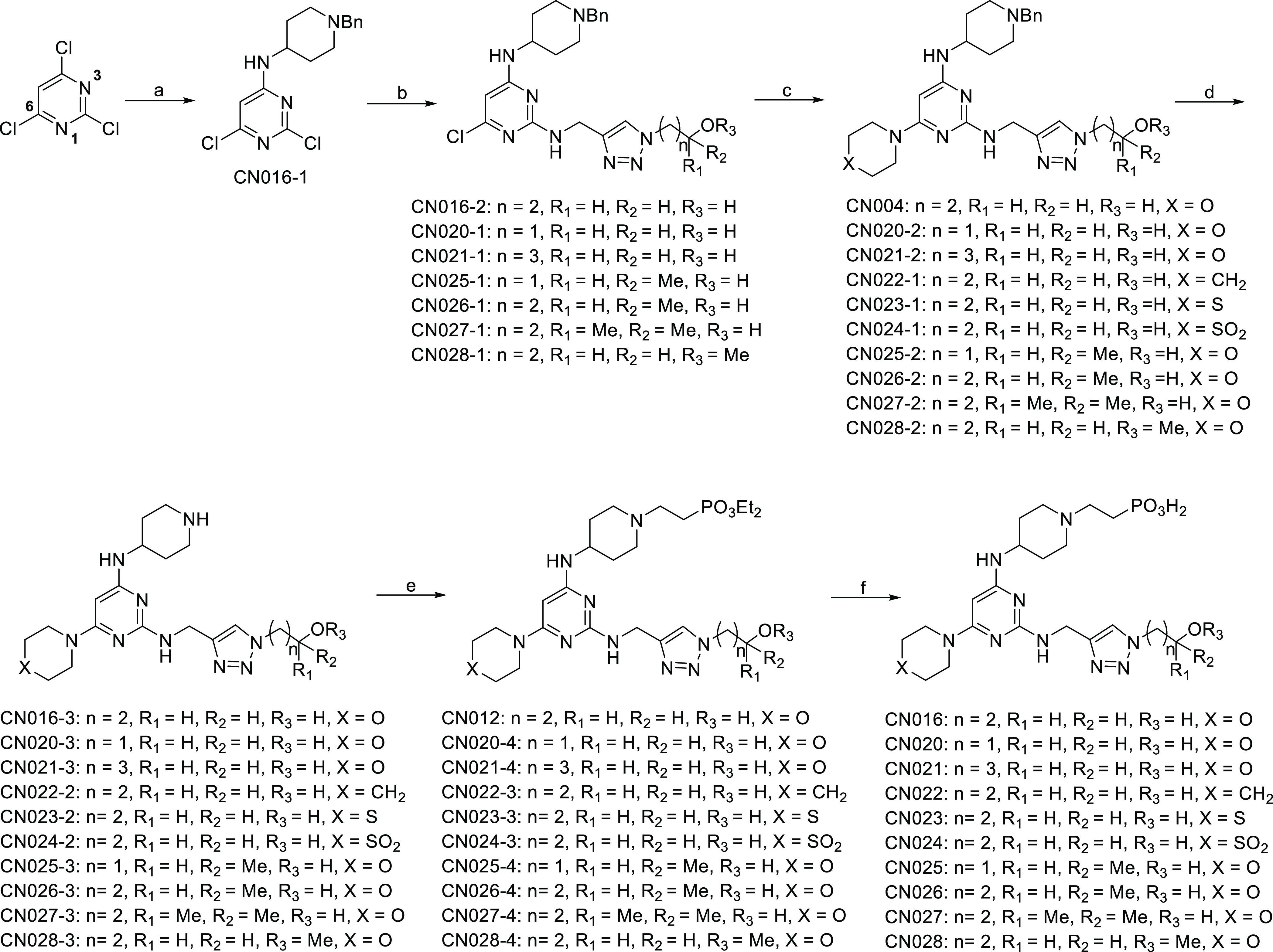
General Synthetic Procedure for Lead Optimization Compounds Reagents and conditions: (a) 4-amino-1-benzyl-piperidine, TEA, THF, 5 °C to rt, 16 h, 56%; (b) triazole linker (synthetic procedures described in Supporting Information), 1-pentanol, 140 °C, 5 h, 54–68%; (c) amine, 1-pentanol, 120 °C, 5 h, 65–89%; (d) 1 atm H_2_, Pd/C (10%), 2-propanol, 60 °C, 16 h, 88–93%; (e) diethyl vinylphosphonate, TEA, MeOH, 60 °C, 16 h, 61–70%; (f) TMSBr, CH_2_Cl_2_, 25 °C, 4 h, 82–90%.

Based on the skeleton of CN016, lead optimization was focused on modifying R_1_ and R_2_ functionalities with the retention of R_3_ containing a unique phosphonate unit. A general synthetic strategy (Scheme 1) was designed to prepare an array of analogues listed in Table 1. Starting from 2,4,6-trichloropyrimidine, C4-substitution with 4-amino-1-benzyl-piperidine was performed at room temperature to produce CN016-1 in 56% yield, which in turn underwent C2-substitution with various triazole linkers, of which synthetic routes and experimental details are reported in the Supporting Information, to afford CN016-2 and CN020-1–CN028-1 in 54–68% yields.

**Table 1:**
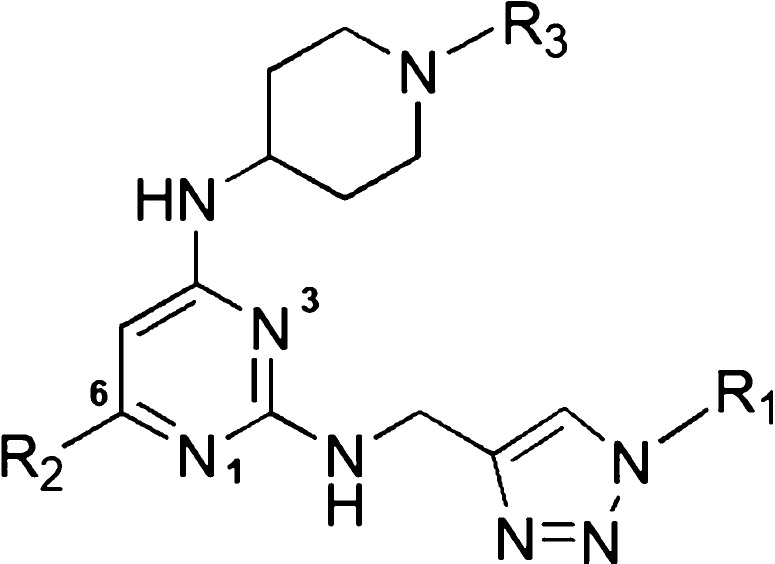

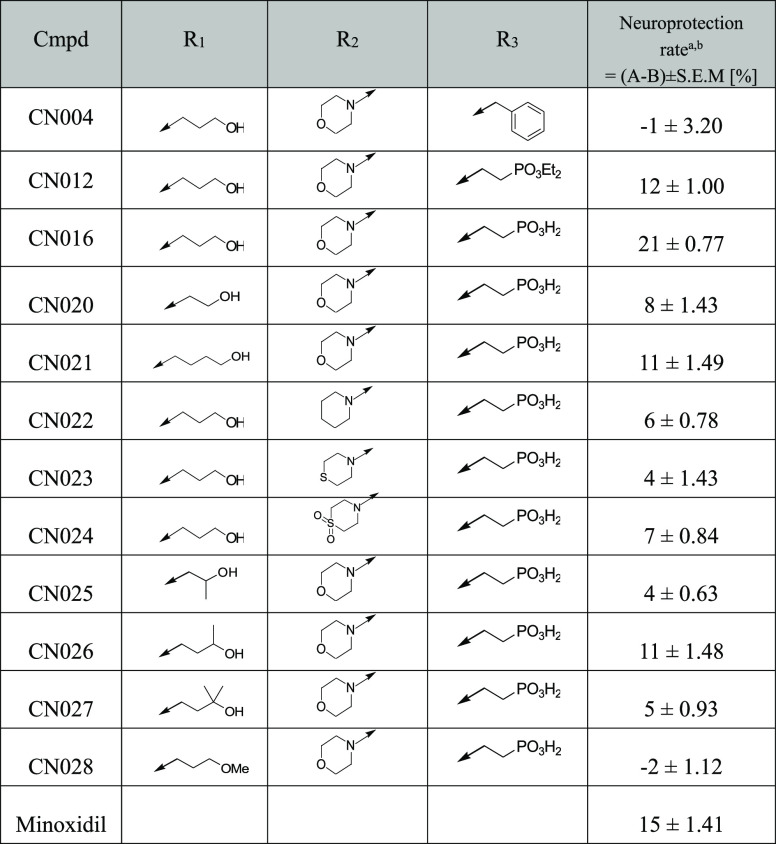
Neurite Outgrowth Assay of Lead Optimization Compounds

a A: Neurite outgrowth (% of control) of a test compound (1000 nM)+paclitaxel (50 nM); B: neurite outgrowth (% of control) of paclitaxel (50 nM) alone.

b By the neurite outgrowth assay, a neuroprotection rate represents the difference in neurite length between a tested compound/paclitaxel-treated group and a paclitaxel-treated alone group on DRG neurons. All experiments were performed in triplicate.

These intermediates were then individually coupled with an amine (i.e., morpholine) at 120 °C in 1-pentanol to furnish the corresponding C6-substituted compounds in 65–89% yields, which were subsequently subjected to hydrogenolysis to afford the corresponding amines in 88–93% yields. Each of these amine intermediates could undergo Michael addition with diethyl vinylphosphonate smoothly to yield diethyl phosphonates in 61–70% yields, of which diethyl moieties were removed under standard conditions (i.e., TMSBr) to achieve final products CN016 and CN020–CN028 as a hydrobromide salt in 82–90% yields. Indeed, the synthetic route of each final compound is provided in the Supporting Information. These compounds were then evaluated in the neurite outgrowth assay using minoxidil as a positive control. Results are compiled in Table 1, and thereof, SAR discussion is described below. When the length of R_1_ was extended or shortened by one methylene unit, the resulting CN020 and CN021, while exerting significant 8–11% protection rates in the neurite outgrowth assay, were far inferior to the parent CN016 (21%). Thus, a three-methylene unit for the R_1_ in the C-2 linker is tentatively thought to be optimal. R_2_ was then subjected to modification by replacing a morpholine unit with different six-membered rings. As a result, CN022–CN024, though possessing a moderate neuroprotection rate (4–7%), remain inferior to the corresponding CN016, suggesting that a protruding oxygen atom in the morpholine moiety might play a critical role in exerting neuroprotective effects. Previous SAR showed that the hydroxyl group at the terminal of the C-2 linker is more favorable than a hydrophobic group or an amine moiety. Thus, we maintained the hydroxyl group at the same position and created the corresponding secondary and tertiary alcohols CN025–027, of which neuroprotection rates (4–11%) were found to be significantly reduced as compared to the primary alcohol CN016 (21%), suggesting that the hydroxyl group at the terminal might be the most appropriate position for exerting activity. In addition, when the terminal hydroxyl group was capped by a methyl moiety, the resulting CN028, containing a methoxy terminus, was devoid of neuroprotective effects (−2% compared to paclitaxel treatment), again supporting that the SAR observed in Figure 2 (e.g., CN009 vs CN010) is reliable. Though analogues designed during lead optimization are limited, the SAR can be well established based on 34 compounds listed in Figures 1 and 2 and Table 1, in which a morpholine unit at the C-6 position in the pyrimidine ring and a phosphonate and hydroxyl moiety, respectively, placed at the terminal of linkers at C-2 and C-4 positions are essential for exhibiting nueroprotective activities. Indeed, CN016 (21 ± 0.77%), possessing a significantly higher neuroprotection rate than minoxidil (15 ± 1.41%), is extremely rare and hard to attain, particularly in terms of the neurite outgrowth assay on DRG neurons.

### CN016 Ameliorates PIPN In Vivo

**Figure 4:**
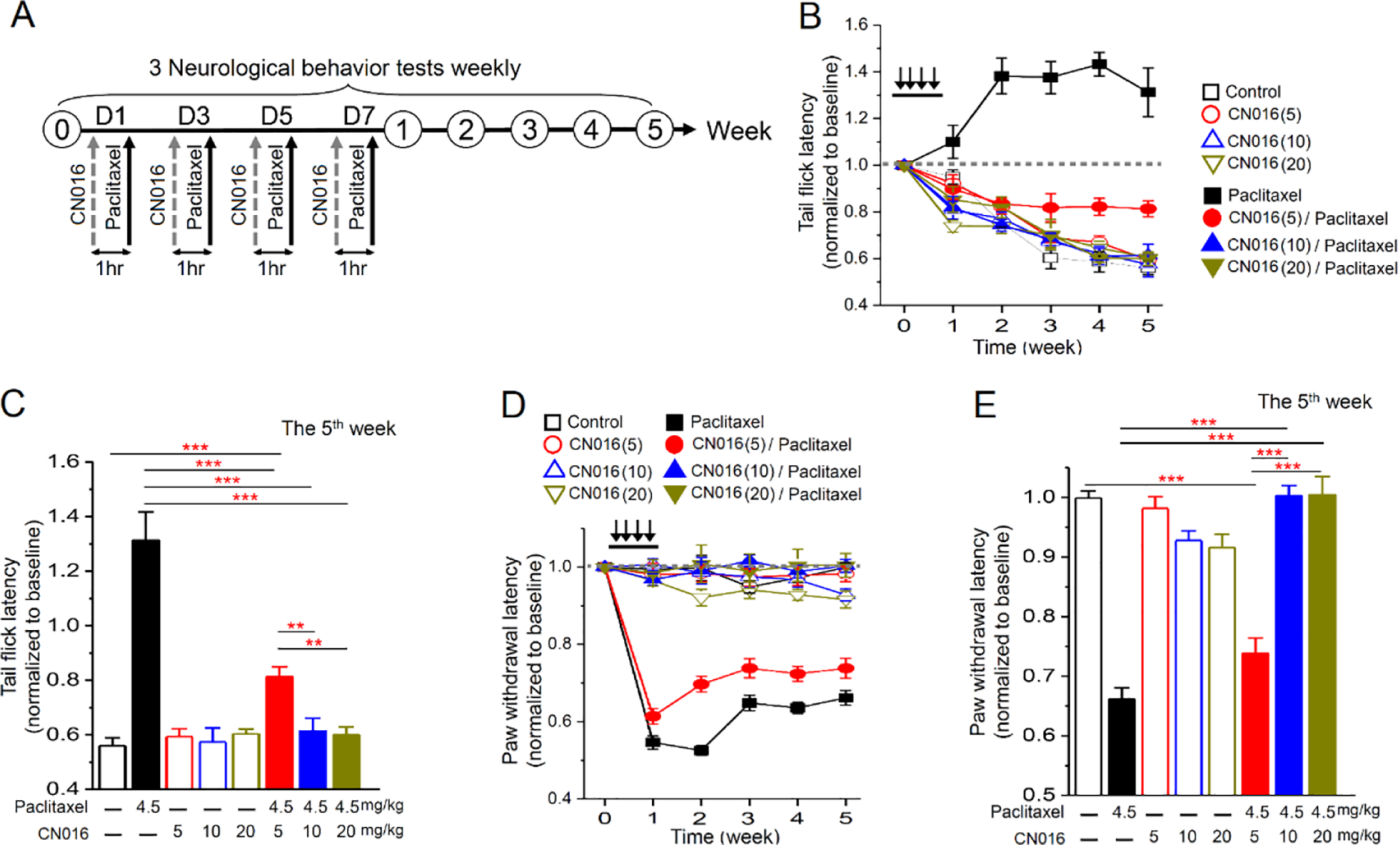
CN016 relieved paclitaxel-induced neuropathy in mice. (A) Experimental protocol of neurological behavior tests and drug administration. The basal levels of every neurological test were taken before treatment. In the first week, CN016 (5, 10, and 20 mg/kg) was administrated by IP 1 h prior to each injection of paclitaxel (4.5 mg/kg). After four courses of treatment, three behavioral tests were conducted weekly for five weeks. (B) Tail immersion test for the assessment of thermal sensation. *Y*-axis, the normalized baseline of latency from tail immersion to tail flick. Black arrows indicate the infusion of the drug. (C) Quantitative analyses of tail-flick latency at the fifth week. Each value represents mean ± S.E.M. from at least five mice in each group. Statistical significance was analyzed using two-way ANOVA. **P < 0.01; ***P < 0.001. (D) von Frey filament test for the detection of the mechanical pressure pain thresholds. *Y*-axis, normalized pressure from touch to paw withdrawal. Black arrows indicate the infusion of the drug. (E) Quantitative analyses of paw withdrawal threshold at the fifth week. Each value represents mean ± S.E.M. from at least five mice in each group. Statistical significance was analyzed using two-way ANOVA. ***P < 0.001.

Three behavioral models of paclitaxel-induced neuropathy were used to verify the neuroprotective effect of CN016. Namely, thermal and mechanical nociceptive reactions described below were evaluated by tail immersion and von Frey filament tests, respectively, and motor coordination was assessed with the rotarod performance test (Supporting Information, Figure S1A,B). To mimic the clinical therapeutic regimen, CN016 (5, 10 and 20 mg/kg) was administrated to 7-week-old female C57BL/6 mice by intraperitoneal injection (IP) 1 h prior to each injection of paclitaxel (4.5 mg/kg) on four alternate days (days 1, 3, 5, and 7). Each test’s basal level was recorded before treatment, and additional sessions of behavior tests were performed weekly for five weeks (Figure 4A).

After four courses of treatment, the mice treated with paclitaxel were found to decrease sensibility to high temperature (Figure 4B,C) and caused mechanical hypersensitivity (Figure 4D,E), highly consistent with the clinical neurological symptoms developed in paclitaxel-treated cancer patients. In sharp contrast, pretreatment with CN016 at a high dose (10 or 20 mg/kg) was able to alleviate both paclitaxel-induced thermhypesthesia (Figure 4B,C) and mechanical allodynia (Figure 4D,E) significantly in the same experiments. However, CN016 administered at a low dose (5 mg/kg) only moderately alleviated thermal insensitivity and mechanical allodynia.

### CN016 Protects Small Fibers from Damage Triggered by Paclitaxel

**Figure 5:**
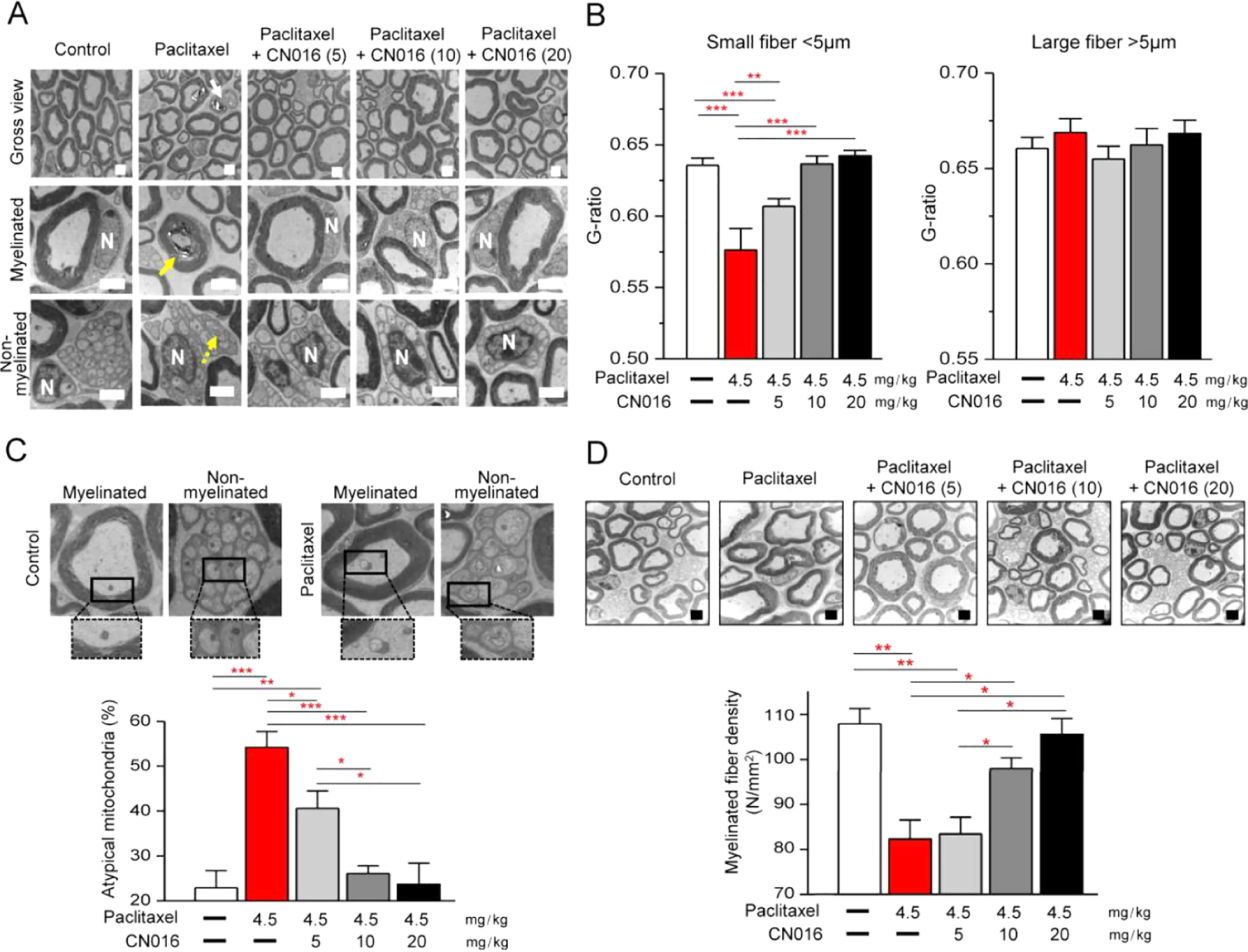
CN016 prevented paclitaxel-induced sciatic nerve damage. (A) Representative high-resolution images of sciatic nerves from different treated mice scanned by TEM. White arrow, degenerated axon. Yellow arrow, demyelinated axon. Yellow dashed arrow, swollen intra-axonal mitochondria. N, nucleus of Schwann cell. Scale bar, 2 μm. (B) Statistical bar charts of G-ratio in mouse sciatic nerves. The myelinated axons are classified into small fibers (axon diameter < 5 μm) and large fibers (axon diameter > 5 μm). Each value represents mean ± S.E.M. of at least 100 fibers. Statistical significance was analyzed using two-tailed Student’s *t*-test. **P < 0.01; ***P < 0.001. (C) Representative TEM images for mitochondrial morphology in cross-section of the sciatic nerve from control and paclitaxel treatment group (top). Quantitative analysis of the proportion of atypical mitochondria in axons, including myelinated and nonmyelinated fibers (bottom). Each value represents mean ± S.E.M. of at least 100 mitochondria. Statistical significance was analyzed using two-tailed Student’s *t*-test. *P < 0.05; **P < 0.01; ***P < 0.001. (D) Gross views for axon distribution in cross-section of the sciatic nerve (top). Scale bar, 4 μm. Quantitative analysis of the sciatic nerve myelinated fiber density (bottom). Each value represents mean ± S.E.M. of at least six different samples. Statistical significance was analyzed using two-tailed Student’s *t*-test. *P < 0.05; **P < 0.01.

Sciatic nerves were isolated from mice after drug treatment and behavioral tests to examine the ultrastructure of nerve fibers. As shown in Figure 5A, paclitaxel caused both myelinated and nonmyelinated fiber damage, in which nerve tissues with a shrinking axon were surrounded by myelin debris (white arrow), degenerated axon with collapsed myelin lamellae (yellow arrow), as well as swollen and vacuolated intra-axonal mitochondria (yellow dashed arrow). On the contrary, pretreatment with CN016 palliated the observed nerve damage. To evaluate the integrity of myelin, we calculated the G-ratio (axon perimeter/myelin perimeter) in different treatment groups. Because the symptoms of PIPN mostly manifest with sensory impairment,^28^ we roughly classified nerve fibers into larger fibers (diameter > 5 μm) and small fibers (diameter < 5 μm) depending on the type of sensory information conveyed.^29^ Large fibers with greater than 5 μm diameter carry information related to proprioception, touch, and pressure. In contrast, small fibers with less than 5 μm diameter carry information related to temperature and pain. Results showed that the G-ratio declined significantly in small fibers in paclitaxel-treated mice, whereas pretreated CN016 could rescue the observed axonal damage, particularly, at a dose of 10 or 20 mg/kg (Figure 5B).

On the other hand, paclitaxel treatment alone or along with pretreated CN016 at different dosages (5, 10, and 20 mg/kg) did not affect the integrity of large fibers (Figure 5B). In addition, we also found that paclitaxel could render mitochondria swollen and vacuolated (atypical mitochondria) in sciatic nerve tissues, but the proportion of this atypical mitochondria was significantly decreased by pretreatment of CN016 (Figure 5C). Quantitative analysis of myelinated fiber densities also revealed that a 25% fiber loss caused by paclitaxel could be significantly restored by CN016 in a dose-dependent manner, particularly at a dose of 20 mg/kg (Figure 5D).

The above findings also provide in-depth evidence to explain why pretreated CN016 could significantly reduce thermal insensitivity and mechanical pressure pain in neurological behavior tests as seen in Figure 4.

### CN016 Inhibits Inflammatory Responses to Attenuate PIPN

**Figure 6:**
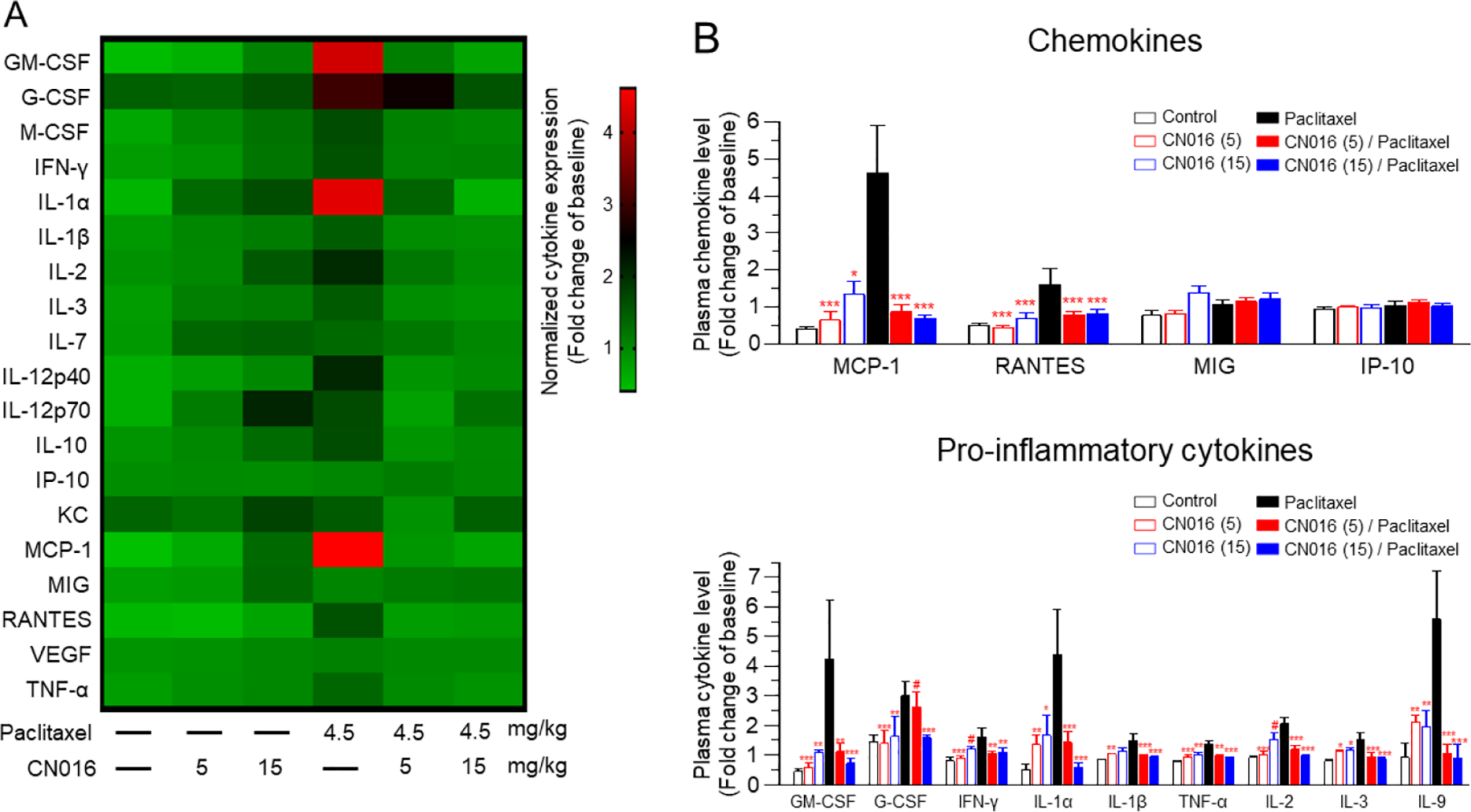
CN016 significantly diminished paclitaxel-induced systemic inflammation. (A) Heat map of 19 plasma cytokines profiled for all exposure treatments on day 14 after the first paclitaxel injection. The color scale represents scaled expression values. Red indicates high expression, while green indicates low expression levels. (B) Serum levels of chemokines (top) and proinflammatory cytokines (bottom panel, *n* = 5 mouse/group) on day 14 after the first course of treatment. Data are presented as the mean ± S.E.M. Statistical significance was analyzed using two-way ANOVA. ^#^*P* < 0.05 vs vehicle group; *P < 0.05; **P < 0.01; ***P < 0.001 vs paclitaxel group.

Neuroinflammation is regarded as a mechanism participating in the development of chemotherapy-induced peripheral neuropathy (CIPN).^30,31^ Previous studies showed that paclitaxel could drive M1 macrophage activation and infiltration into DRG, and subsequent inflammatory responses occurred. Thus, inhibiting this inflammatory cascade might ameliorate paclitaxel-induced neuropathy.^32,33^ In paclitaxel-treated mice (Figure 6A,B), we found that chemokines, MCP-1 and RANTES, and proinflammatory cytokines, GM-CSF, G-CSF, IFN-γ, IL-1α, IL-1β, TNF-α, IL-2, IL-3, and IL-9, were increased.^11,28,34,35^ In contrast, pretreatment with CN016 resulted in a significant decrease in many of proinflammatory cytokines and chemokines as examined above, implying that mechanistically, the observed neuroprotective effects might be associated with the suppression of proinflammatory markers.

**Figure 7:**
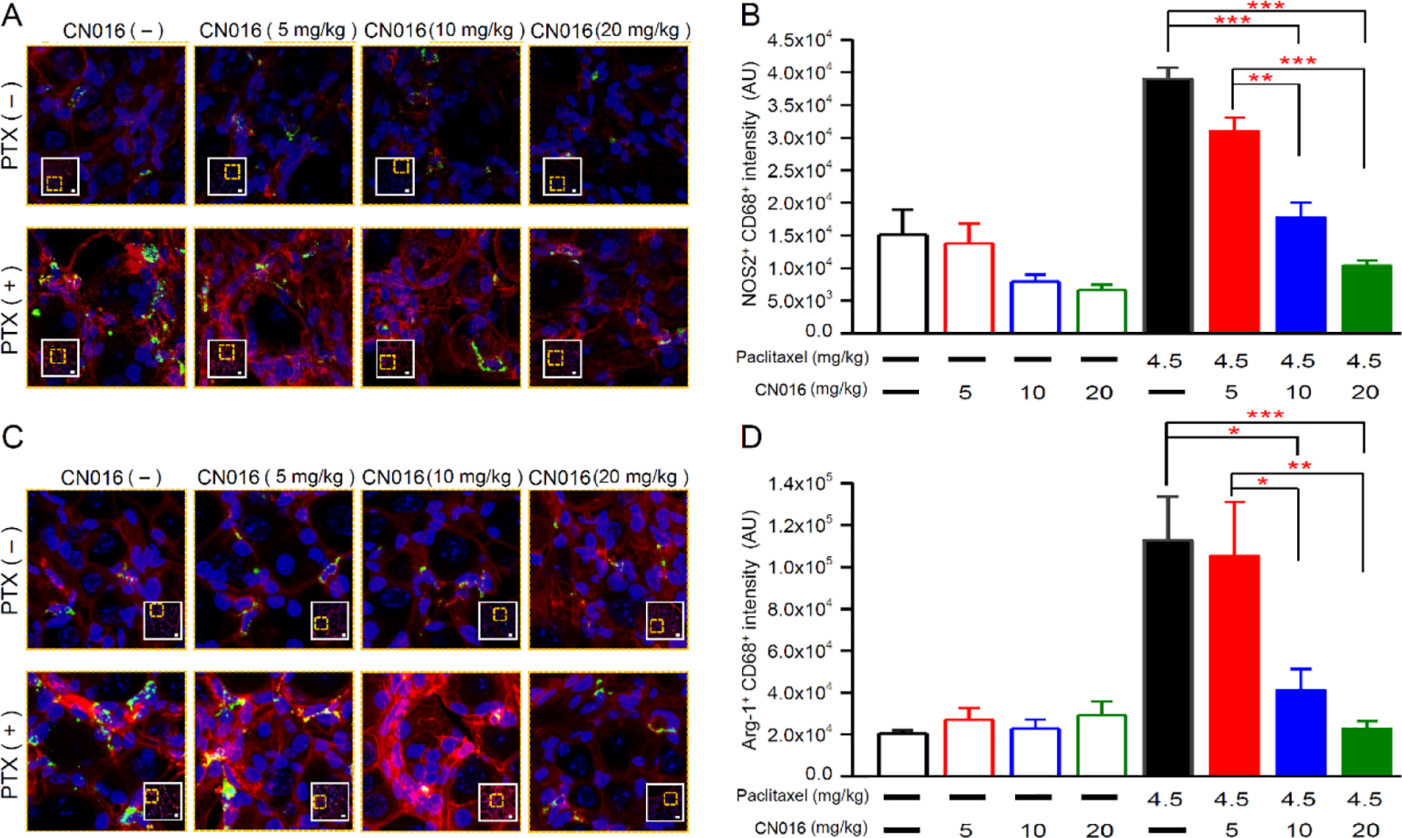
CN016 blocked proinflammatory macrophages recruited by paclitaxel in DRG tissues. (A) Magnified representative images of boxed regions showing the double-immunofluorescence staining for CD68 (macrophage marker; green) and NOS2 (M1-like/proinflammatory marker; red) in DRG tissues on the seventh day after the first course of treatment. Scale bar, 20 μm. (B) Number of M1 phenotype (NOS2^+^/CD68^+^) macrophage was analyzed in DRG tissues on the seventh day after the first course of treatment. Data are presented as the mean ± S.E.M. Statistical significance was analyzed using one-way ANOVA and Bonferroni post-test. **P < 0.01; ***P < 0.001. (C) Magnified representative images of boxed regions showing the double-immunofluorescence staining for CD68 (macrophage marker; green) and Arg-1 (M2-like/anti-inflammatory marker; red) in DRG tissues on the seventh day after the first course of treatment. Scale bar, 20 μm. (D) Number of M2 phenotype (Arg-1^+^/CD68^+^) macrophages was analyzed in DRG tissues on the seventh day after the first course of treatment. Data are presented as the mean ± S.E.M. Statistical significance was analyzed using one-way ANOVA and Bonferroni post-test. *P < 0.05; **P < 0.01; ***P < 0.001.

The confocal images of DRG tissues displayed a large number of M1 and M2 macrophage infiltration into the DRG tissue on the seventh day after first paclitaxel injection. Injection of a high dose of CN016 (10 or 20 mg/kg) before paclitaxel treatment significantly diminished the infiltration of M1 and M2 macrophages in DRG tissues but failed to inhibit paclitaxel-induced M1 and M2 macrophages recruitment at a low dosage (Figure 7).

**Figure 8:**
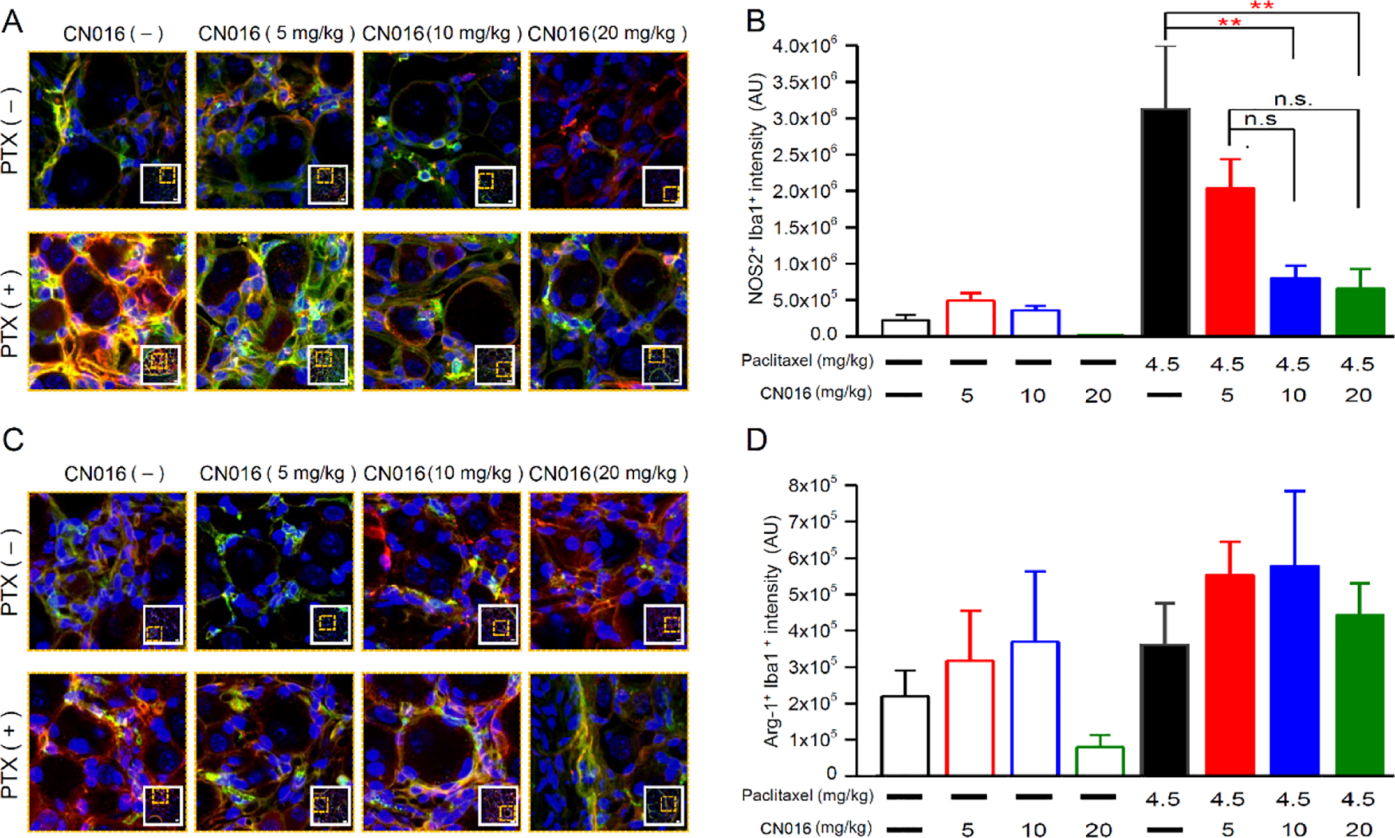
Paclitaxel recruited M1 dominant microglia in DRG tissues suppressed by CN016. (A) Magnified representative images of boxed regions showing the double-immunofluorescence staining for Iba1 (microglia marker; red) and NOS2 (M1-like/proinflammatory marker; green) in DRG tissues on the seventh day after the first course of treatment. Scale bar, 20 μm. (B) Number of M1 phenotype (NOS2^+^/Iba1^+^) microglia was analyzed in DRG tissues on the seventh day after the first course of treatment. Data are presented as the mean ± S.E.M. Statistical significance was analyzed using one-way ANOVA and Bonferroni post-test. **P < 0.01. (C) Magnified representative images of boxed regions showing the double-immunofluorescence staining for Iba1 (microglia marker; red) and Arg-1 (M2-like/anti-inflammatory marker; green) in DRG tissues on the seventh day after the first course of treatment. Scale bar, 20 μm. (D) Number of M2 phenotype (Arg-1^+^/Iba1^+^) microglia was analyzed in DRG tissues on the seventh day after the first course of treatment. Data are presented as the mean ± S.E.M. Statistical significance was analyzed using one-way ANOVA and Bonferroni post-test.

Besides the macrophage subsets, microglia might also contribute to the development of neuropathy after peripheral nerve damage.^36,37^ As depicted in Figure 8, the mice that received CN016 could dramatically reduce paclitaxel-induced activation of M1 microglia in DRG neurons, suggesting that the paclitaxel-triggered inflammation response in sensory neurons appeared to be significantly inhibited.

### Toxicology, Pharmacokinetics, Target Searching, and Vital Sign Studies on CN016

An acute toxicity study (Table 2) revealed that CN016 showed an extremely high maximum tolerance dose (MTD > 500 mg/kg) following intravenous administration in ICR mice, indicating that it may have a large therapeutic window (MTD/MED > 50) relative to the minimum efficacy dose (MED = 10 mg/kg). Pharmacokinetic studies on CN016 following intravenous injection (IV, 5 mg/kg) and IP (5 mg/kg) were also performed. Good blood exposure (AUC = 6985 ng/mL·h) and long duration time (*T*_1/2_ = 12.8 h) were found following IV injection (Table 2), implying that it might have a promising therapeutic utility clinically. More importantly, pharmokinetic analysis (see Figure S2) following IP administration revealed that the plasma concentration of CN016 (84 nM) was more than 8 times higher than in vitro effective concentration (10 nM), again rationalizing the high efficacy observed in animal neurological behavior tests seen in Figure 4.

**Table 2:** Single-Dose Toxicity and Pharmacokinetic Studies on CN016^a^

dose (mg/kg)	route	survival	survival rate	vehicle
50	IV	3/3	100%	saline
100	IV	6/6	100%	saline
300	IV	3/3	100%	saline
500	IV	3/3	100%	saline

sose (mg/kg), IV	*T*_1/2_ (h)	clearance (mL/min/kg)	*V*_ss_ (L/kg)	AUC(0–24) (ng/mL·h)
5	12.8 ± 3.4	12.8 ± 2.9	0.8 ± 0.5	6985 ± 1850

a Values indicate mean ± SD (*n* = 3) following IV in ICR mice.

In the safety assessment, neither paclitaxel treatment nor pretreated CN016 affected motor coordination as indicated in the rotarod test (Supporting Information, Figure S1A,B). We further monitored the mouse systolic pressure, heart rate, and body weight following the protocol as indicated in Figure S1C, the results of which suggested that mice treated with CN016 and paclitaxel did not cause heart rhythm variability, systolic blood pressure fluctuations, and weight change (Figure S1D–F). Moreover, hERG liability, mainly causing sudden death because of arrhythmia, was not observed for CN016 at a concentration up to 100 μM in the patch-clamp assay (Supporting Information, Table S3). Testing CN016 on six human liver microsomal CYP450 enzymes, including 1A2, 2C9, 2C19, 2D6, 2E1, and 3A4, was also conducted. Results showed that no substantial cytochrome inhibition (IC_50_ > 100 μM) was observed, thus minimizing the potential for CYP-induced drug–drug interactions accompanied by CN016 (Table S4). To identify an unknown target(s), CN016 was attempted to screen over a panel of selected 67 commercially available receptors and enzymes, but results showed that a very low inhibitory activity (<20% at 10 μM, Table S5) was detected for each tested item, suggesting that an authentic biological target remains to be determined.

### Comparing In Vivo Efficacy of CN016 with Marketed Minoxidil

According to our previous study,^20^ minoxidil caused tachycardia (25 mg/kg) and hypotension (50 mg/kg) adverse effects in mice and showed no protective effect on paclitaxel-induced neuropathy in behavior tests up to 25 mg/kg. On the contrary, CN016 exhibited a minimum effective dose at 5 mg/kg, and no harmful effects, including heart rhythm, systolic blood pressure, and weight change (Figure S1D–F), were observed at a dose of 20 mg/kg. Based on the above findings, CN016 is highly expected to be a superior neuroprotective drug candidate against PIPN.

## Conclusions

So far, due to the intricacy of chemical-induced peripheral neuropathy, there is no effective clinical agent that exerts a substantial benefit for the prevention or treatment of the side effects. This study leads to identifying a novel compound CN016 that exhibits neuroprotective capacity against PIPN as demonstrated by the significant recovery of thermal dysesthesia and mechanical hypersensitivity in neuropathy mouse models. CN016 appears to effectively inhibit paclitaxel-induced inflammatory responses and the infiltration of immune cells into sensory neurons. Thus, suppressing proinflammatory effects may explain, in part, the mechanism of action of CN016 on alleviating PIPN; however, disclosing the underlying signaling pathway remains warranted. Based on excellent efficacy in improving behavioral functions, high safety profiles (MTD > 500 mg/kg), and a large therapeutic window (MTD/MED > 50) in mice, CN016 might have great potential to become a peripherally neuroprotective agent to prevent neurotoxicity caused by chemotherapeutics as typified by paclitaxel.

## Experimental Section

### Chemistry

Paclitaxel was purchased from Sinphar Pharmaceutical Co. Ltd. (Taiwan). Minoxidil (SC-200987A, Santa-Cruz, USA) was dissolved in dimethyl sulfoxide (DMSO). The compounds used for neuroprotective screening were synthesized and dissolved in DMSO or double-distilled water (ddH_2_O) at 10 mM as a stock solution. The chemicals in our library were purchased from ChemDiv, ChemBridge, TimTec, MCE, and APExBIO.

### General Information

Unless otherwise stated, all materials used were commercially available and used as supplied. Reactions requiring anhydrous conditions were performed in flame-dried glassware and cooled under an argon or nitrogen atmosphere. Unless otherwise stated, the reactions were carried out under argon or nitrogen and monitored by analytical thin-layer chromatography performed on glass-backed plates (5 × 10 cm) precoated with silica gel 60 F254 as supplied by Merck. Visualization of the resulting chromatograms was performed by looking under an ultraviolet lamp (λ = 254 nm) followed by dipping in an ethanol solution of vanillin (5% w/v) containing sulfuric acid (3% v/v) or phosphomolybdic acid (2.5% w/v) and charring with a heat gun. Flash chromatography was used routinely for purification and separation of product mixtures using silica gel 60 of 230–400 mesh size as supplied by Merck. Eluent systems are given in volume/volume concentrations. ^1^H and ^13^C NMR spectra were recorded on Bruker Ascend 400 (400 MHz) and Bruker-Ascend 600 (600 MHz) spectrometers. Chloroform-*d*, methanol-*d*_4_, or deuterium oxide-*d*_2_ was used as the solvent and TMS (δ 0.00 ppm) as an internal standard. Chemical shift values are reported in ppm relative to the TMS in delta (δ) units. Multiplicities are recorded as s (singlet), br s (broad singlet), d (doublet), t (triplet), q (quartet), dd (doublet-of-doublets), dt (doublet-of-triplets), and m (multiplet). Coupling constants (*J*) are expressed in hertz. Electrospray mass spectra (ESMS) were recorded as *m*/*z* values using an Agilent 6125B single quadrupole LC/MS spectrometer, and HRMS-ESI mass were detected by a VARIAN 901-MS (FT-ICR Mass) spectrometer. All test compounds displayed more than 95% purity as determined by an Agilent 1100 series HPLC system using a C18 column (Thermo Scientific, Hypersil Golden, 4.6 mm × 250 mm). The gradient system for HPLC separation was composed of MeOH (mobile phase A) and H_2_O solution containing 0.1% trifluoroacetic acid (mobile phase B). The starting flow rate was 1 mL/min, and the injection volume was 10 μL. During the first 2 min, the percentage of phase A was 10%. At 6 min, the percentage of phase A was increased to 50%. At 16 min, the percentage of phase A was increased to 90% over 9 min. The system was operated at 25 °C. Peaks were detected at λ = 254 nm. IUPAC nomenclature of compounds was recorded with ACD/Name Pro software. All novel compounds reported here were screened for PAINS using KNIME: PAINS-Indigo (module) software, and the results showed that no PAINS liability was detected for them.^38^

### Flowchart of Screening 150,000 Compounds Based on CSVOA024807 Substructure

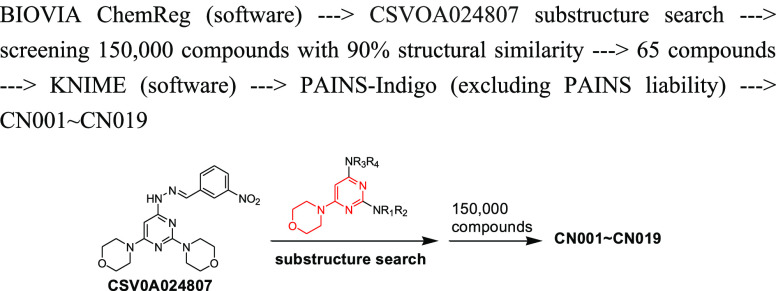



### General Synthetic Procedure for Lead Optimization Compounds Using Various Triazole Linkers in Scheme 1

#### (1-Benzyl-piperidin-4-yl)-(2,6-dichloro-pyrimidin-4-yl)-amine (CN016-1)

To the solution of 2,4,6-trichloropyrimidine (5.40 g, 29.44 mmol) in THF (150 mL) were added 1-benzyl-piperidin-4-ylamine (6.31 g, 33.16 mmol) and TEA (4.52 g, 44.67 mmol) under an atmosphere of nitrogen. The mixture was stirred at 60 °C for 15 h and then quenched with NH_4_Cl(aq). The resulting mixture was extracted with ethyl acetate. The combined organic extracts were washed with brine, dried over anhydrous sodium sulfate, filtered, and concentrated. The residue thus obtained was purified by column chromatography on silica gel (*n*-hexane/ethyl acetate = 1:4) to afford CN016-1 (5.60 g, 56%). ^1^H NMR (600 MHz, CDCl_3_): δ 7.34–7.24 (m, 5H), 6.26 (s, 1H), 3.54 (s, 2H), 2.85 (m, 2H), 2.19 (m, 2H), 1.99 (m, 2H), 1.57 (m, 2H). ^13^C NMR (150 MHz, CDCl_3_): δ 163.4, 161.0, 160.0, 158.7, 137.9, 129.2, 128.4, 127.2, 102.8, 98.8, 63.0, 51.8, 49.0, 48.3, 31.8. ESMS *m*/*z*: 337.1 [M + H]^+^.

#### 3-(4-{[4-(1-Benzyl-piperidin-4-ylamino)-6-chloro-pyrimidin-2-ylamino]-methyl}-[1,2,3]triazol-1-yl)-propan-1-ol (CN016-2)

A solution of CN016-1 (4.01 g, 11.89 mmol) and 3-(4-aminomethyl-[1,2,3]triazol-1-yl)-propan-1-ol (2.16 g, 13.83 mmol) in 1-pentanol (80 mL) was heated at 145 °C for 15 h. The resulting mixture was concentrated, and the residue thus obtained was purified by column chromatography on silica gel (MeOH/ethyl acetate = 1:9) to afford CN016-2 (3.21 g, 64%). ^1^H NMR (600 MHz, CD_3_OD): δ 7.79 (s, 1H), 7.32–7.24 (m, 5H), 5.80 (s, 1H), 4.59 (s, 2H), 4.45 (t, *J* = 7.2 Hz, 2H), 3.80 (m, 1H), 3.54 (t, *J* = 6.0 Hz, 2H), 3.51 (s, 2H), 2.83 (m, 2H), 2.12 (m, 2H), 2.05 (m, 2H), 1.86 (m, 2H), 1.49 (m, 2H). ^13^C NMR (150 MHz, CDCl_3_): δ 163.2, 161.6, 160.0, 146.1, 137.9, 129.3, 128.3, 127.2, 122.5, 93.9, 91.4, 63.0, 58.2, 52.1, 48.3, 47.0, 36.9, 32.7, 31.9. ESMS *m*/*z*: 457.2 [M + H]^+^.

#### 3-(4-{[4-(1-Benzyl-piperidin-4-ylamino)-6-morpholin-4-yl-pyrimidin-2-ylamino]-methyl}-[1,2,3]triazol-1-yl)-propan-1-ol (CN004)

A solution of compound CN016-2 (3.21 g, 7.02 mmol) and morpholine (3.01 g, 34.44 mmol) in 1-pentanol (48 mL) was heated at 120 °C for 15 h. The resulting mixture was concentrated, and the residue thus obtained was purified by flash chromatography on silica gel (MeOH/ethyl acetate = 1:3) to afford CN004 (3.16 g, 89%). ^1^H NMR (600 MHz, CD_3_OD): δ 7.76 (s, 1H), 7.34–7.26 (m, 5H), 4.57 (s, 2H), 4.45 (t, *J* = 7.2 Hz, 2H), 3.70–3.64 (m, 5H), 3.55–3.52 (m, 4H), 3.41 (m, 4H), 2.86 (m, 2H), 2.18 (m, 2H), 2.04 (m, 2H), 1.92 (m, 2H), 1.50 (m, 2H). ^13^C NMR (150 MHz, CDCl_3_): δ 164.3, 163.4, 161.4, 147.1, 138.1, 129.2, 128.2, 127.1, 122.1, 73.3, 66.7, 63.1, 58.4, 52.2, 48.1, 46.9, 44.6, 37.1, 32.7, 32.4. ESMS *m*/*z*: 508.3 [M + H]^+^. HRMS (ESI) *m*/*z*: calcd for C_26_H_38_N_9_O_2_ [M + H]^+^, 508.3149; found, 508.3151. HPLC purity = 95.2%, *t*_R_ = 9.70 min.

#### 3-(4-{[4-Morpholin-4-yl-6-(piperidin-4-ylamino)-pyrimidin-2-ylamino]-methyl}-[1,2,3]triazol-1-yl)-propan-1-ol (CN016-3)

A solution of CN004 (3.16 g, 6.23 mmol) and 10% Pd/C (0.95 g) in 2-propanol (60 mL) was stirred under H_2_(g) (1 atm) at 60 °C for 15 h. The resulting mixture was filtered, and the filtrate was concentrated to afford CN016-3 (2.38 g, 92%). ^1^H NMR (600 MHz, CD_3_OD): δ 7.78 (s, 1H), 4.58 (s, 2H), 4.46 (t, *J* = 7.2 Hz, 2H), 3.76 (m, 1H), 3.70 (m, 4H), 3.55 (t, *J* = 6.0 Hz, 2H), 3.42 (m, 4H), 3.06 (m, 2H), 2.70 (m, 2H), 2.07 (m, 2H), 1.94 (m, 2H), 1.39 (m, 2H). ^13^C NMR (150 MHz, CDCl_3_): δ 164.3, 163.5, 161.5, 148.1, 122.1, 73.4, 66.7, 58.1, 48.2, 46.9, 45.2, 44.6, 37.1, 33.4, 32.8. ESMS *m*/*z*: 418.2 [M + H]^+^.

#### {2-[4-(2-{[1-(3-Hydroxy-propyl)-1*H*-[1,2,3]triazol-4-ylmethyl]-amino}-6-morpholin-4-yl-pyrimidin-4-ylamino)-piperidin-1-yl]-ethyl}-phosphonic Acid Diethyl Ester (CN012)

A solution of CN016-3 (2.38 g, 5.70 mmol), diethyl vinylphosphonate (1.88 g, 11.45 mmol), and TEA (0.07 g, 0.69 mmol) in MeOH (60 mL) was stirred at 60 °C for 16 h and then concentrated. The residue thus obtained was purified by flash chromatography on silica gel (MeOH/ethyl acetate = 3:7) to afford CN012 (2.32 g, 70%). ^1^H NMR (400 MHz, CDCl_3_): δ 7.50 (s, 1H), 4.90 (s, 1H), 4.57 (d, *J* = 6.0 Hz, 2H), 4.40 (t, *J* = 6.8 Hz, 2H), 4.05 (q, *J* = 7.2 Hz, 4H), 3.69 (m, 4H), 3.56–3.40 (m, 7H), 2.79 (m, 2H), 2.61 (m, 2H), 2.13 (m, 2H), 2.10–1.89 (m, 6H), 1.45 (m, 2H), 1.28 (t, *J* = 7.2 Hz, 6H). ^13^C NMR (150 MHz, CDCl_3_): δ 164.2, 163.1, 161.1, 146.9, 122.2, 73.2, 66.6, 61.7 (d, *J* = 6.5 Hz), 58.1, 51.7, 51.4, 47.8, 46.9, 44.6, 37.0, 32.8, 32.0, 23.6 (d, *J* = 138.2 Hz), 16.4 (d, *J* = 6.2 Hz). ^31^P NMR (243 MHz, CDCl_3_): δ 30.6. ESMS *m*/*z*: 582.3 [M + H]^+^. HRMS (ESI) *m*/*z*: calcd for C_25_H_44_N_9_Na_1_O_5_P [M + Na]^+^, 604.3101; found, 604.3105. HPLC purity = 96.6%, *t*_R_ = 9.25 min.

#### {2-[4-(2-{[1-(3-Hydroxy-propyl)-1*H*-[1,2,3]triazol-4-ylmethyl]-amino}-6-morpholin-4-yl-pyrimidin-4-ylamino)-piperidin-1-yl]-ethyl}-phosphonic Acid Hydrobromide Salt (CN016)

To a solution of CN012 (2.32 g, 3.99 mmol) in DCM (4.6 mL) was added TMSBr (3.66 g, 23.91 mmol). The mixture was stirred at 25 °C for 4 h and then quenched with IPA/ethyl acetate. The slurry solution was filtrated to afford CN016 (2.48 g, 90%). ^1^H NMR (400 MHz, CD_3_OD): δ 8.15 (s, 1H), 4.75 (s, 2H), 4.58 (t, *J* = 6.8 Hz, 2H), 4.11 (m, 1H), 3.80–3.60 (m, 10H), 3.58 (t, *J* = 6.0 Hz, 2H), 3.43 (m, 2H), 3.28 (m, 2H), 2.35–2.25 (m, 4H), 2.13 (m, 2H), 1.88 (m, 2H). ^13^C NMR (150 MHz, D_2_O): δ 161.5, 153.2, 152.1, 143.8, 125.0, 71.9, 66.0, 58.0, 51.7, 51.4, 48.5, 46.0, 44.9, 35.5, 31.6, 28.7, 22.8 (d, *J* = 132.8 Hz). ^31^P NMR (243 MHz, D_2_O): δ 20.2. ESMS *m*/*z*: 526.2 [M + H]^+^. HRMS (ESI) *m*/*z*: calcd for C_21_H_36_N_9_Na_1_O_5_P [M + Na]^+^, 548.2475; found, 548.2477. HPLC purity = 96.8%, *t*_R_ = 8.61 min.

### Synthetic Procedures for CN001–CN019 Described in Supporting Information

#### (4-{[4-(1-Benzyl-piperidin-4-ylamino)-6-morpholin-4-yl-pyrimidin-2-ylamino]-methyl}-[1,2,3]triazol-1-yl)-acetic Acid Hydrochloride Salt (CN001)

Starting from 2,4,6-trichloropyrimidine (250 mg, 1.36 mmol), CN001 (179 mg) was obtained in 24% yield over four steps. ^1^H NMR (400 MHz, CD_3_OD): δ 8.17 (s, 1H), 7.62–7.58 (m, 2H), 7.53–7.50 (m, 3H), 5.36 (s, 2H), 4.75 (s, 2H), 4.38 (s, 2H), 4.01 (m, 1H), 3.78–3.70 (m, 8H), 3.64 (m, 2H), 3.26 (m, 2H), 2.24 (m, 2H), 1.88 (m, 2H). ESMS *m*/*z*: 508.2 [M + H]^+^. HRMS (ESI) *m*/*z*: calcd for C_25_H_34_N_9_O_3_ [M + H]^+^, 508.2785; found, 508.2785. HPLC purity = 97.1%, *t*_R_ = 8.93 min.

#### 3-[3-(4-{[4-(1-Benzyl-piperidin-4-ylamino)-6-morpholin-4-yl-pyrimidin-2-ylamino]-methyl}-[1,2,3]triazol-1-yl)-propoxy]-propionic Acid Methyl Ester (CN002)

Starting from 2,4,6-trichloropyrimidine (250 mg, 1.36 mmol), CN002 (173 mg) was obtained in 21% yield over four steps. ^1^H NMR (300 MHz, CDCl_3_): δ 7.50 (s, 1H), 7.38–7.20 (m, 5H), 4.88 (d, *J* = 6.9 Hz, 1H), 4.63 (d, *J* = 6.0 Hz, 2H), 4.45 (t, *J* = 6.6 Hz, 2H), 3.92 (m, 1H), 3.75 (m, 4H), 3.70 (s, 3H), 3.60 (t, *J* = 6.0 Hz, 2H), 3.51 (s, 2H), 3.03 (m, 4H), 2.83 (m, 2H), 2.66 (t, *J* = 6.9 Hz, 2H), 2.45 (t, *J* = 6.9 Hz, 2H), 2.13 (m, 2H), 2.06 (m, 2H), 1.95 (m, 2H), 1.54 (m, 2H). ESMS *m*/*z*: 594.3 [M + H]^+^. HRMS (ESI) *m*/*z*: calcd for C_30_H_44_N_9_O_4_ [M + H]^+^, 594.3516; found, 594.3516. HPLC purity = 95.4%, *t*_R_ = 9.64 min.

#### N4-(1-Benzyl-piperidin-4-yl)-N2-[1-(3-methylsulfanyl-propyl)-1*H*-[1,2,3]triazol-4-ylmethyl]-6-morpholin-4-yl-pyrimidine-2,4-diamine (CN003)

Starting from 2,4,6-trichloropyrimidine (250 mg, 1.36 mmol), CN003 (124 mg) was obtained in 17% yield over five steps. ^1^H NMR (400 MHz, CDCl_3_): δ 7.46 (s, 1H), 7.32–7.24 (m, 5H), 4.94 (s, 1H), 4.65 (d, *J* = 6.0 Hz, 2H), 4.43 (t, *J* = 6.8 Hz, 2H), 3.74 (m, 4H), 3.54 (m, 1H), 3.52 (s, 2H), 3.48 (m, 4H), 2.81 (m, 2H), 2.46 (t, *J* = 6.9 Hz, 2H), 2.20–2.14 (m, 4H), 2.08 (s, 3H), 1.96 (m, 2H), 1.51 (m, 2H). ESMS *m*/*z*: 538.3 [M + H]^+^. HRMS (ESI) *m*/*z*: calcd for C_27_H_40_N_9_OS [M + H]^+^, 538.3077; found, 538.3078. HPLC purity = 97.4%, *t*_R_ = 9.59 min.

#### 3-[3-(4-{[4-Morpholin-4-yl-6-(1-naphthalen-2-ylmethyl-piperidin-4-ylamino)-pyrimidin-2-ylamino]-methyl}-[1,2,3]triazol-1-yl)-propoxy]-propionic Acid Methyl Ester (CN005)

Starting from 2,4,6-trichloropyrimidine (250 mg, 1.36 mmol), CN005 (128 mg) was obtained in 15% yield over six steps. ^1^H NMR ((400 MHz, CD_3_OD): δ 8.04 (s, 1H), 7.99–7.90 (m, 3H), 7.78 (s, 1H), 7.64 (d, *J* = 8.0 Hz, 1H), 7.56–7.53 (m, 2H), 4.56 (s, 2H), 4.42 (t, *J* = 6.8 Hz, 2H), 4.36 (s, 2H), 3.85 (m, 1H), 3.75 (m, 4H), 3.63 (s, 3H), 3.48 (t, *J* = 6.4 Hz, 2H), 3.37 (m, 2H), 3.03 (m, 2H), 3.01 (m, 4H), 2.70 (t, *J* = 7.2 Hz, 2H), 2.46 (t, *J* = 7.2 Hz, 2H), 2.08–2.01 (m, 4H), 1.78 (m, 2H). ESMS *m*/*z*: 644.3 [M + H]^+^. HRMS (ESI) *m*/*z*: calcd for C_34_H_46_N_9_O_4_ [M + H]^+^, 644.3673; found, 644.3672. HPLC purity = 95.6%, *t*_R_ = 9.67 min.

#### 1,3,4,5-Tetrahydroxy-cyclohexanecarboxylic Acid {3-[4-(2-{[1-(3-Amino-propyl)-1*H*-[1,2,3]triazol-4-ylmethyl]-amino}-6-morpholin-4-yl-pyrimidin-4-ylamino)-piperidin-1-yl]-3-oxo-propyl}-amide Hydrochloride Salt (CN006)

Starting from 2,4,6-trichloropyrimidine (250 mg, 1.36 mmol), CN006 (150 mg) was obtained in 15% yield over seven steps. ^1^H NMR (300 MHz, D_2_O): δ 7.90 (s, 1H), 4.52 (s, 2H), 4.40 (t, *J* = 7.2 Hz, 2H), 4.16–4.04 (m, 2H), 3.86 (m, 1H), 3.77 (m, 1H), 3.65–3.55 (m, 5H), 3.47–3.30 (m, 7H), 3.16 (m, 1H), 2.86 (m, 2H), 2.78 (m, 1H), 2.53 (m, 2H), 2.14 (m, 2H), 1.93–1.70 (m, 6H), 1.40 (m, 1H), 1.26 (m, 1H). ESMS *m*/*z*: 662.3 [M + H]^+^. HRMS (ESI) *m*/*z*: calcd for C_29_H_47_N_11_Na_1_O_7_ [M + Na]^+^, 684.3558; found, 684.3553. HPLC purity = 95.1%, *t*_R_ = 8.77 min.

#### 1,3,4,5-Tetrahydroxy-cyclohexanecarboxylic Acid (3-{4-[2-({1-[3-(2-Hydroxy-ethylamino)-propyl]-1*H*-[1,2,3]triazol-4-ylmethyl}-amino)-6-morpholin-4-yl-pyrimidin-4-ylamino]-piperidin-1-yl}-3-oxo-propyl)-amide Hydrochloride Salt (CN007)

Starting from 2,4,6-trichloropyrimidine (250 mg, 1.36 mmol), CN007 (164 mg) was obtained in 16% yield over seven steps. ^1^H NMR (400 MHz, D_2_O): δ 7.86 (s, 1H), 4.52 (s, 2H), 4.40 (t, *J* = 6.8 Hz, 2H), 4.11–4.04 (m, 2H), 3.84 (m, 1H), 3.81–3.78 (m, 3H), 3.66–3.30 (m, 12H), 3.18 (m, 1H), 3.10–2.96 (m, 4H), 2.78 (m, 1H), 2.58 (m, 2H), 2.20 (m, 2H), 1.90–1.64 (m, 6H), 1.41 (m, 2H). ESMS *m*/*z*: 706.3 [M + H]^+^. HRMS (ESI) *m*/*z*: calcd for C_31_H_51_N_11_Na_1_O_8_ [M + Na]^+^, 728.3820; found, 728.3823. HPLC purity = 95.5%, *t*_R_ = 8.67 min.

#### 1,3,4,5-Tetrahydroxy-cyclohexanecarboxylic Acid (3-{4-[2-({1-[2-(2-Amino-ethoxy)-ethyl]-1*H*-[1,2,3]triazol-4-ylmethyl}-amino)-6-morpholin-4-yl-pyrimidin-4-ylamino]-piperidin-1-yl}-3-oxo-propyl)-amide Hydrochloride Salt (CN008)

Starting from 2,4,6-trichloropyrimidine (250 mg, 1.36 mmol), CN008 (126 mg) was obtained in 12% yield over seven steps. ^1^H NMR (400 MHz, D_2_O): δ 7.86 (s, 1H), 4.53 (s, 2H), 4.47 (t, *J* = 7.2 Hz, 2H), 4.16–4.05 (m, 2H), 3.88 (m, 1H), 3.81 (t, *J* = 5.2 Hz, 2H), 3.77 (m, 1H), 3.66 (m, 1H), 3.64–3.60 (m, 5H), 3.55 (t, *J* = 5.2 Hz, 2H), 3.48–3.30 (m, 6H), 3.15 (m, 1H), 3.00 (m, 2H), 2.79 (m, 1H), 2.56 (m, 2H), 1.93–1.70 (m, 6H), 1.40 (m, 1H), 1.26 (m, 1H). ESMS *m*/*z*: 692.3 [M + H]^+^. HRMS (ESI) *m*/*z*: calcd for C_30_H_50_N_11_O_8_ [M + H]^+^, 692.3844; found, 692.3843. HPLC purity = 95.9%, *t*_R_ = 8.72 min.

#### 3-[4-(2-{[1-(3-Hydroxy-propyl)-1*H*-[1,2,3]triazol-4-ylmethyl]-amino}-6-morpholin-4-yl-pyrimidin-4-ylamino)-piperidin-1-yl]-propionic Acid Methyl Ester (CN009)

Starting from 2,4,6-trichloropyrimidine (250 mg, 1.36 mmol), CN009 (129 mg) was obtained in 19% yield over five steps. ^1^H NMR (400 MHz, CDCl_3_): δ 7.53 (s, 1H), 4.93 (s, 1H), 4.66 (d, *J* = 6.0 Hz, 2H), 4.48 (t, *J* = 6.8 Hz, 2H), 3.75 (m, 4H), 3.70 (s, 3H), 3.60 (t, *J* = 6.0 Hz, 2H), 3.52–3.46 (m, 5H), 2.84 (m, 2H), 2.71 (t, *J* = 7.2 Hz, 2H), 2.52 (t, J = 7.2 Hz, 2H), 2.16 (m, 2H), 2.09 (m, 2H), 1.98 (m, 2H), 1.50 (m, 2H). ESMS *m*/*z*: 504.3 [M + H]^+^. HRMS (ESI) *m*/*z*: calcd for C_23_H_38_N_9_O_4_ [M + H]^+^, 504.3047; found, 504.3044. HPLC purity = 95.5%, *t*_R_ = 8.73 min.

#### 3-[4-(2-{[1-(3-Methoxy-propyl)-1*H*-[1,2,3]triazol-4-ylmethyl]-amino}-6-morpholin-4-yl-pyrimidin-4-ylamino)-piperidin-1-yl]-propionic Acid Methyl Ester (CN010)

Starting from 2,4,6-trichloropyrimidine (250 mg, 1.36 mmol), CN010 (125 mg) was obtained in 18% yield over five steps. ^1^H NMR (400 MHz, CDCl_3_): δ 7.55 (s, 1H), 4.93 (s, 1H), 4.68 (d, *J* = 6.0 Hz, 2H), 4.42 (t, *J* = 6.8 Hz, 2H), 3.74 (m, 4H), 3.60 (s, 3H), 3.56–3.52 (m, 5H), 3.35 (t, *J* = 6.0 Hz, 2H), 3.32 (s, 3H), 2.93 (m, 2H), 2.80 (t, *J* = 7.2 Hz, 2H), 2.57 (t, *J* = 7.2 Hz, 2H), 2.33 (m, 2H), 2.13 (m, 2H), 2.00 (m, 2H), 1.63 (m, 2H). ESMS *m*/*z*: 518.3 [M + H]^+^. HRMS (ESI) *m*/*z*: calcd for C_24_H_40_N_9_O_4_ [M + H]^+^, 518.3203; found, 518.3205. HPLC purity = 96.1%, *t*_R_ = 8.92 min.

#### 3-{3-[4-({4-[1-(2-Methoxycarbonyl-ethyl)-piperidin-4-ylamino]-6-morpholin-4-yl-pyrimidin-2-ylamino}-methyl)-[1,2,3]triazol-1-yl]-propoxy}-propionic Acid Methyl Ester (CN011)

Starting from 2,4,6-trichloropyrimidine (250 mg, 1.36 mmol), CN011 (102 mg) was obtained in 13% yield over six steps. ^1^H NMR (400 MHz, CDCl_3_) 7.53 (s, 1H), 4.92 (d, *J* = 7.6 Hz, 1H), 4.64 (d, *J* = 6.0 Hz, 2H), 4.48 (t, *J* = 6.8 Hz, 2H), 3.91 (m, 1H), 3.78 (m, 4H), 3.71 (s, 3H), 3.69 (s, 3H), 3.63 (t, *J* = 6.0 Hz, 2H), 3.05 (m, 4H), 2.84 (m, 2H), 2.73–2.65 (m, 4H), 2.54–2.48 (m, 4H), 2.21–2.10 (m, 4H), 1.97 (m, 2H), 1.49 (m, 2H). ESMS *m*/*z*: 590.3 [M + H]^+^. HRMS (ESI) *m*/*z*: calcd for C_27_H_44_N_9_O_6_ [M + H]^+^, 590.3415; found, 590.3416. HPLC purity = 97.5%, *t*_R_ = 8.46 min.

#### {2-[4-(2-{[1-(3-Hydroxy-3-methyl-butyl)-1*H*-[1,2,3]triazol-4-ylmethyl]-amino}-6-morpholin-4-yl-pyrimidin-4-ylamino)-piperidin-1-yl]-ethyl}-phosphonic Acid Diethyl Ester (CN013)

Starting from 2,4,6-trichloropyrimidine (250 mg, 1.36 mmol), CN013 (149 mg) was obtained in 18% yield over five steps. ^1^H NMR (400 MHz, CD_3_OD): δ 7.51 (s, 1H), 4.94 (s, 1H), 4.65 (d, *J* = 5.6 Hz, 2H), 4.47 (dd, *J* = 9.2, 6.8 Hz, 2H), 4.11 (q, *J* = 6.8 Hz, 4H), 3.74–3.65 (m, 5H), 3.49 (m, 4H), 2.90 (m, 2H), 2.67 (m, 2H), 2.21 (m, 2H), 2.11–1.93 (m, 6H), 1.52 (m, 2H), 1.33 (t, *J* = 6.8 Hz, 6H), 1.26 (s, 6H). ESMS *m*/*z*: 610.3 [M + H]^+^. HRMS (ESI) *m*/*z*: calcd for C_27_H_49_N_9_O_5_P [M + H]^+^, 610.3594; found, 610.3598. HPLC purity = 97.4%, *t*_R_ = 9.62 min.

#### {2-[4-(2-{[1-(3-Hydroxy-butyl)-1*H*-[1,2,3]triazol-4-ylmethyl]-amino}-6-morpholin-4-yl-pyrimidin-4-ylamino)-piperidin-1-yl]-ethyl}-phosphonic Acid Diethyl Ester (CN014)

Starting from 2,4,6-trichloropyrimidine (250 mg, 1.36 mmol), CN014 (136 mg) was obtained in 17% yield over five steps. ^1^H NMR (400 MHz, CD_3_OD): δ 7.88 (s, 1H), 4.57 (s, 2H), 4.47 (t, *J* = 7.2 Hz, 2H), 4.14 (q, *J* = 7.2 Hz, 4H), 3.75–3.65 (m, 6H), 3.42 (m, 4H), 2.91 (m, 2H), 2.64 (m, 2H), 2.21 (m, 2H), 2.07–1.86 (m, 6H), 1.51 (m, 2H), 1.35 (t, *J* = 7.2 Hz, 6H), 1.19 (d, *J* = 6.4 Hz, 3H). ESMS *m*/*z*: 596.3 [M + H]^+^. HRMS (ESI) *m*/*z*: calcd for C_26_H_47_N_9_O_5_P [M + H]^+^, 596.3438; found, 596.3439. HPLC purity = 95.3%, *t*_R_ = 9.20 min.

#### 3-(3-{4-[(4-{1-[2-(Diethoxy-phosphoryl)-ethyl]-piperidin-4-ylamino}-6-morpholin-4-yl-pyrimidin-2-ylamino)-methyl]-[1,2,3]triazol-1-yl}-propoxy)-propionic Acid Methyl Ester (CN015)

Starting from 2,4,6-trichloropyrimidine (250 mg, 1.36 mmol), CN015 (94 mg) was obtained in 10% yield over six steps. ^1^H NMR (300 MHz, CDCl_3_): δ 7.50 (s, 1H), 4.97 (d, *J* = 7.2 Hz, 1H), 4.62 (d, *J* = 5.7 Hz, 2H), 4.45 (t, *J* = 6.9 Hz, 2H), 4.07 (q, *J* = 6.9 Hz, 4H), 3.89 (m, 1H), 3.75 (m, 4H), 3.68 (s, 3H), 3.59 (t, *J* = 6.0 Hz, 2H), 3.02 (m, 4H), 2.85 (m, 2H), 2.76–2.60 (m, 4H), 2.47 (t, *J* = 6.9 Hz, 2H), 2.18 (m, 2H), 2.10–1.93 (m, 6H), 1.47 (m, 2H), 1.31 (t, *J* = 6.9 Hz, 6H). ESMS *m*/*z*: 668.3 [M + H]^+^. HRMS (ESI) *m*/*z*: calcd for C_29_H_51_N_9_O_7_P [M + H]^+^, 668.3649; found, 668.3651. HPLC purity = 96.2%, *t*_R_ = 9.29 min.

#### Methyl 3-(4-((2-((6-Hydroxyhexyl)amino)-6-morpholinopyrimidin-4-yl)amino)piperidin-1-yl)propanoate (CN017)

Starting from 2,4,6-trichloropyrimidine (250 mg, 1.36 mmol), CN017 (102 mg) was obtained in 16% yield over five steps. ^1^H NMR (400 MHz, CDCl_3_): δ 4.90 (s, 1H), 3.76 (m, 4H), 3.70 (s, 3H), 3.64 (t, *J* = 6.8 Hz, 2H), 3.50–3.47 (m, 5H), 3.33 (m, 2H), 2.84 (m, 2H), 2.71 (t, *J* = 7.2 Hz, 2H), 2.53 (t, *J* = 7.2 Hz, 2H), 2.19 (m, 2H), 2.00 (m, 2H), 1.59–1.39 (m, 10H). ESMS *m*/*z*: 465.3 [M + H]^+^. HRMS (ESI) *m*/*z*: calcd for C_23_H_41_N_6_O_4_ [M + H]^+^, 465.3189; found, 465.3192. HPLC purity = 95.8%, *t*_R_ = 9.74 min.

#### Diethyl(2-(4-((2-((4-(2-hydroxyethyl)benzyl)amino)-6-morpholinopyrimidin-4-yl)amino)piperidin-1-yl)ethyl)phosphonate (CN018)

Starting from 2,4,6-trichloropyrimidine (250 mg, 1.36 mmol), CN018 (136 mg) was obtained in 17% yield over five steps. ^1^H NMR (400 MHz, CDCl_3_): δ 7.30 (d, *J* = 8.0 Hz, 2H), 7.20 (d, *J* = 8.0 Hz, 2H), 4.93 (s, 1H), 4.54 (d, *J* = 5.6 Hz, 2H), 4.12 (q, *J* = 7.2 Hz, 4H), 3.86 (t, *J* = 5.2 Hz, 2H), 3.80 (m, 4H), 3.56 (m, 1H), 3.49 (m, 4H), 2.87–2.84 (m, 4H), 2.68 (m, 2H), 2.17 (m, 2H), 2.04–1.96 (m, 4H), 1.50 (m, 2H), 1.31 (t, *J* = 7.2 Hz, 6H). ESMS *m*/*z*: 577.3 [M + H]^+^. HRMS (ESI) *m*/*z*: calcd for C_28_H_45_N_6_Na_1_O_5_P [M + Na]^+^, 599.3087; found, 599.3087. HPLC purity = 97.7%, *t*_R_ = 9.98 min.

#### 2-(4-((2-((4-(2-Hydroxyethyl)benzyl)amino)-6-morpholinopyrimidin-4-yl)amino)piperidin-1-yl)ethyl)phosphonic Acid Hydrobromide Salt (CN019)

Starting from 2,4,6-trichloropyrimidine (250 mg, 1.36 mmol), CN019 (136 mg) was obtained in 15% yield over six steps. ^1^H NMR (400 MHz, CD_3_OD): δ 7.30 (d, *J* = 8.0 Hz, 2H), 7.23 (d, *J* = 8.0 Hz, 2H), 4.56 (br s, 2H), 4.11 (m, 1H), 3.75–3.65 (m, 12H), 3.45 (m, 2H), 3.31 (m, 2H), 2.82 (m, 2H), 2.35–2.25 (m, 4H), 1.90 (m, 2H). ESMS *m*/*z*: 521.2 [M + H]^+^. HRMS (ESI) *m*/*z*: calcd for C_24_H_37_N_6_Na_1_O_5_P [M + Na]^+^, 543.2461; found, 543.2462. HPLC purity = 95.5%, *t*_R_ = 9.88 min.

#### (2-(4-((2-(((1-(2-Hydroxyethyl)-1*H*-1,2,3-triazol-4-yl)methyl)amino)-6-morpholinopyrimidin-4-yl)amino)piperidin-1-yl)ethyl)phosphonic Acid Hydrobromide Salt (CN020)

Starting from 2,4,6-trichloropyrimidine (250 mg, 1.36 mmol), CN020 (149 mg) was obtained in 16% yield over six steps. ^1^H NMR (600 MHz, D_2_O): δ 8.08 (s, 1H), 4.62 (s, 2H), 4.48 (t, *J* = 5.4 Hz, 2H), 3.87 (t, *J* = 5.4 Hz, 2H), 3.80 (m, 1H), 3.70–3.40 (m, 10H), 3.28 (m, 2H), 3.07 (m, 2H), 2.25–2.10 (m, 4H), 1.69 (m, 2H). ^13^C NMR (150 MHz, D_2_O): δ 161.5, 153.2, 152.3, 144.6, 124.3, 71.7, 65.9, 59.7, 53.7, 51.6, 51.5, 46.0, 44.9, 35.4, 28.7, 22.7 (d, *J* = 133.4 Hz). ESMS *m*/*z*: 512.2 [M + H]^+^. HRMS (ESI) *m*/*z*: calcd for C_20_H_33_N_9_O_5_P [M + H]^+^, 512.2499; found, 512.2495. HPLC purity = 95.6%, *t*_R_ = 8.22 min.

#### {2-[4-(2-{[1-(4-Hydroxy-butyl)-1*H*-[1,2,3]triazol-4-ylmethyl]-amino}-6-morpholin-4-yl-pyrimidin-4-ylamino)-piperidin-1-yl]-ethyl}-phosphonic Acid Hydrobromide Salt (CN021)

Starting from 2,4,6-trichloropyrimidine (250 mg, 1.36 mmol), CN021 (157 mg) was obtained in 16% yield over six steps. ^1^H NMR (400 MHz, D_2_O): δ 7.87 (s, 1H), 4.55 (s, 2H), 4.33 (t, *J* = 6.8 Hz, 2H), 3.75 (m, 1H), 3.65–3.40 (m, 12H), 3.24 (m, 2H), 3.02 (m, 2H), 2.17 (m, 2H), 1.99 (m, 2H), 1.81 (m, 2H), 1.64 (m, 2H), 1.32 (m, 2H). ^13^C NMR (150 MHz, D_2_O): δ 161.2, 153.2, 152.2, 143.9, 124.8, 71.7, 65.9, 60.7, 51.7, 51.4, 51.3, 46.0, 44.9, 35.5, 28.7, 28.1, 25.8, 22.8 (d, *J* = 132.8 Hz). ESMS *m*/*z*: 540.2 [M + H]^+^. HRMS (ESI) *m*/*z*: calcd for C_22_H_39_N_9_O_5_P [M + H]^+^, 540.2812; found, 540.2810. HPLC purity = 95.4%, *t*_R_ = 8.80 min.

#### {2-[4-(2-{[1-(3-Hydroxy-propyl)-1*H*-[1,2,3]triazol-4-ylmethyl]-amino}-6-piperidin-1-yl-pyrimidin-4-ylamino)-piperidin-1-yl]-ethyl}-phosphonic Acid Hydrobromide Salt (CN022)

Starting from 2,4,6-trichloropyrimidine (250 mg, 1.36 mmol), CN022 (103 mg) was obtained in 11% yield over six steps. ^1^H NMR (400 MHz, CD_3_OD): δ 8.14 (s, 1H), 4.73 (s, 2H), 4.57 (t, *J* = 7.2 Hz, 2H), 4.11 (m, 1H), 3.75–3.59 (m, 6H), 3.58 (t, *J* = 6.0 Hz, 2H), 3.43 (m, 2H), 3.28 (m, 2H), 2.36–2.12 (m, 6H), 1.91 (m, 2H), 1.75–1.60 (m, 6H). ^13^C NMR (150 MHz, D_2_O): δ 161.8, 153.1, 152.4, 145.1, 123.9, 73.2, 58.0, 52.4, 51.4, 47.5, 46.5, 45.4, 36.0, 31.8, 28.8, 25.1, 23.7, 23.2 (d, *J* = 131.0 Hz). ESMS *m*/*z*: 524.3 [M + H]^+^. HRMS (ESI) *m*/*z*: calcd for C_22_H_39_N_9_O_4_P [M + H]^+^, 524.2863; found, 524.2867. HPLC purity = 95.5%, *t*_R_ = 9.78 min.

#### {2-[4-(2-{[1-(3-Hydroxy-propyl)-1*H*-[1,2,3]triazol-4-ylmethyl]-amino}-6-thiomorpholin-4-yl-pyrimidin-4-ylamino)-piperidin-1-yl]-ethyl}-phosphonic Acid Hydrobromide Salt (CN023)

Starting from 2,4,6-trichloropyrimidine (250 mg, 1.36 mmol), CN023 (95 mg) was obtained in 10% yield over six steps. ^1^H NMR (400 MHz, D_2_O): δ 7.91 (s, 1H), 4.54 (s, 2H), 4.39 (t, *J* = 6.8 Hz, 2H), 3.80–3.70 (m, 5H), 3.58 (m, 2H), 3.42 (t, *J* = 6.8 Hz, 2H), 3.27 (m, 2H), 3.04 (m, 2H), 2.50 (m, 4H), 2.17 (m, 2H), 2.06–1.98 (m, 4H), 1.68 (m, 2H). ^13^C NMR (150 MHz, D_2_O): δ 161.4, 154.0, 152.2, 144.9, 123.2, 72.1, 58.1, 52.2, 51.4, 48.0, 47.7, 46.0, 36.0, 31.7, 28.7, 26.0, 23.0 (d, *J* = 130.5 Hz). ESMS *m*/*z*: 542.2 [M + H]^+^. HRMS (ESI) *m*/*z*: calcd for C_21_H_36_N_9_Na_1_O_4_PS [M + Na]^+^, 564.2246; found, 564.2250. HPLC purity = 95.1%, *t*_R_ = 9.07 min.

#### {2-[4-(6-(1,1-Dioxo-1l6-thiomorpholin-4-yl)-2-{[1-(3-hydroxy-propyl)-1*H*-[1,2,3]triazol-4-ylmethyl]-amino}-pyrimidin-4-ylamino)-piperidin-1-yl]-ethyl}-phosphonic Acid Hydrobromide Salt (CN024)

Starting from 2,4,6-trichloropyrimidine (250 mg, 1.36 mmol), CN024 (114 mg) was obtained in 11% yield over six steps. ^1^H NMR (400 MHz, CD_3_OD): δ 8.00 (s, 1H), 4.71 (s, 2H), 4.52 (t, *J* = 7.2 Hz, 2H), 4.24 (m, 4H), 4.11 (m, 1H), 3.74 (m, 2H), 3.57 (t, *J* = 6.0 Hz, 2H), 3.43 (m, 2H), 3.33 (m, 2H), 3.18 (m, 4H), 2.34–2.24 (m, 4H), 2.12 (m, 2H), 1.91 (m, 2H). ^13^C NMR (150 MHz, D_2_O): δ 161.2, 153.5, 152.3, 145.2, 123.6, 72.5, 58.1, 52.6, 51.3, 50.7, 47.3, 46.2, 43.1, 36.2, 31.8, 28.8, 23.3 (d, *J* = 129.6 Hz). ESMS *m*/*z*: 574.2 [M + H]^+^. HRMS (ESI) *m*/*z*: calcd for C_21_H_37_N_9_O_6_PS [M + H]^+^, 574.2325; found, 574.2331. HPLC purity = 95.3%, *t*_R_ = 7.41 min.

#### {2-[4-(2-{[1-(2-Hydroxy-propyl)-1*H*-[1,2,3]triazol-4-ylmethyl]-amino}-6-morpholin-4-yl-pyrimidin-4-ylamino)-piperidin-1-yl]-ethyl}-phosphonic Acid Hydrobromide Salt (CN025)

Starting from 2,4,6-trichloropyrimidine (250 mg, 1.36 mmol), CN025 (151 mg) was obtained in 16% yield over six steps. ^1^H NMR (400 MHz, CD_3_OD): δ 8.44 (s, 1H), 4.85 (s, 2H), 4.64 (dd, *J* = 13.6, 3.6 Hz, 1H), 4.42 (dd, *J* = 13.6, 8.0 Hz, 1H), 4.25–4.13 (m, 2H), 3.80–3.64 (m, 10H), 3.42–3.36 (m, 4H), 2.40–2.29 (m, 4H), 1.96 (m, 2H), 1.24 (d, *J* = 4.4 Hz, 3H). ^13^C NMR (150 MHz, D_2_O): δ 161.8, 154.7, 153.6, 144.6, 124.9, 72.3, 66.1, 57.0, 52.1, 51.4, 48.9, 46.0, 44.9, 35.8, 28.7, 23.0 (d, *J* = 131.6 Hz), 19.1. ESMS *m*/*z*: 526.2 [M + H]^+^. HRMS (ESI) *m*/*z*: calcd for C_21_H_37_N_9_O_5_P [M + H]^+^, 526.2655; found, 526.2656. HPLC purity = 95.5%, *t*_R_ = 8.62 min.

#### {2-[4-(2-{[1-(3-Hydroxy-butyl)-1*H*-[1,2,3]triazol-4-ylmethyl]-amino}-6-morpholin-4-yl-pyrimidin-4-ylamino)-piperidin-1-yl]-ethyl}-phosphonic Acid Hydrobromide Salt (CN026)

Starting from 2,4,6-trichloropyrimidine (250 mg, 1.36 mmol), CN026 (159 mg) was obtained in 17% yield over six steps. ^1^H NMR (400 MHz, CD_3_OD): δ 8.17 (s, 1H), 4.75 (s, 2H), 4.58 (t, *J* = 6.8 Hz, 2H), 4.10 (m, 1H), 3.80–3.70 (m, 10H), 3.47–3.43 (m, 3H), 3.31 (m, 2H), 2.36–2.25 (m, 4H), 2.08 (m, 2H), 1.96 (m, 2H), 1.22 (d, *J* = 6.4 Hz, 3H). ^13^C NMR (150 MHz, D_2_O): δ 161.7, 153.3, 152.1, 145.2, 123.5, 72.5, 66.0, 64.6, 52.5, 51.4, 47.5, 46.0, 44.9, 38.0, 36.0, 28.7, 23.2 (d, *J* = 129.8 Hz), 22.0. ESMS *m*/*z*: 540.2 [M + H]^+^. HRMS (ESI) *m*/*z*: calcd for C_22_H_39_N_9_O_5_P [M + H]^+^, 540.2812; found, 540.2815. HPLC purity = 97.4%, *t*_R_ = 8.89 min.

#### {2-[4-(2-{[1-(3-Hydroxy-3-methyl-butyl)-1*H*-[1,2,3]triazol-4-ylmethyl]-amino}-6-morpholin-4-yl-pyrimidin-4-ylamino)-piperidin-1-yl]-ethyl}-phosphonic Acid Hydrobromide Salt (CN027)

Starting from 2,4,6-trichloropyrimidine (250 mg, 1.36 mmol), CN027 (172 mg) was obtained in 18% yield over six steps. ^1^H NMR (400 MHz, CD_3_OD): δ 8.12 (s, 1H), 4.73 (s, 2H), 4.57 (dd, *J* = 9.6, 6.8 Hz, 2H), 4.11 (m, 1H), 3.80–3.64 (m, 10H), 3.44 (m, 2H), 3.31 (m, 2H), 2.33–2.22 (m, 4H), 2.08 (m, 2H), 1.90 (m, 2H). 1.27 (s, 6H). ^13^C NMR (150 MHz, D_2_O): δ 160.6, 152.4, 150.7, 143.8, 123.7, 72.1, 70.0, 65.9, 52.6, 51.4, 50.5, 48.9, 46.7, 42.4, 36.0, 28.7, 27.6, 23.2 (d, *J* = 132.4 Hz). ESMS *m*/*z*: 554.3 [M + H]^+^. HRMS (ESI) *m*/*z*: calcd for C_23_H_40_N_9_Na_1_O_5_P [M + Na]^+^, 576.2788; found, 576.2785. HPLC purity = 96.6%, *t*_R_ = 8.68 min.

#### {2-[4-(2-{[1-(3-Methoxy-propyl)-1*H*-[1,2,3]triazol-4-ylmethyl]-amino}-6-morpholin-4-yl-pyrimidin-4-ylamino)-piperidin-1-yl]-ethyl}-phosphonic Acid Hydrobromide Salt (CN028)

Starting from 2,4,6-trichloropyrimidine (250 mg, 1.36 mmol), CN028 (163 mg) was obtained in 17% yield over six steps. ^1^H NMR (600 MHz, D_2_O): δ 8.06 (s, 1H), 4.75 (s, 2H), 4.53 (t, *J* = 7.2 Hz, 2H), 3.92 (m, 1H), 3.75–3.50 (m, 10H), 3.39 (m, 2H), 3.29 (t, *J* = 6.0 Hz, 2H), 3.27 (s, 3H), 3.17 (m, 2H), 2.30 (m, 2H), 2.23–2.08 (m, 4H), 1.79 (m, 2H). ^13^C NMR (150 MHz, D_2_O): δ 161.5, 153.3, 152.0, 142.9, 125.1, 71.1, 68.7, 66.0, 58.2, 51.7, 51.3, 48.5, 46.2, 45.0, 33.1, 31.3, 26.6, 23.1 (d, *J* = 130.2 Hz). ESMS *m*/*z*: 540.2 [M + H]^+^. HRMS (ESI) *m*/*z*: calcd for C_22_H_39_N_9_O_5_P [M + H]^+^, 540.2812; found, 540.2813. HPLC purity = 95.4%, *t*_R_ = 8.93 min.

### Biology

All experimental design of behavioral studies and animal care were accredited by the institutional animal ethics committee of the National Cheng Kung University (approved IACUC number: 106280) and followed with Animal Care Guidelines. In addition, all cell culture experiments were designed and performed as follows.

### Image Acquisition and Analysis on the Image-Based HCS

Primary cortical or DRG cells were fixed with 4% paraformaldehyde for 15 min following compound and paclitaxel treatment. After washing with PBS, the fixed cells were permeabilized with PBS containing 0.05% Triton X-100 for 20 min and then blocked with commercial blocking buffer (#GTX30963; GeneTex, USA) at room temperature for 1 h. Subsequently, the cortical cells were stained for the extending processes and synaptic puncta by using anti-MAP2 (#MBS502140, 1:500; Mybiosource, USA) and anti-synaptophysin (#ab32127, 1:500; Abcam, UK) overnight. DRG cells were stained for the neurite and neuron nucleus by using anti-β III tubulin (#D71G9, 1:600; Cell Signaling, USA) and anti-NeuN monoclonal antibody (#MAB377, 1:400; Millipore, USA) overnight. On the second day, the samples were washed for 30 min with wash buffer and incubated with secondary antibodies (Alexa-488, 647 1:600; Thermo Fisher, USA) and 4 μg/mL Hoechst 33258 (#94403-1ML, 1:100; Merck, USA) at room temperature for 1 h. In the initial experiments of screening the potential neuroprotective compound for CIPN, ImageXpress^Micro^ (IXM) high-content imaging system (Molecular Devices, USA) driven by MetaXpress software was used. Images of treated cells were automatically acquired by using a 10× objective. Twenty-one images per well were taken in each of three channels (DAPI, FITC, and Cy5) to create a whole view of the entire neural network. The integrity of the neural network was evaluated using TIF images by importing them into MetaXpress software for analysis using the module for multiparameter, such as Neurite Outgrowth, Cell Scoring, and Transfluor. Furthermore, the score of synaptogenesis was analyzed in AcuityXpress software (Molecular Devices, USA).

### Animal Behavioral Tests and Drug Administration for Paclitaxel-Induced Neuropathy

The baseline of every neurological behavior test was measured in 6-week-old C57BL/6J female mice prior to treatment. In the first week, 7-week-old C57BL/6J female mice did not undergo any tests, and CN016 (5, 10 and 20 mg/kg) was given by IP 1 h before IP of paclitaxel (4.5 mg/kg) on alternate days (days 1, 3, 5, and 7). Three behavioral tests were performed on the same groups of animals once per week and no more than two behavioral tests on the same day. The first behavioral experiment was performed on the next day after the last course of treatment. Mechanical hyperalgesia was evaluated by using von Frey filament (#2390, IITC Inc., USA), thermal sensitivity was determined by the tail immersion assay (water temperature: 48–49 °C), and motor coordination was assessed by rotarod (#7750, Ugo Basile Biological Research Apparatus, Italy). After the first drug implantation, the body weight was recorded every 6 days throughout the entire experiment.

### Hemodynamic Monitoring

Noninvasive automatized tail-cuff system (Visitech Systems, Apex, NC, USA) was used to measure the systolic pressure and the heart rate of conscious mice. All mice were placed in a constant temperature platform at least three times to adapt the system prior to hemodynamic measurement. The root of the tail was mounted on the occlusion and sensor cuff after the mouse was placed in a constant temperature platform. Each mouse was measured for at least 15 repetitions to obtain the mean of systolic pressure and heart rate. The hemodynamic change of each mouse was measured at five different time points.

### Systemic Mouse Cytokine and Chemokine Detection

Samples were obtained from C57BL/6J female mice before and 14 days after the first course of treatment. The blood samples were obtained from the submandibular vein and then centrifuged at 3000 rpm for 15 min at 4 °C to yield plasma. Mouse cytokines/chemokines were quantified using Magnetic Bead 32-Multiplex Panel (Millipore, MCYTMAG-70K-PX32). Statistical significance was analyzed using two-way ANOVA, with *p* < 0.05 considered significant.

### Ultrastructure Evaluation of the Sciatic Nerve

Sciatic nerve samples from mice from each treatment group were isolated after the last time point of mouse behavioral tests. Fixation of sciatic nerves with 4% glutaraldehyde and post fixation with 1% osmium tetroxide solution at 4 °C were performed. These samples were dehydrated in graded series of alcohol, embedded in EMbed 812 (EMS; #14120), and sliced to a thickness to 90 nm. Transmission electron microscopy (H7650, Hitachi) was used to observe the ultrastructure in the cross section of the sciatic nerves.

### Immunofluorescence for Image Analyses

DRG tissues were isolated on day 7 after first paclitaxel injection. The tissues were fixed in 4% paraformaldehyde at 4 °C overnight and subsequently placed in 30% sucrose for sample dehydration. During the frozen section procedure, the tissues were embedded in OCT (EMS; #14120) and sectioned into 20 μm thick slices. The DRG tissue sections were permeabilized with 0.1% Triton X-100 in PBS for 1 h and then blocked with CAS-Block solution (#008120; Thermo Fisher, USA) at room temperature for another 1 h. DRG tissue sections were stained with primary antibody against CD68 (1:100 dilution; Novus), NOS2 (1:100 dilution; Santa Cruz), Arginase-1 (1:100 dilution; Santa Cruz), and Iba-1 (1:100 dilution; Santa Cruz) at 4 °C overnight. Afterward, the samples were washed with PBS and stained with secondary antibodies (Alexa-488, 594, 1:200 dilution; Invitrogen) and Hoechst 33342 (4 μg/mL; Invitrogen) at room temperature for 1 h. A 60× objective by an Olympus FV3000 confocal microscope was used to acquire immunofluorescence images.

### Toxicology

An acute single-dose toxicity study was conducted in male ICR mice. 7-week-old ICR male mice were intravenous bolus administered with CN016 dissolved in 0.9% sodium chloride solution by using dose escalation procedure to approach the toxicity of CN016. The treatment groups included 50, 100, 300, and 500 mg/kg dose levels and three to six mice for each group. The injection volume was calculated based on the individual body weight. All animals were observed for clinical signs twice daily after the first day of drug treatment. Animal body weight was measured once daily during the 14 day observation period of the study. All animals were euthanized with 100% CO_2_ at the end of the study.

### Pharmacokinetics

Male ICR mice weighed (29–33 g) were obtained from BioLASCO (Taiwan Co., Ltd., Ilan, Taiwan). The animal studies were performed according to NHRI institutional animal care and committee-approved procedures. Food and water were available ad libitum throughout the experiment. A single 5.0 mg/kg dose of CN016 was dissolved in normal saline and was separately administered to mice intravenously. The groups consisted of three mice for each time point. At times 0.03, 0.08, 0.25, 0.5, 1, 2, 4, 6, 8, 16, and 24 h after dosing, blood was collected from groups of three mice at each time point by cardiac puncture. Plasma was separated from blood and stored in a freezer (−80 °C) before LC/MS/MS analysis. In addition, pharmacokinetic analysis of CN016 following intraperitoneal administration (IP, 5 mg/kg) is provided in Figure S2 (Supporting Information).

### Statistical Analysis

Data are expressed as mean ± S.E.M. Quantifications in morphology-based screening data were analyzed with the two-tailed Student’s *t*-test. Mice were randomly divided into eight groups for the behavior test. For comparing response differences, data were analyzed by two-way ANOVA. The significance criterion in statistics was *p* < 0.05.

## Supporting Information Available

+Molecular formula strings of all tested compounds (CSV)
Synthetic procedures of CN001–CN003, CN005–CN011, CN013–CN015, and CN017–CN019 and copies of HPLC traces as well as ^1^H and ^13^C NMR spectra of compounds CN004, CN012, CN016, and CN020–CN028 (PDF)

## Abbreviations

CIPN: chemotherapy-induced peripheral neuropathy
CXCR4: G protein-coupled CXC chemokine receptor 4
DRG: dorsal root ganglion
ESMS: electrospray mass spectra
HCS: high-content screening
MTD: maximum tolerance dose
MED: minimum efficacy dose
PIPN: paclitaxel-induced peripheral neuropathy
SAR: structure–activity relationships
SC: subcutaneous
SD: standard deviation
SEM: standard error of the mean
