# Supplementary material for: Discovery of Potential Neuroprotective Agents against Paclitaxel-Induced Peripheral Neuropathy
Source: J Med Chem. 2022 Mar 2;65(6):4767–82. doi: 10.1021/acs.jmedchem.1c01912 (PMC8958505; doi:10.1021/acs.jmedchem.1c01912)
Supplement: Supplementary file 2 — jm1c01912_si_002.pdf [file jm1c01912_si_002.pdf]

Supporting Information

Discovery of Potential Neuroprotective Agents against Paclitaxel-induced Peripheral Neuropathy

Yi-Fan Chen,[2,4] Chien-Huang Wu,[1] Li-Hsien Chen,[2] Hao-Wei Lee,[1] Jinq-Chyi Lee,[1] Teng-Kuang Yeh,[1] Jang-Yang Chang,[1,*] Ming-Chen Chou,[1] Hui-Ling Wu,[1] Yen-Po Lai,[1] Jen-Shin Song,[1] Kai-Chia Yeh,[1] Chiung-Tong Chen,[1] Chia-Jui Lee,[1] Kak-Shan Shia,[1,*] and Meng-Ru Shen[2,3,*]

[1]Institute of Biotechnology and Pharmaceutical Research, National Health Research Institutes, Miaoli County 35053, Taiwan, R.O.C.

[2]Department of Pharmacology, College of Medicine, National Cheng Kung University, Tainan City 70101, Taiwan, R.O.C

[3]Department of Obstetrics & Gynecology, National Cheng Kung University Hospital, College of Medicine, National Cheng Kung University, Tainan City 70101, Taiwan, R.O.C.

[4]Institute of Basic Medical Sciences, College of Medicine, National Cheng Kung University, Tainan City 70101, Taiwan, R.O.C

Contents

1). Table S1. The synaptogenesis and neurite outgrowth assays of 78 minoxidil-related derivatives--- S1-S8

2). Table S2: The neurite outgrowth assay of CN001~CN019----------------- S9

3). Table S3: hERG patch-clamp assay of CN016 ------------------------------- S10

4). Table S4: Cytochrome P450 testing of CN016------------------------------- S11

5). Table S5: 67 off-target study of CN016--------------------------------------- S12-S19

6). Figure S1: The safety assessment of CN016 including rotarod, body weight, and hemodynamic monitoring tests---------------------------------- S20

7). Figure S2: The pharmacokinetics of CN016 by IP administration--------- S21

8). Synthesis of CN001~CN003, CN005~CN011, CN013~CN015, CN017~CN019, CN020, CN021, and CN025------------------------------- S22-S48

9). ^{1}H and ^{13}C NMR spectra of CN004, CN012, CN016, and CN020~CN028--- S49-S72

10). HPLC analyses of CN004, CN012, CN016, and CN020~CN028--------- S73-S84

Table S1. The Synaptogenesis and Neurite Outgrowth Assay of 78 minoxidil-related derivatives

Test Compounds	Structure (SMILES)	A[a]	B[b]	C[c]	D[d]
CSV0A062712	O=C(C1=CC=CC=C1C)NNC2=NC(N3CCOCC3)=NC(N4CCOCC4)=C2	52±7	51±2	67±10	63±6
CSV0A024811	O=[N+](C1=C(OCO2)C2=CC(/C=N/NC3=NC(N4CCCCCC4)=NC(N5CCCCCC5)=C3)=C1)[O-]	52±1	46±4	69±7	60±3
CSV0A027457	ClC(C=C1)=CC=C1/C=N/NC2=NC(N3CCCCCC3)=NC(N4CCCCCC4)=C2	51±4	39±4	62±4	51±4
CSV0A013031	COC(C=CC=C1)=C1/C=N/NC2=NC(N3CCOCC3)=NC(N4CCOCC4)=C2	57±1	33±3	74±3	67±6
CSV0A024810	C1(N/N=C/C2=CC=NC=C2)=NC(N3CCCCC3)=NC(N4CCCCC4)=C1	38±3	40±4	68±1	67±1
CSV0A027460	CN(C)C(C=C1)=CC=C1/C=N/NC2=NC(N3CCCC3)=NC(N4CCCC4)=C2	47±1	33±3	77±1	63±3
CSV0A013030	BrC1=CC(/C=N/NC2=NC(N3CCOCC3)=NC(N4CCOCC4)=C2)=C(O)C=C1	26±2	26±3	76±5	89±11
CSV0A047584	CN(C)C1=NC(N/N=C/C2=CC=CC=C2O)=CC(N(C)C)=N1	22±2	24±4	88±7	78±11
CSV0A055770	OC1=CC(/C=N/NC2=NC(N3CCCC3)=NC(N4CCCC4)=C2)=CC=C1	24±2	25±2	82±4	75±4
CSV0A024809	C1(N/N=C/C2=CC=NC=C2)=NC(N3CCOCC3)=NC(N4CCOCC4)=C1	38±3	27±0.5	77±2	82±3
CSV0A041617	CN(C)C(C=C1)=CC=C1/C=N/NC2=NC(N3CCOCC3)=NC(N4CCOCC4)=C2	30±1	30±1	93±5	94±1

CSV0A024808	ClC(C=C1)=CC=C1/C=N/NC2=NC(N3CCOCC3)=NC(N4CCOCC4)=C2	27±1	25±1	91±1	89±4
CSVOD047961	CCOC(C=C1)=CC=C1NC2=NC(NCC3=CC=CC=C3)=C([N+]([O-])=O)C(/N=C/C4=C(C=CC=C5)C5=CC(C(NC6=CC=CC=C6C)=O)=C4OC)=N2	30±3	36±1	90±2	95±2
CSV0A055768	CN(C)C1=NC(N/N=C/C2=CC=C(O)C(OC)=C2)=CC(N(C)C)=N1	35±4	33±0.2	71±2	74±6
CSV0A062711	O=C(CCC1=CC=CC=C1)NNC2=NC(N3CCCCC3)=NC(N4CCCCC4)=C2	36±0.5	28±3	80±11	79±6
CSV0A027459	CN(C)C1=NC(N/N=C/C2=CC=C(Cl)C=C2)=CC(N(C)C)=N1	35±1	37±2	76±7	79±11
CSV0A062713	O=C(C1=CC=C(C(C)(C)C)C=C1)NNC2=NC(N3CCCC3)=NC(N4CCCC4)=C2	33±1	36±2	85±8	74±5
CSV0A055771	COC(C=CC=C1)=C1/C=N/NC2=NC(N3CCCC3)=NC(N4CCCC4)=C2	36±2	40±8	68±6	68±3
CSV0A024814	ClC(C=C1)=CC=C1/C=N/NC2=NC(N3CCCC3)=NC(N4CCCC4)=C2	46±1	31±1	81±0.005	67±2
CSV0A024812	O=[N+](C(C=C1)=CC=C1/C=N/NC2=NC(N3CCCC3)=NC(N4CCCC4)=C2)[O-]	55±6	62±3	53±3	60±1
CSV0A034003	CN(C)C1=NC(N/N=C/C2=CC=CC=C2O)=CC(N(C)C)=N1	67±5	66±9	61±3	62±3
CSV0A041619	OC(C=CC(Cl)=C1)=C1/C=N/NC2=NC(N3CCCC3)=NC(N4CCCC4)=C2	54±0.03	62±2	62±2	61±2
CSV0A024806	O=[N+](C(C=C1)=CC=C1	68±6	78±5	70±3	68±6

	/C=N/NC2=NC(N3CCOCC3)=NC(N4CCOCC4)=C2)[O-]				
CSV0A047585	CN(C)C1=NC(N/N=C/C2=CC=C(Cl)C=C2Cl)=CC(N(C)C)=N1	67±2	56±0.2	57±2	57±2
CSV0A0544S0	NC1=NC(NC2=C([S-])C=CC(C)=[NH+]2)=CC(N)=N1	63±5	52±8	60±2	57±5
CSV0A024804	CCN(CC)C(C=C1)=CC=C1/C=N/NC2=NC(N3CCOCC3)=NC(N4CCOCC4)=C2	67±8	62±3	68±2	66±1
CSV0A047582	CN(C)C1=NC(N/N=C/C2=CC=CC=C2)=CC(N(C)C)=N1	73±3	77±9	69±3	67±3
CSV0A051549	O=C(C1=CC=CC=C1)NNC2=NC(N3CCOCC3)=NC(N4CCOCC4)=C2	86±6	77±7	71±1	68±1
CSV0A027458	CN(C)C1=NC(N/N=C/C2=CC=CC([N+]([O-])=O)=C2)=CC(N(C)C)=N1	73±3	73±3	71±3	65±3
CSV0A047583	CN(C)C1=NC(N/N=C/C2=CC3=C(OCO3)C([N+]([O-])=O)=C2)=CC(N(C)C)=N1	66±4	69±5	63±3	61±3
CSV0A024807	O=[N+](C1=CC(/C=N/NC2=NC(N3CCOCC3)=NC(N4CCOCC4)=C2)=CC=C1)[O-]	92±2	97±2	69±3	60±1
CSV0A024813	COC(C=C1)=CC=C1/C=N/NC2=NC(N3CCCC3)=NC(N4CCCC4)=C2	98±4	81±5	66±2	63±2
CSV0A013029	OC(C=C1)=CC=C1/C=N/NC2=NC(N3CCOCC3)=NC(N4CCOCC4)=C2	72±2	84±6	58±2	54±5
CSV0A024085	OC(C=CC=C1)=C1/C=N/NC2=NC(N3CCOCC3)=N	71±4	68±5	64±3	53±2

	C(N4CCOCC4)=C2				
CSV0A055769	CN(C)C1=NC(N/N=C/C2=CC=CS2)=CC(N(C)C)=N1	83±1	97±8	68±3	62±2
CSV0A041618	CN(C)C(C=C1)=CC=C1/C=N/NC2=NC(N3CCCCC3)=NC(N4CCCCC4)=C2	83±2	64±7	55±2	55±2
CSV0A034004	CN(C)C1=NC(N/N=C/C2=CC=C(OC)C=C2)=CC(N(C)C)=N1	65±6	76±2	54±7	55±4
CSV0A020149	ClC(C=C1)=CC=C1/C=N/NC2=NC(N(CC)CC)=NC(N(CC)CC)=C2	43±1	36±1	52±2	48±3
CSV0A023089	CN(C)C1=NC(N(C)C)=CC(NNC(C2=CC=C(Cl)C=C2)=O)=N1	38±3	39±6	47±2	50±5
CSV0A007879	CCN(CC)C1=NC(N(CC)CC)=CC(N/N=C/C2=CC=CC(O)=C2)=N1	38±3	26±5	42±5	35±4
CSV0A011999	COC1=CC=CC=C1/C=N/NC2=CC(N(CCC)CCC)=NC(N(CCC)CCC)=N2	33±4	37±2	45±5	53±3
CSV0A061665	ClC(C=C1)=CC=C1/C=N/NC2=NC(N(CCC)CCC)=NC(N(CCC)CCC)=C2	27±3	27±5	37±7	27±3
CSV0A012003	CCN(CC)C1=NC(N(CC)CC)=CC(N/N=C/C2=C(O)C=CC(Br)=C2)=N1	27±3	31±3	34±3	36±7
CSV0A020330	BrC1=CC(/C=N/NC2=NC(N(CCC)CCC)=NC(N(CCC)CCC)=C2)=C(O)C=C1	22±3	45±7	27±2	45±3
CSV0A011998	O=[N+](C(C=C1)=CC=C1/C=N/NC2=NC(N3CCCCC3)=NC(N4CCCCC4)=C2)[O-]	75±1	55±2	77±5	61±6
CSV0A007655	CCN(CC)C1=NC(N/N=C/C2=CC=CC=C2OC)=CC(N(CC)CC)=N1	39±4	41±2	45±7	51±3

CSV0A021190	CN(C)C1=NC(N(C)C)=CC(N/N=C/C2=CC=CC(O)=C2)=N1	37±1	45±1	43±1	50±2
CSV0A012753	O=[N+](C(C=C1)=CC=C1/C=N/NC2=NC(N3CCCCCC3)=NC(N4CCCCCC4)=C2)[O-]	60±1	61±4	77±7	75±7
CSV0A023090	CCN(CC)C(C=C1)=CC=C1/C=N/NC2=NC(N3CCCCC3)=NC(N4CCCCC4)=C2	82±4	86±0.4	57±4	58±3
CSV0A2327S0	CN(C)C1=NC(NNC(C2=CC=CC=C2O)=O)=CC(N(C)C)=N1	42±2	41±5	55±1	53±5
CSV0A008237	CCN(CC)C1=NC(NNC(C2=CC=CC=C2OC)=O)=CC(N(CC)CC)=N1	45±4	45±8	56±1	50±9
CSV0A029945	CCN(CC)C1=NC(N(CC)CC)=CC(N/N=C/C2=CC=NC=C2)=N1	35±6	50±0.3	41±8	75±8
CSV0A005710	C1(N/N=C/C2=CC=NC=C2)=NC(N3CCCC3)=NC(N4CCCC4)=C1	35±2	41±6	82±7	86±12
CSV0A061667	C1(N/N=C/C2=CC=NC=C2)=NC(N3CCCCCC3)=NC(N4CCCCCC4)=C1	40±6	41±7	82±6	82±6
CSV0A005154	BrC1=CC(/C=N/NC2=NC(N3CCCCC3)=NC(N4CCCCC4)=C2)=C(O)C=C1	38±4	30±4	82±7	72±7
CSV0A023088	CCN(CC)C1=NC(N(CC)CC)=CC(N/N=C/C2=C(O)C=CC(Br)=C2)=N1	33±3	40±5	43±4	47±2
CSV0A061666	COC1=CC=CC=C1/C=N/NC2=CC(N3CCCCC3)=NC(N4CCCCC4)=N2	41±1	42±4	91±3	89±7
CSV0B001120	OC1=CC(/C=N/NC2=NC(N3CCCCC3)=NC(N4CCCCC4)=C2)=CC=C1	43±5	41±5	95±9	82±2

CSV0B002167	C1(N/N=C/C2=CC=NC=C2)=NC(N3CCCC3)=NC(N4CCCC4)=C1	37±7	30±3	82±6	68±7
CSV0B002003	BrC1=CC(/C=N/NC2=NC(N3CCCCC3)=NC(N4CCCCC4)=C2)=C(O)C=C1	33±1	40±0.2	83±6	86±3
CSV0B002732	C1(N/N=C/C2=CC=NC=C2)=NC(N3CCCCC3)=NC(N4CCCCC4)=C1	41±3	42±3	84±6	88±1
CSV0B001160	O=C(C1=CC=C(C(C)(C)C)C=C1)NNC2=NC(N3CCCCC3)=NC(N4CCCCC4)=C2	51±5	41±1	89±7	80±5
CSV0B026196	O=C(C1=CC=C(C(C)(C)C)C=C1)NNC2=NC(N3CCOCC3)=NC(N4CCOCC4)=C2	36±2	32±1	83±5	65±4
CSV0B002001	CN(C)C1=NC(N(C)C)=CC(NNC(C2=CC=C(C(C)(C)C)C=C2)=O)=N1	35±3	35±3	44±3	44±3
CSV0B008884	CCN(CC)C1=NC(N(CC)CC)=CC(NNC(C2=CC=C(C(C)(C)C)C=C2)=O)=N1	35±3	37±3	41±3	41±4
CSV0B016174	OC1=CC=C(C=C1OC)/C=N/NC2=CC(N3CCCC3)=NC(N4CCCC4)=N2	43±3	36±4	86±3	76±4
CSV0B002704	OC1=CC=C(C=C1OC)/C=N/NC2=CC(N3CCOCC3)=NC(N4CCOCC4)=N2	38±2	29±6	79±7	65±3
CSV0B006088	OC1=CC=C(C=C1OC)/C=N/NC2=CC(N3CCCCC3)=NC(N4CCCCC4)=N2	45±6	52±10	59±5	66±7
CSV0B009489	OC1=CC=C(C=C1OC)/C=N/NC2=CC(N(CC)CC)=NC(N(CC)CC)=N2	34±3	36±3	39±4	41±3
CSV0B023377	OC(C=CC=C1)=C1/C=N/NC2=NC(N3CCCCC3)=NC(N4CCCCC4)=C2	42±1	42±3	57±2	57±4

CSV0B002764	OC(C=CC=C1)=C1/C=N/NC2=NC(N3CCOCC3)=NC(N4CCOCC4)=C2	48±5	47±3	66±6	67±4
CSV0B009305	CN(C)C1=NC(N(C)C)=CC(N/N=C/C2=C(O)C=CC=C2)=N1	36±3	38±3	39±2	40±2
CSV0B018834	CCN(CC)C1=NC(N(CC)CC)=CC(N/N=C/C2=C(O)C=CC=C2)=N1	30±3	31±3	39±3	39±2
CSV0B009488	CCN(CC)C1=NC(N(CC)CC)=CC(NNC(CCC2=CC=CC=C2)=O)=N1	38±3	34±3	35±4	44±3
CSV0B008273	CN(C)C1=NC(N(C)C)=CC(NNC(CCC2=CC=CC=C2)=O)=N1	39±2	36±2	46±3	53±2
CSV0C019163	O=C(CCC1=CC=CC=C1)NNC2=NC(N3CCCC3)=NC(N4CCCC4)=C2	49±4	53±1	68±7	74±1
CSV0C009114	O=C(CCC1=CC=CC=C1)NNC2=NC(N3CCOCC3)=NC(N4CCOCC4)=C2	48±5	49±3	70±7	65±3
CSV0D043578	O=C(CCC1=CC=CC=C1)NNC2=NC(N(CCC)CCC)=NC(N(CCC)CCC)=C2	38±3	42±4	42±5	48±1
Minoxidil		NA	58±8	NA	66±3

[a]A: Synaptogenesis (% of control) of test compound (10 nM) + paclitaxel (1 μM), values represent the mean ± SE of at least three independent experiments.

[b]B: Synaptogenesis (% of control) of test compound (1000 nM) + paclitaxel (1 μM)

[c]C: Neurite outgrowth (% of control) of test compound (10 nM) + paclitaxel (1 μM)

[d]D: Nneurite outgrowth (% of control) of test compound (1000 nM) + paclitaxel (1 μM)

Note: Both synaptogenesis and neurite outgrowth are important for central nervous system development (Neural Dev. 2021 Jan 7;16(1):1). Therefore, only compounds displaying a better neuroprotective effect on both neurite outgrowth and synaptogenesis assays than the positive control (Minoxidil) are elected as potential hits, highlighted in red in Table S1, for further development, whose structures are also outlined below.

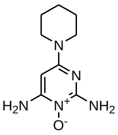

Minoxidil

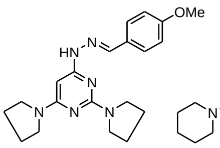

CSV0A024813

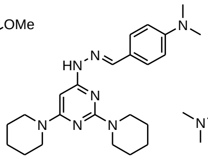

CSV0A041618

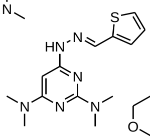

CSV0A055769

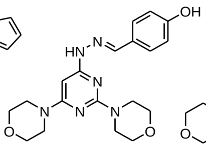

CSV0A013029

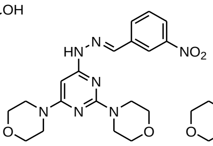

CSV0A024807

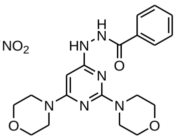

CSV0A051549

Table S2. The neurite outgrowth assay of compounds CN001~CN019 in Figure 2

Cmpd	Neuroprotection rate[a] = (A-B) ± S.E.M [%]
**CN001**	-6 ± 0.30
**CN002**	-4 ± 0.81
**CN003**	1 ± 2.60
**CN004**	1 ± 3.20
**CN005**	-3 ± 0.41
**CN006**	4 ± 1.44
**CN007**	7 ± 1.05
**CN008**	4 ± 1.44
**CN009**	15 ± 1.28
**CN010**	5 ± 3.06
**CN011**	4 ± 4.10
**CN012**	9 ± 1.00
**CN013**	6 ± 1.17
**CN014**	9 ± 2.00
**CN015**	3 ± 1.11
**CN016**	21 ± 0.77
**CN017**	1 ± 0.51
**CN018**	3 ± 2.20
**CN019**	-2 ± 1.20

[a]A: Neurite outgrowth (% of control) of test compound (1000 nM) + paclitaxel (50 nM); B: Neurite outgrowth (% of control) of paclitaxel (50 nM).

hERG patch-clamp assay

CN016 were tested in the *hERG* (*hERG*-CHO, automated patch-clamp) assay and determined at Eurofins Panlabs (Missouri, USA). The experiment was accepted in accordance with Eurofins validation Standard Operating Procedure. The tested result was listed in Table S3.

Table S3. Result of *hERG* patch-clamp assay of CN016

Tested [C]	100 μM	10 μM	1 μM
Inhibition	32.9%	19.1%	6.0%

Cytochrome P450 testing of CN016

The effects of CN016 on the activity of six specific human liver cytochrome P450 isozymes (CYP 1A2, 2C9, 2C19, 2D6, 2E1, 3A4) expressed in baculovirus-infected insect cell supersomes were studied. The reactions used to measure the activity of these enzymes were phenacetin O-deethylation (1A2), diclofenac 4-hydroxylation (2C9), mephenytoin 4-hydroxylation (2C19), dextromethorphan O-demethylation (2D6), chlorzoxazone 6-hydroxylation (2E1) and testosterone 6-hydroxylation (3A4). Furafylline, sulfaphenazole, tranylcypromine, quinidine, diethyldithiocarbamate and azamulin were used as reference inhibitors of these enzymes. Incubation mixtures containing no CN016 or reference inhibitors were used as controls. The tested result was listed in Table S4.

Table S4. Result of Cytochrome P450 testing of CN016

CYP450	1A2	2C9	2C19	2D6	2E1	3A4
Concentration of inhibition	>100 μM	>100 μM	>100 μM	>100 μM	>100 μM	>100 μM

Table S5. 67 off-target study of CN016 at 10 μM

Tested item	Inhibition (%)	Tested item	Inhibition (%)
Adenosine A_1	-9	Histamine H_3	2
Adenosine A_{2A}	5	Imidazoline I_2, Central	-5
Adenosine A_3	0	Interleukin IL-1 R1	-3
Adrenergic α_{1A}	-9	Leukotriene, Cysteinyl CysL T_1	-8
Adrenergic α_{1B}	14	Melatonin MT_1	6
Adrenergic α_{1D}	-6	Muscarinic M_1	5
5drenergic α_{2A}	-4	Muscarinic M_2	-1
A-1energic β_1	4	Muscarinic M_3	0
Adr-nergic β_2	-4	Neuropeptide Y Y_1	-4
Androgen (Testosterone)	6	Neuropeptide Y Y_2	10
Bradykinin B_1	-10	Nicotinic Acetylcholine	-6
Bradykinin B_2	-4	Nicotinic Acetylcholine α1, Bungarotoxin	7
Calcium Channel L-Type, Benzothiazepine	-13	Opiate δ_1 (OP1, DOP)	6
Calcium Channel L-Type, Dihydropyridine	14	Opiate κ(OP2, KOP)	3
Calcium Channel N-Type	1	Opiate μ(OP3, MOP)	1
Cannabinoid CB_1	-5	Phorbol Ester	-3
Dopamine D1	-2	Platelet Activating Factor (PAF)	-6
Dopamine D_{2S}	3	Potassium Channel [K_{ATP}]	-1
Dopamine D_3	-3	Potassium Channel hERG	-3
Dopamine $D_{4.4}$	12	Prostanoid EP_4	4
Endothelin ETA	15	Purinergic P2X	-9
Endothelin ETB	-1	Purinergic P2Y	9
Epidermal Growth Factor (EGF)	-1	Rolipram	6
Estrogen ERα	-17	Serotonin (5-Hydroxytryptamine) 5-HT1A	2
GABAA, Flunitrazepam, Central	-5	Serotonin (5-Hydroxytryptamine) 5-HT2B	10
GABAA, Muscimol, Central	5	Serotonin (5-Hydroxytryptamine) 5-HT3	-1
$GABA_{B1A}$	14	Sigma σ_1	0
Glucocorticoid	14	Sodium Channel, Site 2	-1
Glutamate, Kainate	0	Thyroid Hormone	7
Glutamate, NMDA, Agonism	-3	Transporter, Dopamine (DAT)	0
Glutamate, NMDA, Glycine	-5	Transporter, GABA	2
Glutamate,NMDA, Phencyclidine	-4	Transporter, Norepinephrine (NET)	-1
Histamine H_1	19	Transporter, Serotonin (5-Hydroxytryptamine) (SERT)	-4
Histamine H_2	0		

Data Report for Pharmacology Services

National Health Research Institutes
35 Keyan Rd, Zhunan Town, Miaoli County 350, Taiwan

Chia Ling Hsiao
886-37-246-166-35796
chhsiao@nhri.org.tw

Study #: TW04-0010577
Quote #: TW04-0010577-Q01 **t Study #:**
Study Date: Sep 28, 2021 - (Interim Report) **PO #:** N/A

Compound Information

**Panlabs Code:**	NHR-434
**Panlabs #:**	1251879
**Alt. Code 1:**	CN168
**Alt. Code 2:**	
**Alt. Code 3:**	
**M.W.:**	687.37
**F.W.:**	

Eurofins Panlabs Discovery Services Taiwan, Ltd.
25, Wugong 6th Road, Wugu District,
New Taipei City, Taiwan 24891

Tel: +886-2-7751-7000 • Fax:+886-2-2299-9375• e-mail: PanlabsTaipeiLab@eurofins.com

Cerep and Eurofins Panlabs are two companies of the Eurofins Group

Services Performed

LeadProfilingScreen (Total # of Assays: 67)

Study Objectives

To evaluate, in LeadProfilingScreen, the activity of test compound CN168 (Panlabs # 1251879).

Study Signatures

Yao-Chieh Chou

Study Director for Binding Assays

"This study was conducted according to the procedures described in this report. All data presented are authentic, accurate and correct to the best of our knowledge."

Table of Contents

Report Section	Page
Cover Page	1
Study Signatures	2
Table of Contents	3
Summary of Significant Results	4
Experimental Results	5

Summary of Significant Results

Biochemical assay results are presented as the percent inhibition of specific binding or activity throughout the report. All other results are expressed in terms of that assay's quantitation method.

- For primary assays, only the lowest concentration with a significant response judged by the assays' criteria, is shown in this summary.
- Where applicable, either the secondary assay results with the lowest dose/concentration meeting the significance criteria or, if inactive, the highest dose/concentration that did not meet the significance criteria is shown.
- Unless otherwise requested, primary screening in duplicate with quantitative data (e.g., IC_{50} ± SEM, K_i ± SEM and n_H) are shown where applicable for individual requested assays. In screening packages, primary screening in duplicate with semi-quantitative data (e.g., estimated IC_{50}, K_i and n_H) are shown where applicable (concentration range of 4 log units); available secondary functional assays are carried out (30 mM) and MEC or MIC determined only if active in primary assays >50% at 1 log unit below initial test concentration.

Significant responses (≥ 50% inhibition or stimulation for Biochemical assays) were noted in the primary assays listed below:

No significant results noted.

Experimental Results

Cat #	Assay Name	Batch*	Spec.	Rep.	Conc.	% Inh.	IC_{50}*	K_i	n_H	R
**Compound: CN168, PT #: 1251879**										
200510	Adenosine A_1	476987	hum	2	10 μM	-9				
200610	Adenosine A_{2A}	476944	hum	2	10 μM	5				
200720	Adenosine A_3	477021	hum	2	10 μM	0				
203110	Adrenergic α_{1A}	476973	hum	2	10 μM	-9				
203210	Adrenergic α_{1B}	476973	hum	2	10 μM	14				
203400	Adrenergic α_{1D}	477022	hum	2	10 μM	-6				
203630	Adrenergic α_{2A}	476988	hum	2	10 μM	-4				
204010	Adrenergic β_1	477216	hum	2	10 μM	4				
204110	Adrenergic β_2	477024	hum	2	10 μM	-4				
206000	Androgen (Testosterone)	477111	hum	2	10 μM	6				
212520	Bradykinin B_1	477217	hum	2	10 μM	-10				
212620	Bradykinin B_2	476993	hum	2	10 μM	-4				
214510	Calcium Channel L-Type, Benzothiazepine	477025	rat	2	10 μM	-13				
214600	Calcium Channel L-Type, Dihydropyridine	477043	rat	2	10 μM	14				
216000	Calcium Channel N-Type	476934	rat	2	10 μM	1				
217050	Cannabinoid CB_1	476975	hum	2	10 μM	-5				
219500	Dopamine D_1	477026	hum	2	10 μM	-2				
219700	Dopamine D_{2S}	477028	hum	2	10 μM	3				
219800	Dopamine D_3	477026	hum	2	10 μM	-3				
220000	Dopamine $D_{4.4}$	477029	hum	2	10 μM	12				
224010	Endothelin ET_A	477068	hum	2	10 μM	15				
224110	Endothelin ET_B	477136	hum	2	10 μM	-1				
225510	Epidermal Growth Factor (EGF)	476977	hum	2	10 μM	-1				
226010	Estrogen ERα	476935	hum	2	10 μM	-17				
226600	$GABA_A$, Flunitrazepam, Central	476990	rat	2	10 μM	-5				
226500	$GABA_A$, Muscimol, Central	477137	rat	2	10 μM	5				
228610	$GABA_{B1A}$	477031	hum	2	10 μM	14				
232030	Glucocorticoid	477112	hum	2	10 μM	14				
232710	Glutamate, Kainate	477032	rat	2	10 μM	0				
232810	Glutamate, NMDA, Agonism	477243	rat	2	10 μM	-3				
232910	Glutamate, NMDA, Glycine	476938	rat	2	10 μM	-5				
233000	Glutamate, NMDA, Phencyclidine	477051	rat	2	10 μM	-4				
239610	Histamine H_1	477054	hum	2	10 μM	19				

Note: Items meeting criteria for significance (≥50% stimulation or inhibition) are highlighted.
* Batch: Represents compounds tested concurrently in the same assay(s).
ham=Hamster; hum=Human

Experimental Results

Cat #	Assay Name	Batch*	Spec.	Rep.	Conc.	% Inh.	IC_{50}*	K_i	n_H	R
239710	Histamine H_2	476936	hum	2	10 µM	0				
239820	Histamine H_3	477029	hum	2	10 µM	2				
241000	Imidazoline I_2, Central	477139	rat	2	10 µM	-5				
243530	Interleukin IL-1 R1	477115	hum	2	10 µM	-3				
250460	Leukotriene, Cysteinyl $CysLT_1$	477245	hum	2	10 µM	-8				
251600	Melatonin MT_1	477140	hum	2	10 µM	6				
252610	Muscarinic M_1	477201	hum	2	10 µM	5				
252710	Muscarinic M_2	477202	hum	2	10 µM	-1				
252810	Muscarinic M_3	476980	hum	2	10 µM	0				
257010	Neuropeptide Y Y_1	476937	hum	2	10 µM	-4				
257110	Neuropeptide Y Y_2	477298	hum	2	10 µM	10				
258700	Nicotinic Acetylcholine α1, Bungarotoxin	477060	hum	2	10 µM	-6				
258730	Nicotinic Acetylcholine α3β4	476939	hum	2	10 µM	7				
260130	Opiate δ_1 (OP1, DOP)	477141	hum	2	10 µM	6				
260210	Opiate κ (OP2, KOP)	477142	hum	2	10 µM	3				
260410	Opiate μ (OP3, MOP)	477143	hum	2	10 µM	1				
264500	Phorbol Ester	477221	mouse	2	10 µM	-3				
299037	Platelet Activating Factor (PAF)	476931	hum	2	10 µM	-6				
265600	Potassium Channel [K_{ATP}]	477206	ham	2	10 µM	-1				
265900	Potassium Channel hERG	476971	hum	2	10 µM	-3				
268420	Prostanoid EP_4	477229	hum	2	10 µM	4				
299036	Purinergic P2X	476930	rat	2	10 µM	-9				
268820	Purinergic P2Y, Non-Selective	476928	rat	2	10 µM	9				
270000	Rolipram	476929	rat	2	10 µM	6				
271110	Serotonin (5-Hydroxytryptamine) $5\text{-}HT_{1A}$	476983	hum	2	10 µM	2				
271700	Serotonin (5-Hydroxytryptamine) $5\text{-}HT_{2B}$	476956	hum	2	10 µM	10				
271910	Serotonin (5-Hydroxytryptamine) $5\text{-}HT_3$	477250	hum	2	10 µM	-1				
299034	Sigma σ_1	477223	hum	2	10 µM	0				
279510	Sodium Channel, Site 2	476985	rat	2	10 µM	-1				
285900	Thyroid Hormone	477117	rat	2	10 µM	7				
220320	Transporter, Dopamine (DAT)	476989	hum	2	10 µM	0				
226400	Transporter, GABA	476926	rat	2	10 µM	2				
204410	Transporter, Norepinephrine (NET)	477120	hum	2	10 µM	-1				
274030	Transporter, Serotonin (5-Hydroxytryptamine) (SERT)	477300	hum	2	10 µM	-4				

Note: Items meeting criteria for significance (≥50% stimulation or inhibition) are highlighted.
* Batch: Represents compounds tested concurrently in the same assay(s).
ham=Hamster; hum=Human

Experimental Results

Eurofins Panlabs, Inc. has an exclusive, worldwide limited use license from Synaptic Pharmaceutical Corporation to perform these assays: Adrenergic Alpha 1D, Adrenergic Alpha 2B, and Dopamine D5 for safety and selectivity profiling. Eurofins Panlabs' license excludes performing those assays in connection with drug discovery or development activities where the principal therapeutic mechanism of action of the test compound involves selective binding to a licensed receptor. Customers may contact Synaptic directly if they believe they need a broader license.

Note: Items meeting criteria for significance (≥50% stimulation or inhibition) are highlighted.
* Batch: Represents compounds tested concurrently in the same assay(s).
ham=Hamster; hum=Human

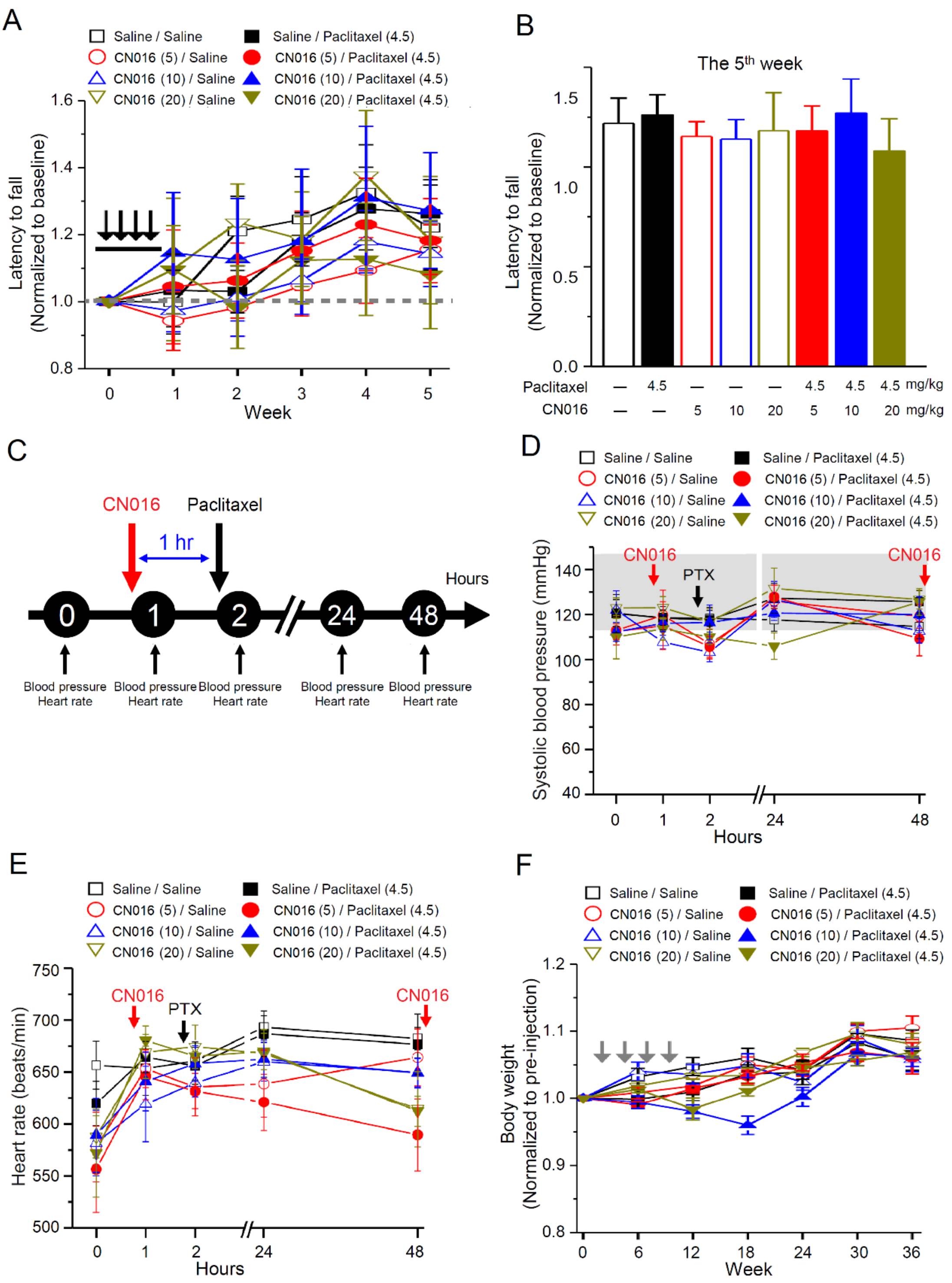

Figure S1. The safety assessment of CN016. (A) Rotarod test for the evaluation of motor function, motor learning, coordination, and equilibrium. Y axis, normalized latency to fall from the accelerating rotarod. Black arrows indicate the infusion of the drug. (B) Quantitative analyses of latency to fall at the 5th week. Each value represents mean ± S.E.M. from at least 5 mice in each group. Statistical significance was analyzed using two-way ANOVA. (C) Experimental design of hemodynamic assessment. (D) Systolic pressure after CN016 (5, 10 or 20 mg/kg) and paclitaxel (4.5 mg/kg) treatment. The gray box shows the normal range of systolic arterial pressure. Red and black arrows indicate the CN016 and paclitaxel injection. (E) Heart rate after CN016 (5, 10 or 20 mg/kg) and paclitaxel (4.5 mg/kg) treatment. Red and black arrows indicate the CN016 and paclitaxel injection. (F) Body weight for each group is normalized to body weight at 0 days. Gray arrows indicate the drug injection. Each value represents mean ± S.E.M. from at least 5 mice in each group.

A

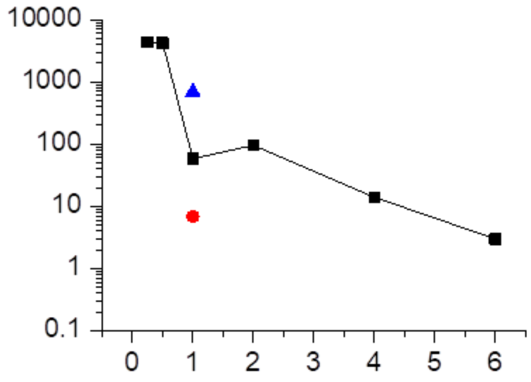

B

PK Study of CN016

mouse IP (dose: 5 mg/kg)			
$T_{1/2}$ (hr)	C_{max} (ng/ml)	T_{max} (hr)	$AUC_{(0\text{-}inf)}$ (ng/mL * hr)
1.9	5313	0.3	2273

Drug Conc. (ng/mL)		
Time (hr)	Avg.	S.D.
0.25	4293	3756
0.5	4220	985
1	58	54
2	96	45
4	14	13.9
6	3.1	3

Total number of mice: 18, N=3 for each time point

Figure S2. Pharmacokinetic profiles of CN016 (5 mg/kg) after intraperitoneal administration**.** (A) Plasma concentration-time profiles of the CN016 after intraperitoneal injection of a 5 mg/kg dose. Red Circle: a drug concentration of 10 nM. Blue Triangle: a drug concentration of 1000 nM. (B) Pharmacokinetic parameter of CN016 after intraperitoneal administration.

The pharmacokinetics of CN016 after IP administration (5 mg/kg) is illustrated in Figure S2. Behavior tests in mice were performed following CN016 first and then paclitaxel was given one hour later according to the protocol. As a result, the plasma concentration of CN016 was found to be 58 ng/mL (84.425 nM) at one hour, which was 8.4 times higher than that of *in vitro* effective drug concentration (10 nM) in the neurite outgrowth assay, thus rationalizing the observed efficacy in neurological behavior tests (Figure 4), in which CN016 (5 mg/kg) could significantly reduce the thermal insensitivity and mechanical pressure pain caused by paclitaxel. Black Square (plasma concentration from PK study, IP); Red Circle and Blue Triangle represented a drug concentration of 10 and 1000 nM, respectively, in the neurite outgrowth assay.

Synthesis of CN001~CN003, CN005~CN011, CN013~CN015, CN017~CN021, and CN025

Part I: Preparation of linkers 1-12

Linkers 1-12 used for synthesis of CN001~CN028 were listed below.

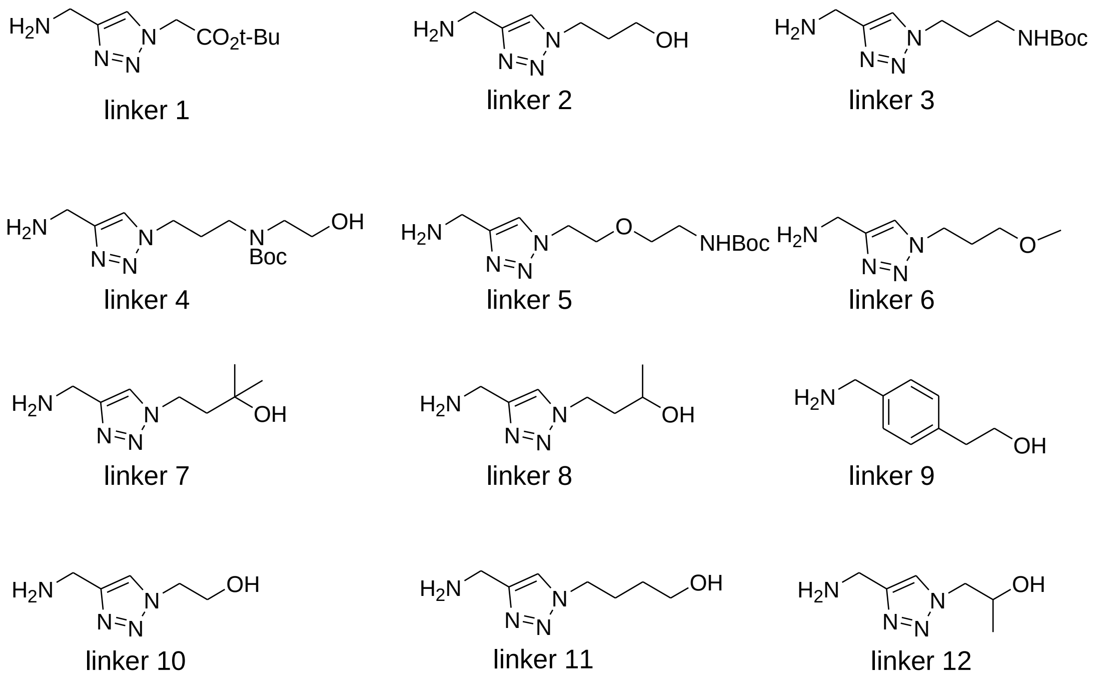

Preparation of linker 1

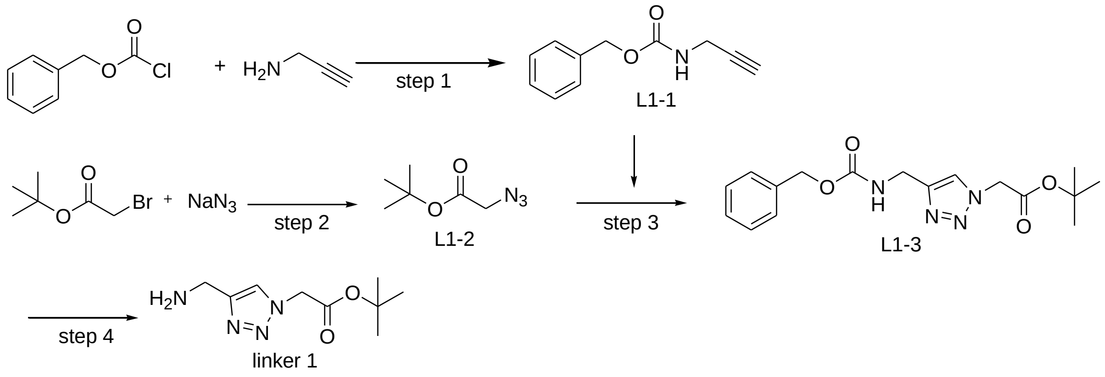

In the first step, benzyl chloroformate (6.07 g, 35.6 mmol) was added at 5-10 °C to a solution of prop-2-ynylamine (1.97 g, 35.8 mmol) and K_2CO_3 (10.11 g, 73.3 mmol) in a mixture of tetrahydrofuran and water (20 mL/40 mL). The resulting

mixture was warmed to room temperature for 15 h and then quenched with aqueous NH_4Cl, followed by extraction with ethyl acetate. The combined organic phases were washed with water and brine, dried over anhydrous sodium sulfate, filtered, and concentrated under reduced pressure to afford a residue. Crystallization of the residue using a solvent mixture of *n*-hexane/dichloromethane at -20 °C gave L1-1 (6.42 g, 95%). ^{1}H NMR (400 MHz, $CDCl_3$) δ 7.38−7.32 (m, 5H), 5.13 (s, 2H), 3.99 (m, 2H), 2.24 (dd, *J* = 2.8, 2.4 Hz, 1H).

A solution of bromo-acetic acid tert-butyl ester (5.85 g, 30 mmol) and sodium azide (19 g, 101 mmol) in acetone/H_2O (585 mL/140 mL) was stirred at 25 °C for 15 h and then concentrated to give a residue, which was extracted with DCM. The combined extracts were washed with brine, dried over anhydrous sodium sulfate, filtered, and concentrated to afford L1-2 (3.84 g, 81%). To a solution of L1-1 (4.56 g, 24.1 mmol) and L1-2 (3.84 g, 24.4 mmol) in ethanol (150 mL) was added a solution of $CuSO_4$ (0.41 g, 2.6 mmol), (+)-sodium L-ascorbate (0.56 g, 2.8 mmol), and K_2CO_3 (6.71 g, 48.5 mmol) in H_2O (36 mL). The mixture was stirred at 25 °C for 15 h and then concentrated to give a residue, which was extracted with DCM. The combined extracts were washed with brine, dried over anhydrous sodium sulfate, filtered, and concentrated to afford L1-3 (6.02 g, 71%). ^{1}H-NMR (300 MHz, $CDCl_3$) δ 7.62 (s, 1H), 7.38−7.32 (m, 5H), 5.13 (s, 2H), 5.02 (s, 2H), 4.63 (s, 2H), 1.44 (s, 9H).

A solution of L1-3 (3.01 g, 8.7 mmol) and 10% Pd/C (0.3 g) in methanol (60 mL) was stirred under H_2 (1 atm) at 25 °C for 6 h. The resulting mixture was then filtered. The filtrate was concentrated to afford linker 1 (1.64 g, 89%). ^{1}H-NMR (300 MHz, $CDCl_3$) δ 7.56 (s, 1H), 5.08 (s, 2H), 4.13 (s, 2H), 1.41 (s, 9H).

Preparation of linker 2

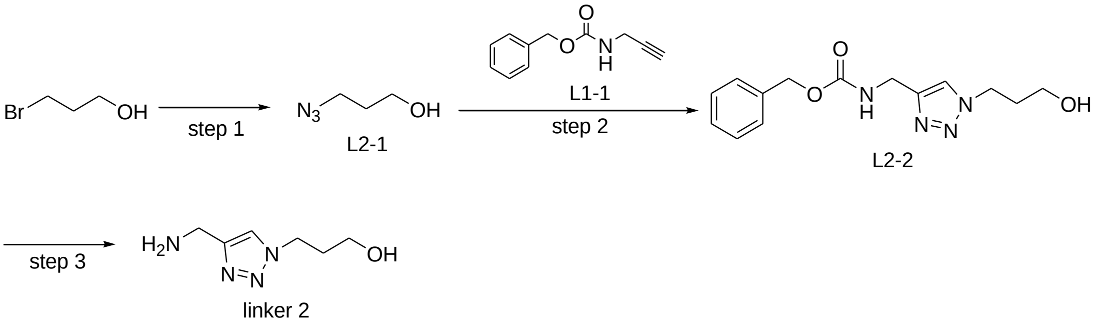

To a solution of 3-bromo-1-propanol (20 g, 144 mmol) in water/acetone (100 mL/35 mL) was added NaN_3 (11.3 g, 174 mmol) and KI (2.02 g, 12.2 mmol) at room temperature. The reaction mixture was stirred at 25 °C for 15 h and then concentrated. The resulting residue was extracted with ethyl acetate. The combined extracts were washed with water and brine, dried over anhydrous sodium sulfate, and filtered. The filtrate was concentrated to afford L2-1 (14.12 g, 97%). ^{1}H-NMR (400 MHz, $CDCl_3$) δ 3.77 (t, J = 6.0 Hz, 2H), 3.47 (t, J = 6.4 Hz, 2H), 1.85 (m, 2H).

To a solution of L1-1 (15.4 g, 81.4 mmol) and L2-1 (8.23 g, 81.4 mmol) in EtOH (800 mL) was added a solution of $CuSO_4$ (2.01 g, 12.6 mmol), (+)-sodium L-ascorbate (4 g, 20.2 mmol), and K_2CO_3 (16.02 g, 115.8 mmol) in H_2O (160 mL). The reaction mixture was stirred at 25 °C for 15 h and then concentrated. The resulting

residue was extracted with DCM. The combined extracts were washed with water and brine, dried over anhydrous sodium sulfate, filtered, and concentrated. The residue thus obtained in the previous step was purified by column chromatography on silica gel (*n*-hexane : ethyl acetate = 1 : 3) to afford L2-2 (19.74 g, 84%). ^{1}H-NMR (300 MHz, $CDCl_3$) δ 7.56 (s, 1H), 7.38−7.24 (m, 5H), 5.15 (s, 2H), 4.50−4.42 (m, 4H), 3.61 (m, 2H), 2.09 (m, 2H).

A solution of L2-2 (6 g, 20.7 mmol) and 10% Pd/C (1.2 g) in 2-propanol (120 mL) was stirred under H_2 (1 atm) at 60 °C for 15 h and filtered. The filtrate was concentrated to afford linker 2 (3.0 g, 93%). ^{1}H-NMR (400 MHz, $CDCl_3$) δ 7.55 (s, 1H), 4.43 (t, *J* = 6.8 Hz, 2H), 3.89 (s, 2H), 3.53 (t, *J* = 6.0 Hz, 2H), 2.04 (m, 2H).

Preparation of linker 3

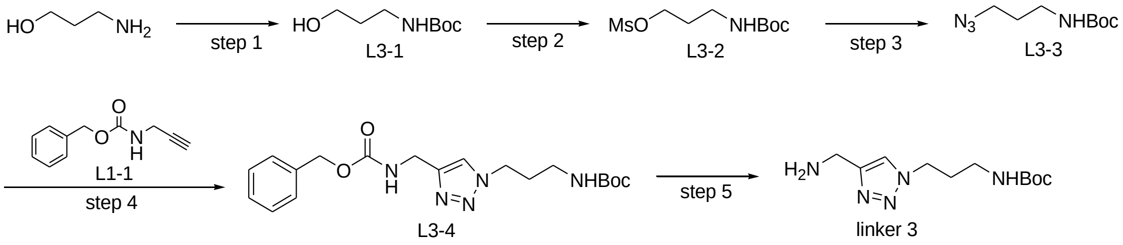

To a solution of 1-aminopropanol (2.25 g, 30 mmol) in DCM was added tert-butoxycarbonyl anhydride (7.81 g, 35.7 mmol) and TEA (3.6 g, 35.6 mmol). The mixture was stirred at room temperature for 15 h and then concentrated to give a crude residue, which was purified by column chromatography on silica gel (*n*-hexane : ethyl acetate = 1 : 3) to afford L3-1 (4.11 g, 78%). ^{1}H-NMR (300 MHz, $CDCl_3$) δ 3.65 (m, 2H), 3.27 (m, 2H), 1.65 (m, 2H), 1.44 (s, 9H).

To a solution of L3-1 (4.11 g, 23.4 mmol) and TEA (3.22 g, 31.7 mmol) in DCM (180 mL) was added methanesulfonyl chloride (5.41 g, 47.2 mmol) dropwise at 5-10 °C. The resulting mixture was warmed to room temperature for 15 h and then quenched with aqueous NH_4Cl, followed by extraction with DCM. The combined organic phases were washed with aqueous $NaHCO_3$ and brine, dried over anhydrous sodium sulfate, filtered, and concentrated to afford L3-2 (4.12 g, 70%). To a solution of L3-2 (4.12 g, 16.3 mmol) in water/acetone (100 mL/35 mL) was added NaN_3 (5.41 g, 83.2 mmol) and KI (0.3 g, 1.8 mmol) at room temperature. The reaction mixture was stirred at 25 °C for 15 h and then concentrated. The resulting residue was extracted with ethyl acetate. The combined extracts were washed with water and brine, dried over anhydrous sodium sulfate, and filtered. The filtrate was concentrated to afford L3-3 (2.41 g, 74%).^{1}H-NMR (300 MHz, $CDCl_3$) δ 3.35 (t, J = 6.6 Hz, 2H), 3.23 (m, 2H), 1.77 (m, 2H), 1.44 (s, 9H)

To a solution of L3-3 (2.41 g, 12 mmol) and L1-1 (2.41 g, 12.7 mmol) in EtOH (120 mL) was added a solution of $CuSO_4$ (0.2 g, 1.3 mmol), (+)-sodium L-ascorbate (0.24 g, 1.2 mmol), K_2CO_3 (2.24 g, 16.2 mmol) in H_2O (24 mL). The mixture was stirred at 25 °C for 15 h and then concentrated to give a residue, which was extracted with DCM. The combined extracts were washed with brine, dried over anhydrous sodium sulfate, filtered, and concentrated to afford L3-4 (3.12 g, 67%). ^{1}H-NMR (400

MHz, $CDCl_3$) δ 7.35 (s, 1H), 7.37−7.32 (m, 5H), 5.11 (s, 2H), 4.46 (d, J = 6.0 Hz, 2H), 4.39 (t, J = 6.8 Hz, 2H), 3.12 (m, 2H), 2.07 (m, 2H), 1.46 (s, 9H).

A solution of L3-4 (3.12 g, 8.0 mmol) and 10% Pd/C (0.6 g) in 2-propanol (62 mL) was stirred under H_2 (1 atm) at 25 °C for 6 h and then filtered. The filtrate was concentrated to afford linker 3 (1.82 g, 89%). ^{1}H-NMR (400 MHz, $CDCl_3$) δ 7.56 (s, 1H), 4.41 (t, J = 6.8 Hz, 2H), 3.99 (s, 2H), 3.14 (m, 2H), 2.09 (m, 2H), 1.46 (s, 9H).

Preparation of linker 4

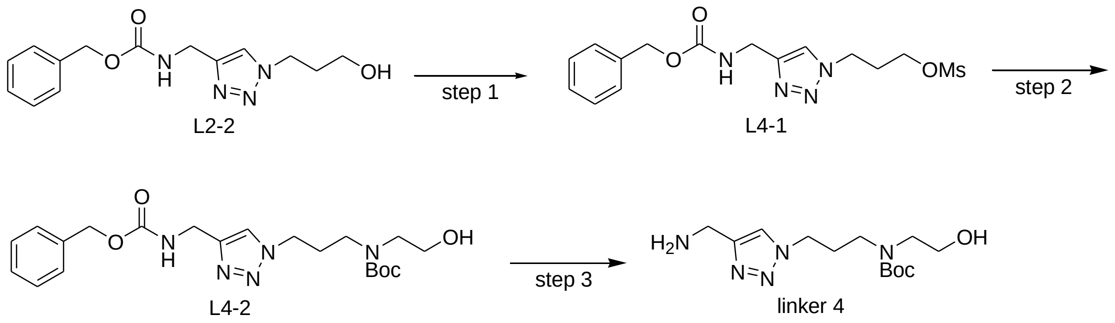

To a solution of L2-2 (5 g, 17.2 mmol) in dry DCM (100 mL) was added TEA (2.6 g, 25.7 mmol) and MsCl (2.37 g, 20.7 mmol) at 5 °C. The reaction mixture was stirred at 25 °C for 15h and then quenched with aqueous NH_4Cl. The resulting residue was extracted with DCM. The combined extracts were washed with water and brine, dried over anhydrous sodium sulfate, and filtered. The filtrate was concentrated to afford L4-1 (4.56 g, 72%). ^{1}H-NMR (300 MHz, $CDCl_3$) δ 7.58 (s, 1H), 7.40−7.24 (m, 5H), 5.11 (s, 2H), 4.58−4.46 (m, 4H), 4.23 (m, 2H), 3.04 (s, 3H), 2.36 (m, 2H).

To a solution of L4-1 (4.56 g, 12.4 mmol) in dry THF (50 mL) was added 2-aminoethanol (5.6 g, 91.7 mmol) at 5 °C. The reaction mixture was stirred at 65 °C for

15 h and then quenched with aqueous NH_4Cl. The resulting mixture was extracted with ethyl acetate. The combined extracts were washed with water and brine, dried over anhydrous sodium sulfate, filtered, and concentrated. The residue thus obtained in the previous step was dissolved in DCM and mixed with Boc_2O (3.4 g, 15.6 mmol) and TEA (1.6 g, 15.8 mmol). The mixture was stirred at room temperature for 15 h and then concentrated to give a crude residue, which was purified by column chromatography on silica gel (*n*-hexane : ethyl acetate = 1 : 3) to afford L4-2 (3.51 g, 65%). ^{1}H-NMR (400 MHz, $CDCl_3$) δ 7.60 (s, 1H), 7.39−7.32 (m, 5H), 5.11 (s, 2H), 4.46 (d, J = 6.0 Hz, 2H), 4.36 (t, J = 6.8 Hz, 2H), 3.73 (m, 2H), 3.37−3.31 (m, 4H), 2.18 (m, 2H), 1.46 (s, 9H).

A solution of L4-2 (3.51 g, 8.1 mmol) and 10% Pd/C (1.0 g) in 2-propanol (100 mL) was stirred under H_2 (1 atm) at 60 °C for 15 h and filtered. The filtrate was concentrated to afford linker 4 (2.2 g, 91%). ^{1}H-NMR (400 MHz, $CDCl_3$) δ 7.53 (s, 1H), 4.37 (t, J = 6.8 Hz, 2H), 3.97 (s, 2H), 3.71 (t, J = 5.2 Hz, 2H), 3.41-3.30 (m, 4H), 2.16 (m, 2H), 1.45 (s, 9H).

Preparation of linker 5

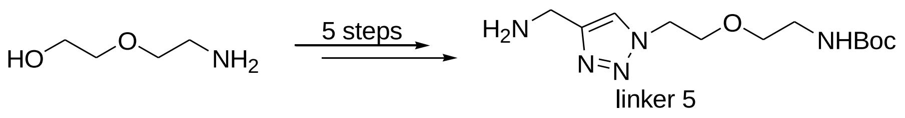

Linker 5 was prepared from 2-(2-aminoethoxy)ethanol following a synthetic

procedure similar to that used for linker 3 and obtained in 19% yield over five steps. ^{1}H-NMR (400 MHz, $CDCl_3$) δ 7.60 (s, 1H), 4.49 (t, *J* = 4.8 Hz, 2H), 3.99 (s, 2H), 3.80 (t, *J* = 4.8 Hz, 2H), 3.46 (t, *J* = 4.8 Hz, 2H), 3.25 (m, 2H), 1.41 (s, 9H).

Preparation of linker 6

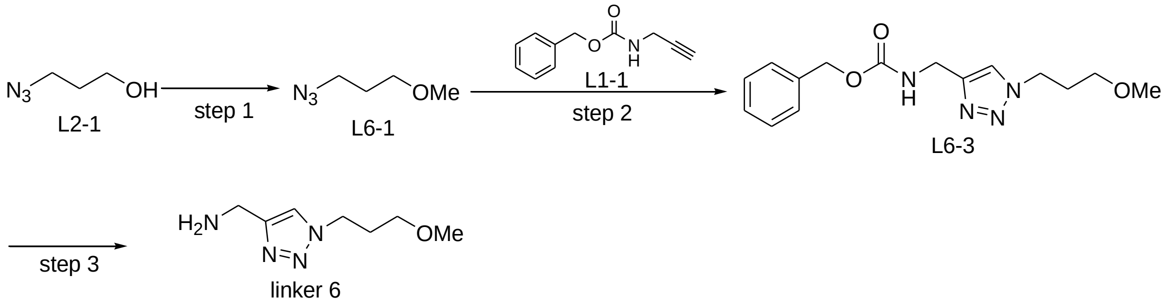

To a solution of L2-1 (5 g, 49.5 mmol) in solution of dry dimethylformamide (40 mL) was added KOH (11 g, 196.4 mmol) at 5 °C. The reaction mixture was stirred at 5 °C for 0.5 h and then iodomethane (14 g, 98.6 mmol) was then added to the solution. The mixture was stirred at room temperature for 15 h and concentrated. The resulting residue was extracted with ethyl acetate. The combined extracts were washed with water and brine, dried over anhydrous sodium sulfate, and filtered. The filtrate was concentrated to afford L6-1 (1.62 g, 28%). ^{1}H-NMR (400 MHz, $CDCl_3$) δ 3.46 (t, *J* = 6.4 Hz, 2H), 3.39 (t, *J* = 6.8 Hz, 2H), 3.35 (s, 3H), 1.85 (m, 2H).

To a solution of L6-1 (1.62 g, 14.1 mmol) and L1-1 (2.67 g, 14.1 mmol) in EtOH (100 mL) was added a solution of $CuSO_4$ (0.34 g, 2.1 mmol), (+)-sodium L-ascorbate (0.84 g, 4.2 mmol), and K_2CO_3 (1.94 g, 14 mmol) in H_2O (20 mL). The reaction

mixture was stirred at 25 °C for 15 h and then concentrated. The resulting residue was extracted with DCM. The combined extracts were washed with water and brine, dried over anhydrous sodium sulfate, filtered, and concentrated. The residue thus obtained in the previous step was purified by column chromatography on silica gel (*n*-hexane : ethyl acetate = 1 : 2) to afford L6-2 (3.03 g, 71%). ^{1}H-NMR (400 MHz, $CDCl_3$) δ 7.52 (s, 1H), 7.40–7.32 (m, 5H), 5.11 (s, 2H), 4.46 (d, J = 6.0 Hz, 2H), 4.43 (t, J = 6.8 Hz, 2H), 3.36 (m, 2H), 3.32 (s, 3H), 2.16 (m, 2H).

A solution of L6-2 (3.03 g, 10.0 mmol) and 10% Pd/C (0.6 g) in 2-propanol (60 mL) was stirred under H_2 (1 atm) at 60 °C for 15 h and filtered. The filtrate was concentrated to afford linker 6 (1.66 g, 98%). ^{1}H-NMR (400 MHz, $CDCl_3$) δ 7.44 (s, 1H), 4.45 (t, J = 6.8 Hz, 2H), 4.01 (s, 2H), 3.36 (t, J = 6.0 Hz, 2H), 3.34 (s, 3H), 1.70 (m, 2H).

Preparation of linker 7

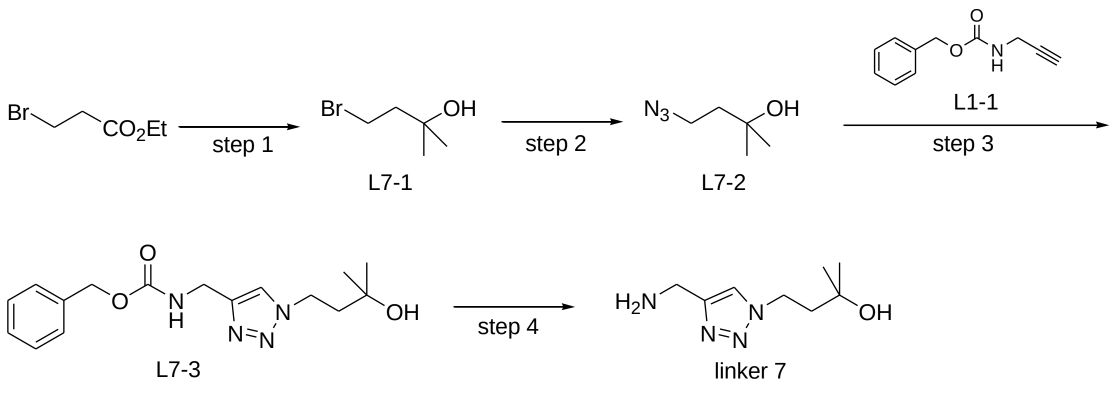

To a solution of ethyl 3-bromopropanoate (2 g, 11 mmol) in dry THF (45 mL) was added a solution of methylmagnesium bromide (6 mL, 3 M in THF). The mixture

was stirred at 25 °C for 4 h and then quenched with aqueous NH_4Cl. The resulting mixture was extracted with ethyl acetate. The combined extracts were washed with brine, dried over anhydrous sodium sulfate, filtered, and concentrated to afford L7-1 (1.21 g, 65%) ^{1}H-NMR (400 MHz, $CDCl_3$) δ 3.49 (m, 2H), 2.14 (m, 2H), 1.22 (s, 6H).

To a solution of L7-1 (1.21 g, 7.2 mmol) in dimethylformamide (12 mL) was added NaN_3 (2.31 g, 35.4 mmol) and Na_2CO_3 (2.02 g, 19.1 mmol) at room temperature. The reaction mixture was stirred at 25 °C for 15 h and then concentrated. The resulting residue was extracted with ethyl acetate. The combined extracts were washed with water and brine, dried over anhydrous sodium sulfate, and filtered. The filtrate was concentrated to afford L7-2 (0.72 g, 78%). ^{1}H-NMR (400 MHz, $CDCl_3$) δ 3.41 (m, 2H), 1.74 (m, 2H), 1.22 (s, 6H).

To a solution of L7-2 (0.72 g, 5.6 mmol) and L1-1 (1.56 g, 8.2 mmol) in EtOH (45 mL) was added a solution of $CuSO_4$ (0.24 g, 1.5 mmol), (+)-sodium L-ascorbate (0.48 g, 2.4 mmol), and K_2CO_3 (0.84 g, 6.1 mmol) in H_2O (9 mL). The reaction mixture was stirred at 25 °C for 15 h and then concentrated. The resulting residue was extracted with DCM. The combined extracts were washed with water and brine, dried over anhydrous sodium sulfate, filtered, and concentrated. The residue thus obtained in the previous step was purified by column chromatography on silica gel (MeOH :

ethyl acetate = 1 : 19) to afford L7-3 (1.2 g, 68%). ^{1}H-NMR (400 MHz, $CDCl_3$) δ 7.60 (s, 1H), 7.38−7.28 (m, 5H), 5.08 (s, 2H),4.47 (m, 2H), 4.40 (s, 2H), 2.06 (m, 2H), 1.31 (s, 6H).

A solution of L7-3 (1.2 g, 3.8 mmol) and 10% Pd/C (0.24 g) in 2-propanol (24 mL) was stirred under H_2 (1 atm) at 60 °C for 15 h and then filtered. The filtrate was concentrated to afford linker 7 (0.61 g, 88%). ^{1}H-NMR (400 MHz, $CDCl_3$) δ 7.61 (s, 1H), 4.51 (m, 2H), 4.00 (s, 2H), 2.11 (m, 2H), 1.31 (s, 6H).

Preparation of linker 8

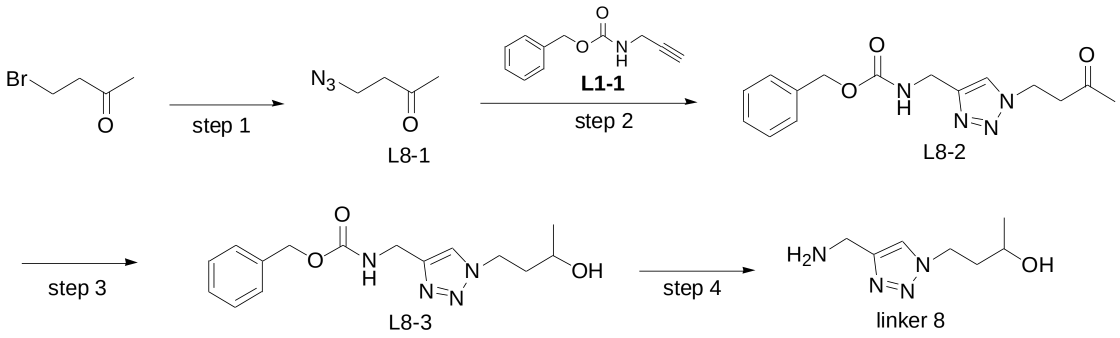

To a solution of 4-bromo-butan-2-one (1.51 g, 10 mmol) in dimethylformamide (16 mL) was added NaN_3 (3.01 g, 46.2 mmol) and KI (0.23 g, 1.4 mmol) at room temperature. The reaction mixture was stirred at 25 °C for 15 h and then concentrated. The resulting residue was extracted with ethyl acetate. The combined extracts were washed with water and brine, dried over anhydrous sodium sulfate, and filtered. The filtrate was concentrated to afford L8-1 (0.87 g, 77%).^{1}H-NMR (400 MHz, $CDCl_3$) δ 3.54 (t, J = 6.4 Hz, 2H), 2.70 (t, J = 6.4 Hz, 2H), 2.04 (s, 3H).

To a solution of L8-1 (0.87 g, 7.7 mmol) and L1-1 (1.7 g, 9 mmol) in EtOH (65

mL) was added a solution of $CuSO_4$ (0.4 g, 2.5 mmol), (+)-sodium L-ascorbate (0.75 g, 3.8 mmol), and K_2CO_3 (1.2 g, 8.7 mmol) in H_2O (13 mL). The reaction mixture was stirred at 25 °C for 15 h and then concentrated. The resulting residue was extracted with DCM. The combined extracts were washed with water and brine, dried over anhydrous sodium sulfate, filtered, and concentrated. The residue thus obtained in the previous step was purified by column chromatography on silica gel (*n*-hexane : ethyl acetate = 1 : 3) to afford L8-2 (1.98 g, 85%). ^{1}H-NMR (400 MHz, $CDCl_3$) δ 7.61 (s, 1H), 7.40−7.31 (m, 5H), 5.13 (s, 2H), 4.60 (t, *J* = 6.4 Hz, 2H), 4.46 (d, *J* = 6.0 Hz, 2H), 3.14 (m, 2H), 2.21 (s, 3H).

To a solution of L8-2 (1.98 g, 6.5 mmol) in dry MeOH (30 mL) was added $NaBH_4$ (0.72 g, 19 mmol) at 5 °C. The reaction mixture was stirred at 5 °C for 3 h and then quenched with aqueous NH_4Cl. The resulting mixture was extracted with ethyl acetate. The combined extracts were washed with water and brine, dried over anhydrous sodium sulfate, and filtered. The filtrate was concentrated to afford L8-3 (1.61 g, 81%). ^{1}H-NMR (400 MHz, $CDCl_3$) δ 7.60 (s, 1H), 7.38−7.28 (m, 5H), 5.09 (s, 2H), 4.47 (m, 2H), 4.42 (d, *J* = 6.4 Hz, 2H), 3.73 (m, 1H), 2.02 (m, 1H), 1.92 (m, 1H), 1.21 (d, *J* = 6.0 Hz, 3H).

A solution of L8-3 (1.61 g, 5.3 mmol) and 10% Pd/C (0.32 g) in 2-propanol (32 mL) was stirred under H_2 (1 atm) at 60 °C for 15 h and filtered. The filtrate was

concentrated to afford linker 8 (0.83 g, 93%). ^{1}H-NMR (400 MHz, $CDCl_3$) δ 7.53 (s, 1H), 4.55 (m, 1H), 4.44 (m, 1H), 3.99 (s, 2H), 3.74 (m, 1H), 2.06 (m, 1H), 1.94 (m, 1H), 1.20 (d, J = 6.4 Hz, 3H).

Preparation of linker 9

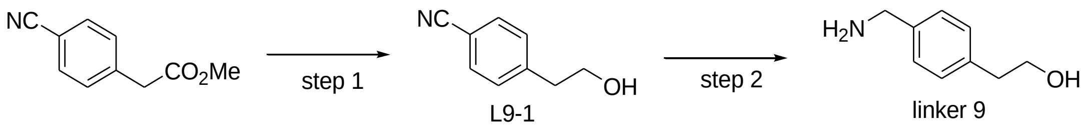

To a solution of methyl (4-cyanophenyl)acetate (2.0 g, 11.4 mmol) in dry THF (50 mL) was added lithium aluminum hydride (0.85 g, 22.4 mmol) at 5 °C. The reaction mixture was stirred at 5 °C for 2 h and then quenched with aqueous NH_4Cl. The resulting mixture was extracted with ethyl acetate. The combined extracts were washed with water and brine, dried over anhydrous sodium sulfate, and filtered. The filtrate was concentrated to afford L9-1 (1.42 g, 85%). (400 MHz, $CDCl_3$) δ 7.62 (d, J = 8.0 Hz, 2H), 7.37 (d, J = 8.0 Hz, 2H), 3.91 (t, J = 6.4 Hz, 2H), 2.95 (t, J = 6.4 Hz, 2H).

To a solution of L9-1 (1.42 g, 9.6 mmol) in MeOH (45 mL) was added $NiCl_2$ (0.14 g, 1.1 mmol) and $NaBH_4$ (0.75 g, 19.8 mmol), and the mixture was stirred at 0 °C for 4 h and then quenched with 1N aqueous HCl solution. The mixture was concentrated to remove MeOH, and the resulting mixture was extracted with DCM. The combined organic extracts were washed with brine, dried over anhydrous sodium sulfate, and filtered. The filtrate was concentrated to afford linker 9 (1.04 g, 71%).

(400 MHz, $CDCl_3$) δ 7.27 (d, *J* = 8.0 Hz, 2H), 7.20 (d, *J* = 8.0 Hz, 2H), 3.93 (s, 2H), 3.83 (t, *J* = 6.0 Hz, 2H), 2.82 (t, *J* = 6.0 Hz, 2H).

Preparation of linker 10

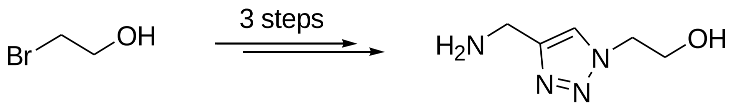

linker 10

Linker 10 was prepared from 2-bromoethnol following a synthetic procedure similar to that used for linker 2 and obtained in 65% yield over three steps. ^{1}H-NMR (400 MHz, $CDCl_3$) δ 7.56 (s, 1H), 4.52 (t, *J* = 4.8 Hz, 2H), 4.20 (t, *J* = 4.8 Hz, 2H), 4.00 (s, 2H).

Preparation of linker 11

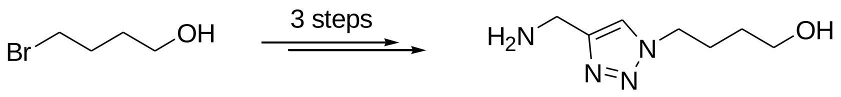

linker 11

Linker 11 was prepared from 4-bromobutanol following a synthetic procedure similar to that used for linker 2 and obtained in 71% yield over three steps. ^{1}H-NMR (400 MHz, $CDCl_3$) δ 7.53 (s, 1H), 4.43 (t, *J* = 7.2 Hz, 2H), 4.01 (s, 2H), 3.46 (t, *J* = 6.0 Hz, 2H), 1.51−1.46 (m, 4H).

Preparation of linker 12

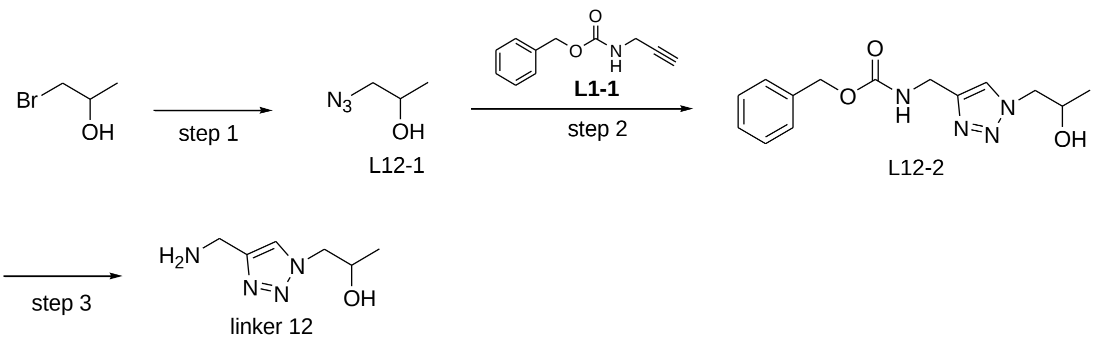

To a solution of 1-bromo-2-propanol (1.69 g, 12.2 mmol) in dimethylformamide (16 mL) was added NaN_3 (3.01 g, 46.2 mmol) and Na_2CO_3 (2.32 g, 21.7 mmol) at room temperature. The reaction mixture was stirred at 25 °C for 15 h and then concentrated. The resulting residue was extracted with ethyl acetate. The combined extracts were washed with water and brine, dried over anhydrous sodium sulfate, and filtered. The filtrate was concentrated to afford L12-1 (0.98 g, 80%). ^{1}H-NMR (300 MHz, $CDCl_3$) δ 3.95 (m, 1H), 3.36 (dd, *J* = 12.3, 3.3 Hz, 1H), 3.23 (dd, *J* = 12.3, 7.5 Hz, 1H), 1.22 (d, *J* = 6.3 Hz, 3H).

To a solution of L12-1 (0.98 g, 9.7 mmol) and L1-1 (1.9 g, 10.0 mmol) in EtOH (95 mL) was added a solution of $CuSO_4$ (0.2 g, 2.5 mmol), (+)-sodium L-ascorbate (0.48 g, 3.8 mmol), and K_2CO_3 (1.8 g, 8.7 mmol) in H_2O (19 mL). The reaction mixture was stirred at 25 ℃ for 15 h and then concentrated. The resulting residue was extracted with DCM. The combined extracts were washed with water and brine, dried over anhydrous sodium sulfate, filtered, and concentrated. The residue thus obtained in the previous step was purified by column chromatography on silica gel (n-

hexane : ethyl acetate = 1 : 3) to afford L12-2 (1.81 g, 64%). ^{1}H-NMR (300 MHz, $CDCl_3$) δ 7.62 (s, 1H), 7.40−7.27 (m, 5H), 5.12 (s, 2H), 4.76 (d, *J* = 6.0 Hz, 2H), 4.40 (m, 1H), 4.24 (m, 1H), 4.20 (m, 1H), 1.25 (d, *J* = 6.3 Hz, 3H).

A solution of L12-2 (1.81 g, 6.2 mmol) and 10% Pd/C (0.36 g) in 2-propanol (32 mL) was stirred under H_2 (1 atm) at 60 ℃ for 15 h and filtered. The filtrate was concentrated to afford linker 12 (0.89 g, 91%). ^{1}H-NMR (400 MHz, $CDCl_3$) δ 7.56 (s, 1H), 4.42 (dd, *J* = 12.3, 3.0 Hz, 1H), 4.30−4.18 (m, 2H), 3.98 (s, 2H), 1.22 (d, *J* = 6.3 Hz, 3H).

Part II: Synthesis of CN001~CN003, CN005~CN011, CN013~CN015, CN017~CN021, and CN025

Preparation of CN001

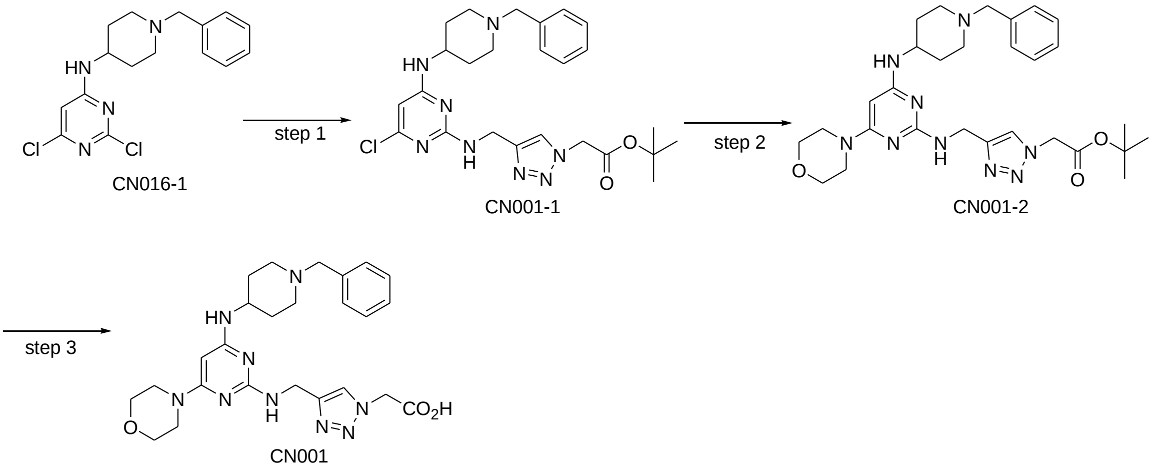

A solution of CN016-1 (1.02 g, 3.0 mmol) and linker 1 (1.06 g, 5.0 mmol) in 1-pentanol (20 mL) was heated at 140 °C for 15 hours. The resulting mixture was then

concentrated to afford crude CN001-1. To a solution of CN001-1 in 2-propanol (20 mL) was added morpholine (0.82 g, 9.4 mmol). The mixture was stirred at 120 °C for 15 h and then concentrated. The residue thus obtained in the previous step was purified by column chromatography on silica gel (MeOH : ethyl acetate = 1 : 9) to afford CN001-2 (0.87 g, 51%). ^{1}H NMR (400 MHz, $CDCl_3$) δ 7.59 (s, 1H), 7.36–7.20 (m, 5H), 5.07 (s, 2H), 5.02 (s, 1H), 4.69 (d, J = 6.0Hz, 2H), 4.38 (m, 1H), 3.73 (m, 4H), 3.55 (m, 1H), 3.51 (s, 2H), 3.45 (m, 4H), 2.80 (m, 2H), 2.16 (m, 2H), 1.96 (m, 2H), 1.53 (m, 2H), 1.46 (s, 9H).

A solution of 2N HCl/diethyl ether (1 mL, 2 mmol) was added to a DCM of CN001-2 (218 mg, 0.4 mmol). The reaction mixture was stirred at 25 °C for 15 h and then concentrated to afford a hydrochloride salt of CN001 (179 mg, 85%).

Preparation of CN002

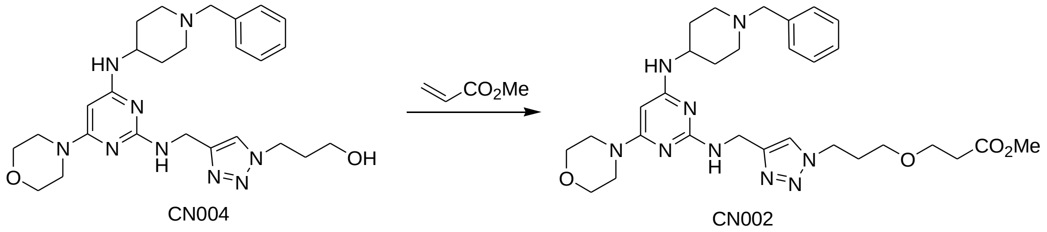

A solution of CN004 (218 mg, 0.4 mmol), methyl acrylate (128 mg, 1.5 mmol), and TEA (65 mg, 0.6 mmol) in MeOH (50 mL) was stirred at 60 °C for 16 h and then concentrated. The residue thus obtained in the previous stepwas purified by column chromatography on silica gel (MeOH : ethyl acetate = 1 : 4) to afford CN002 (173 mg, 68%).

Preparation of CN003

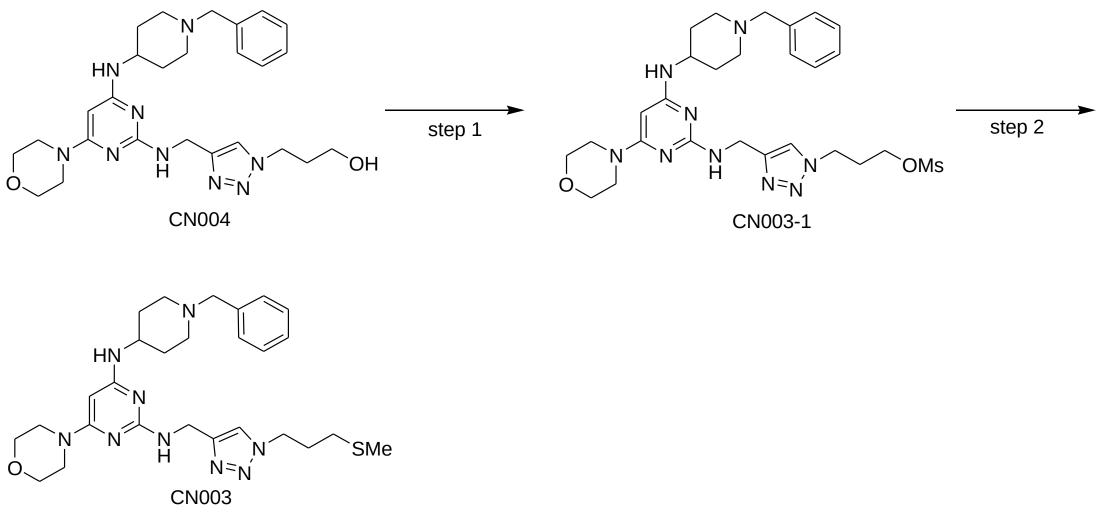

To a solution of CN004 (217 mg, 0.4 mmol) in dry DCM (6 mL) was added TEA (132 mg, 1.3 mmol) and MsCl (104 mg, 0.9 mmol) at 5 °C. The reaction mixture was stirred at 25 °C for 15 h and then quenched with aqueous NH_4Cl. The resulting residue was extracted with DCM. The combined extracts were washed with water and brine, dried over anhydrous sodium sulfate, and filtered. The filtrate was concentrated to afford CN003-1 (181 mg, 72%). To a solution of CN003-1 (181 mg, 0.6 mmol) in dimethylformamide (3 mL) was added aqueous NaSMe (0.31 mL, 0.7 mmol, 15% in H_2O). The reaction mixture was stirred at 45 °C for 3 h and then quenched with aqueous NH_4Cl. The resulting residue was extracted with DCM. The combined extracts were washed with water and brine, dried over anhydrous sodium sulfate, filtered and concentrated. The residue thus obtained in the previous stepwas purified by column chromatography on silica gel (MeOH : ethyl acetate = 1 : 4) to afford CN003 (124 mg, 75%).

Preparation of CN005

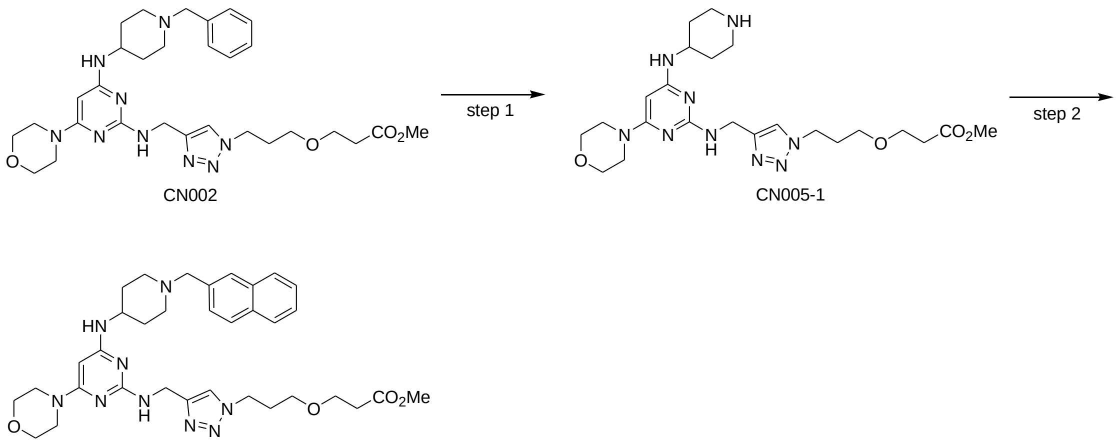

CN005

A solution of CN002 (171mg, 0.3 mmol) and 10% Pd/C (34 mg) in 2-propanol (5 mL) was stirred under H_2 (1 atm) at 60 ºC for 15 h and then filtered. The filtrate was concentrated to afford CN005-1 (132 mg, 91%). ^{1}H NMR (400 MHz, CD_3OD) δ 7.78 (s, 1H), 4.53 (s, 2H), 4.46 (t, *J* = 6.8 Hz, 2H), 3.75 (m, 4H), 3.73 (m, 1H), 3.70 (s, 3H), 3.52 (t, *J* = 6.0 Hz, 2H), 3.46 (m, 2H), 3.11 (m, 2H), 3.02 (m, 4H), 2.75 (t, *J* = 6.8 Hz, 2H), 2.52 (t, *J* = 6.8 Hz, 2H), 2.1–2.03 (m, 4H), 1.75 (m, 2H).

A solution of CN005-1 (132 mg, 0.3 mmol), 2-Bromomethyl-naphthalene (117 mg, 0.5 mmol), and TEA (83 mg, 0.8 mmol) in MeOH (4 mL) was stirred at 25 °C for 16 h and then quenched with aqueous NH_4Cl. The resulting mixture was extracted with ethyl acetate. The combined extracts were washed with water and brine, dried over anhydrous sodium sulfate, filtered, and concentrated. The residue was purified by column chromatography on silica gel (MeOH : ethyl acetate = 1 : 4) to afford CN005 (128 mg, 75%).

Preparation of CN006

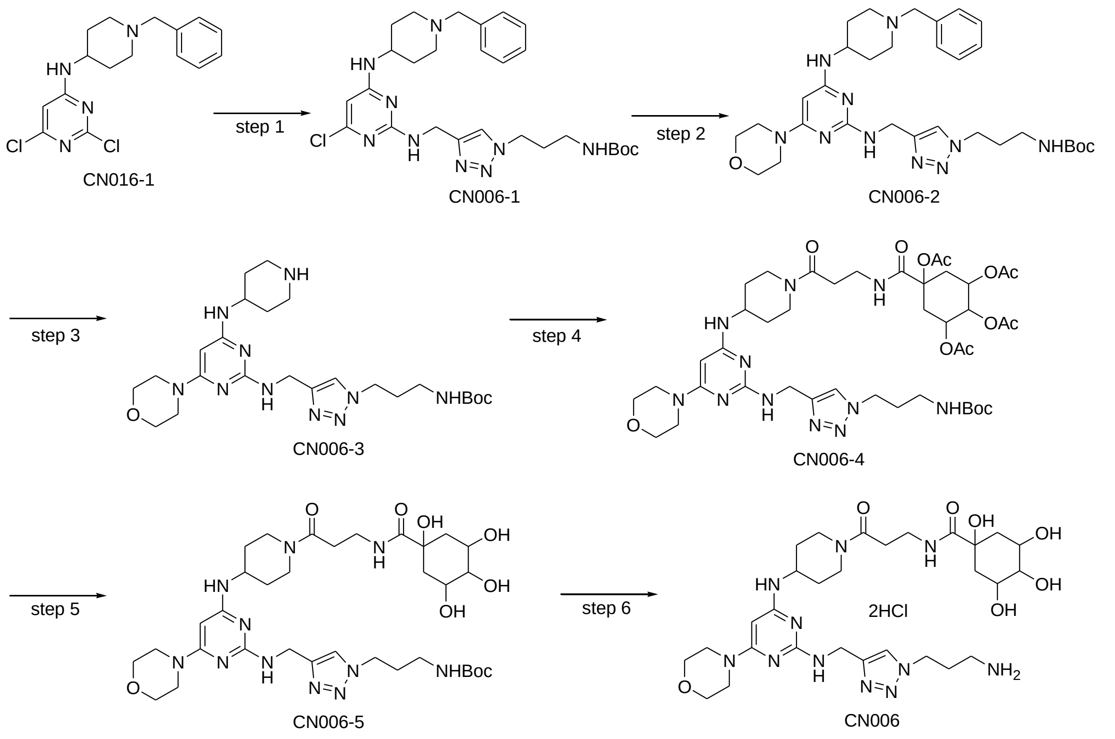

To a solution of CN016-1 (1.83 g, 5.4 mmol) in 1-pentanol (50 mL) was added linker 3 (1.41 g, 5.5 mmol). The mixture was stirred at 140 °C for 15 hours and then concentrated. The residue thus obtained in the previous step was purified by column chromatography on silica gel with ethyl acetate to afford CN006-1 (2.01 g, 67%). ^{1}H NMR (400 MHz, $CDCl_3$) δ 7.54 (s, 1H), 7.36–7.32 (m, 5H), 4.84 (s, 1H), 4.65 (d, *J* = 5.6 Hz, 2H), 4.39 (*J* = 4.8 Hz, 2H), 3.50 (s, 2H), 3.12 (m, 2H), 2.82 (m, 2H), 2.11 (m, 2H), 1.93 (m, 2H), 1.73–1.57 (m, 4H), 1.45 (s, 9H).

To a solution of CN006-1 (2.01 g, 3.6 mmol) in 1-pentanol (40 mL) was added morpholine (1.42 g, 16.1 mmol). The mixture was stirred at 120 °C for 15 h and then concentrated. The residue thus obtained in the previous step was purified by column

chromatography on silica gel (MeOH : ethyl acetate = 1 : 19) to afford CN006-2 (1.83, 83%). ^{1}H NMR (400 MHz, $CDCl_3$) δ 7.50 (s, 1H), 7.36–7.32 (m, 5H), 4.93 (s, 1H), 4.65 (d, J = 5.6 Hz, 2H), 4.38 (J = 5.2 Hz, 2H), 3.73 (m, 4H), 3.51 (s, 2H), 3.46–3.43 (m, 5H), 3.12 (m, 2H), 2.82 (m, 2H), 2.04 (m, 2H), 1.95 (m, 2H), 1.73–1.57 (m, 4H), 1.44 (s, 9H).

A solution of CN006-2 (1 g, 1.6 mmol) and 10% Pd/C (0.3 g) in 2-propanol (20 mL) was stirred under H_2 (1 atm) at 60 °C for 15 h. The resulting mixture was filtered and concentrated to afford CN006-3 (0.74 g, 87%). To a solution of CN006-3 (190mg, 0.4 mmol) in DCM (8 mL) was added 1-ethyl-3-(3-dimethylaminopropyl)-carbodiimide (145 mg, 0.8 mmol), hydroxybenzotriazole (92 mg, 0.6 mmol) and 3-[(1,3,4,5-Tetraacetoxy-cyclohexanecarbonyl)-amino]-propionic acid (300 mg, 0.7 mmol) at 25°C. The reaction mixture was stirred for 15 h and then poured into water. The resulting mixture was extracted with DCM. The combined organic extracts were washed with brine, dried over anhydrous sodium sulfate, filtered, and concentrated. The residue thus obtained in the previous step was purified by column chromatography on silica gel (MeOH : DCM = 1 : 19) to afford CN006-4 (240 mg, 70%). ^{1}H NMR (400 MHz, $CDCl_3$) δ 7.84 (s, 1H), 6.84 (m, 1H), 5.61 (m, 1H), 5.41 (m, 1H), 4.94 (m, 1H), 4.90 (s, 1H), 4.64 (d, J = 5.6 Hz, 2H), 4.36 (t, J = 6.8 Hz, 2H), 3.82 (m, 1H), 3.76-3.64 (m, 5H), 3.42 (m, 4H), 3.39–3.30 (m, 2H), 3.16–3.08 (m,

3H), 2.83 (m, 1H), 2.80–2.70 (m, 2H), 2.58–2.36 (m, 4H), 2.08–2.02 (m, 16H), 1.70 (m, 1H), 1.44 (s, 9H), 1.36 (m, 2H).

To a solution of CN006-4 (240 mg, 0.3 mmol) in MeOH/DCM (5 mL/5 mL) was added a solution of 1N NaOMe/MeOH (1.5 mL, 1.5 mmol). The reaction mixture was stirred at 25 °C for 15 h and then concentrated. The residue thus obtained in the previous step was purified by column chromatography on silica gel (MeOH : DCM = 2 : 8) to afford CN006-5 (167 mg, 85%). ^{1}H NMR (300 MHz, CD_3OD) δ 7.84 (s, 1H), 4.58 (s, 2H), 4.39 (t, J = 6.9 Hz, 2H), 4.16–4.10 (m, 2H), 3.99–3.82 (m, 3H), 3.70 (m, 4H), 3.51–3.42 (m, 6H), 3.40 (m, 1H), 3.21 (m, 1H), 3.04 (m, 2H), 2.83 (m, 1H), 2.62 (m, 2H), 2.08–1.83 (m, 8H), 1.43 (s, 9H), 1.36 (m, 2H).

A solution of 2N HCl/diethyl ether (0.75 mL, 1.5 mmol) was added to a DCM solution of CN006-5 (167 mg, 0.3 mmol). The reaction mixture was stirred at 25 °C for 15 h and then concentrated to afford a hydrochloride salt of CN006 (150 mg, 93%).

Preparation of CN007

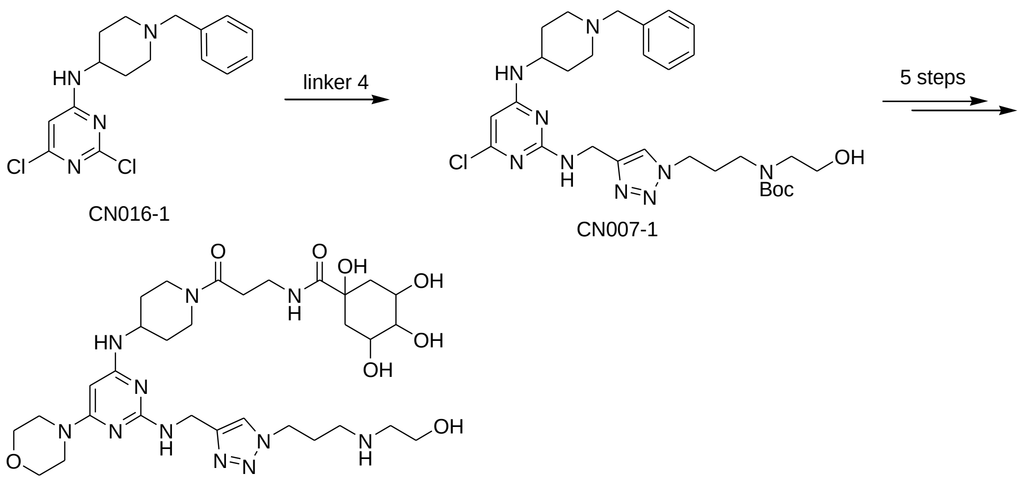

CN007 was prepared from CN016-1 following a synthetic procedure similar to that used for CN006 and obtained in 28% yield over six steps.

Preparation of CN008

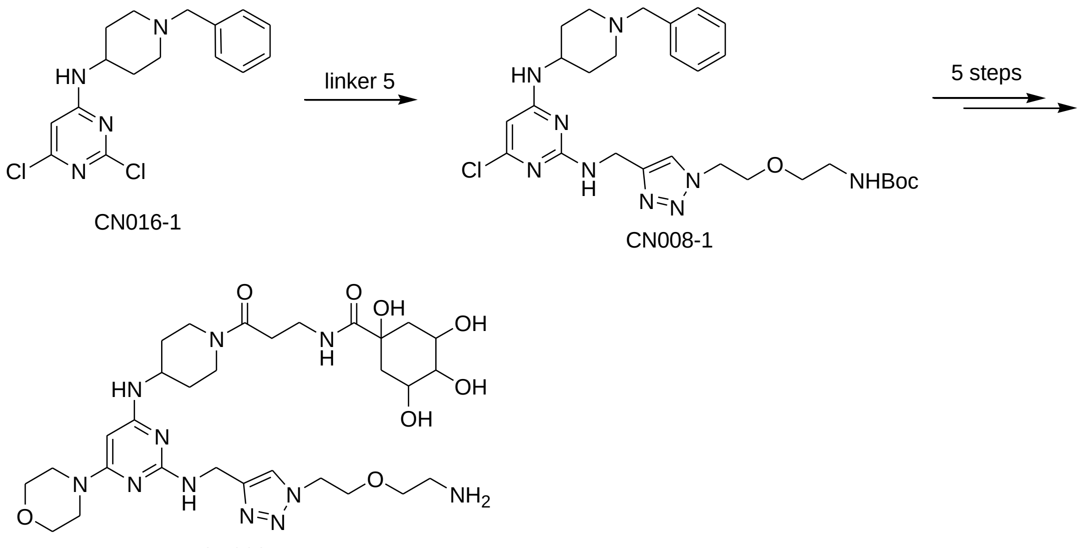

CN008 was prepared from CN016-1 following a synthetic procedure similar to that used for CN006 and obtained in 22% yield over six steps.

Preparation of CN009

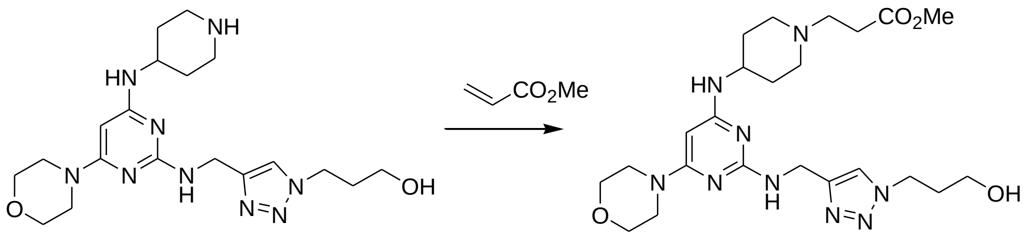

CN016-3 CN009

A solution of CN016-3 (160 mg, 0.4 mmol), methyl acrylate (70 mg, 0.8 mmol) and TEA (60 mg, 0.6 mmol) in MeOH (6 mL) was stirred at 25 °C for 16 h and then concentrated. The residue thus obtained in the previous step was purified by column chromatography on silica gel (MeOH : ethyl acetate = 1 : 4) to give CN009 (129 mg, 67%).

Preparation of CN010

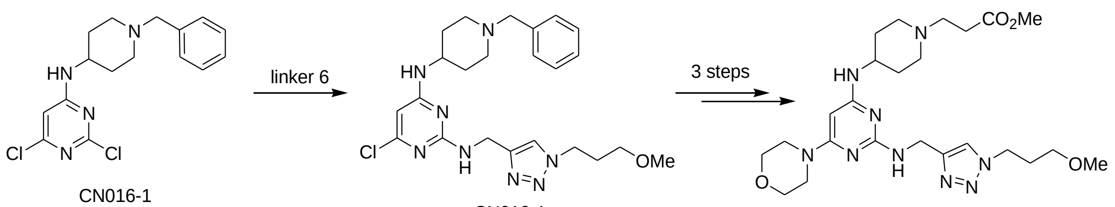

CN016-1 CN010-1 CN010

CN010 was prepared from CN016-1 following a synthetic procedure similar to that used for CN009 and obtained in 32% yield over four steps.

Preparation of CN011

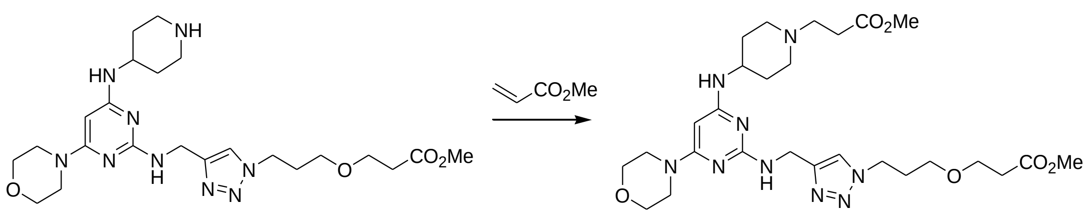

CN005-1 CN011

CN011 was prepared from CN005-1 following a synthetic procedure similar to that

used for CN009 and obtained in 69% yield.

Preparation of CN013

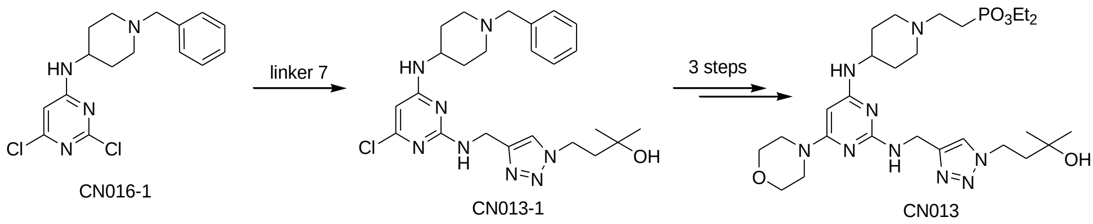

CN013 was prepared from CN016-1 following a synthetic procedure similar to that used for CN012 and obtained in 32% yield over four steps.

Preparation of CN014

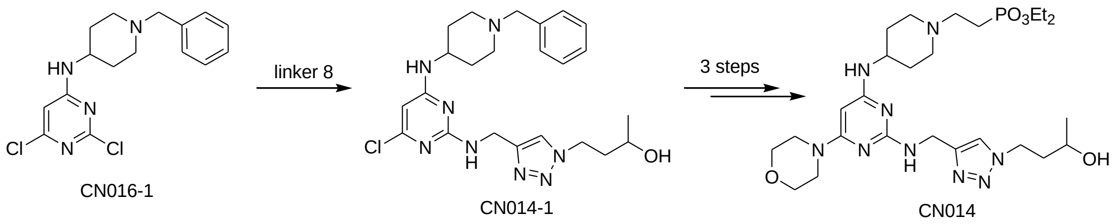

CN014 was prepared from CN016-1 following a synthetic procedure similar to that used for CN012 and obtained in 30% yield over four steps.

Preparation of CN015

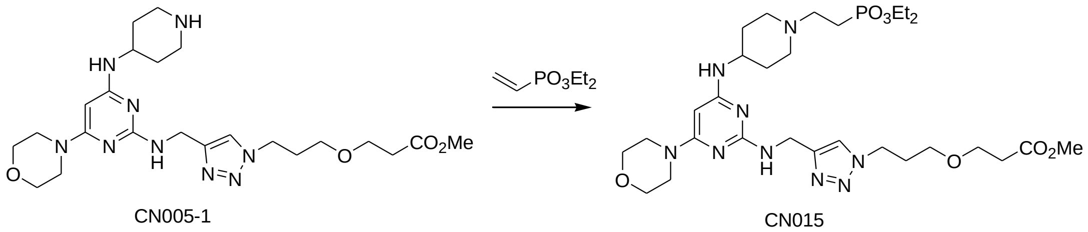

CN015 was prepared from CN005-1 following a synthetic procedure similar to that used for CN012 and obtained in 56% yield.

Preparation of CN017

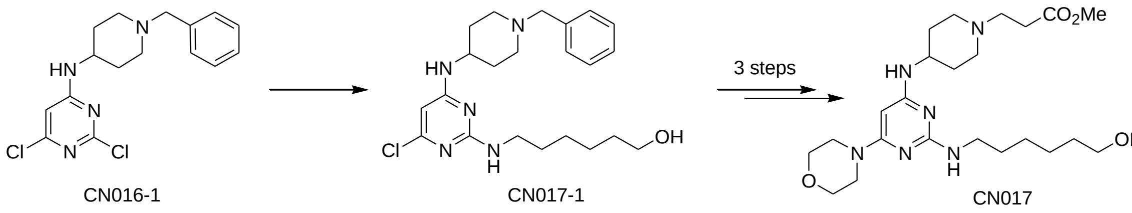

CN017 was prepared from CN016-1 following a synthetic procedure similar to that used for CN009 and obtained in 29% yield over four steps.

Preparation of CN018

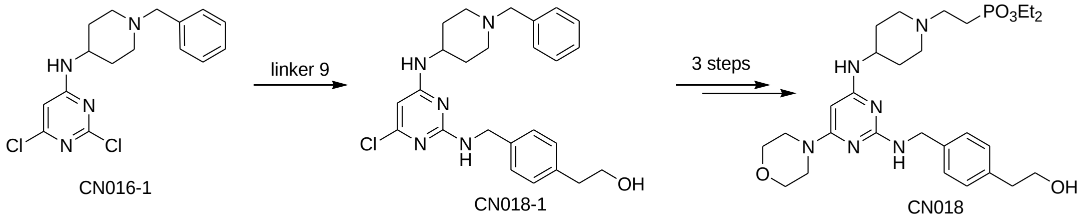

CN018 was prepared from CN016-1 following a synthetic procedure similar to that used for CN012 and obtained in 31% yield over four steps.

Preparation of CN019

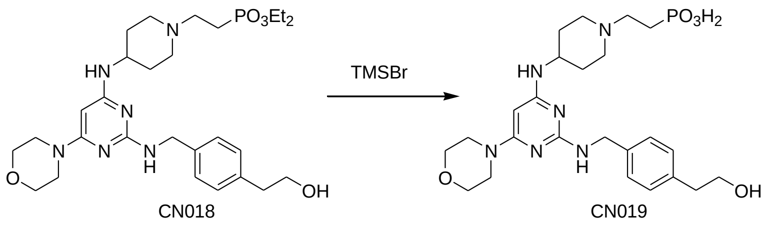

CN019 was prepared from CN018 following a synthetic procedure similar to that used for CN016 and obtained in 85% yield.

Preparation of CN020

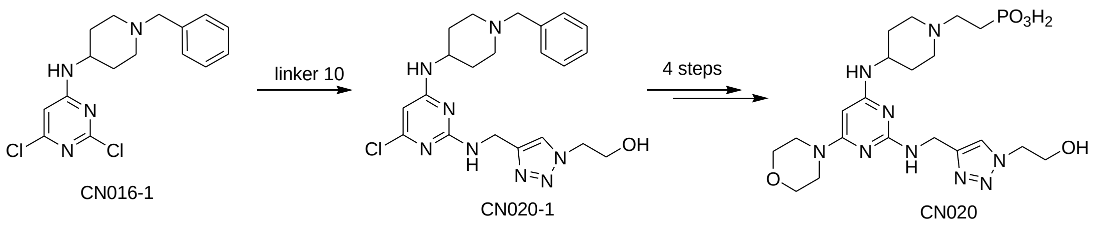

CN014 was prepared from CN016-1 following a synthetic procedure similar to that used for CN012 and obtained in 28% yield over five steps.

Preparation of CN021

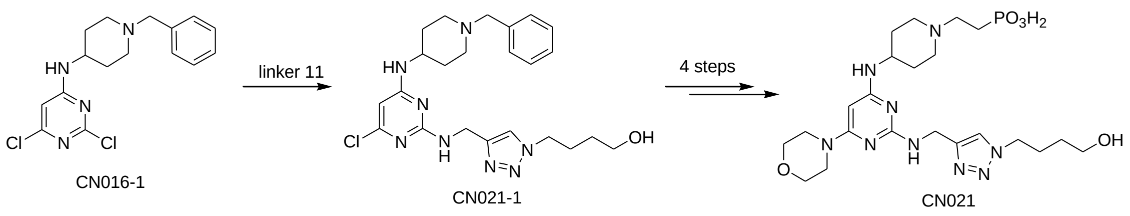

CN014 was prepared from CN016-1 following a synthetic procedure similar to that used for CN012 and obtained in 29% yield over five steps.

Preparation of CN025

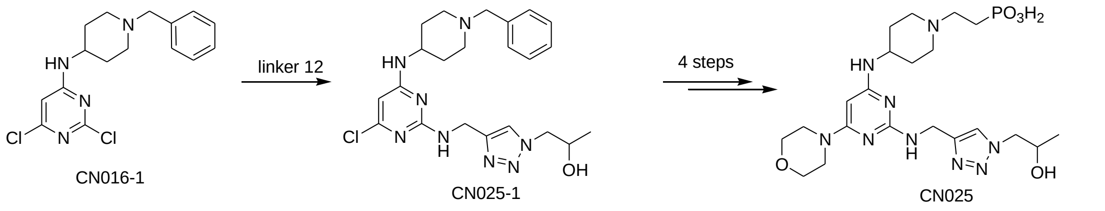

CN014 was prepared from CN016-1 following a synthetic procedure similar to that used for CN012 and obtained in 28% yield over five steps.

^{1}H-NMR of CN004

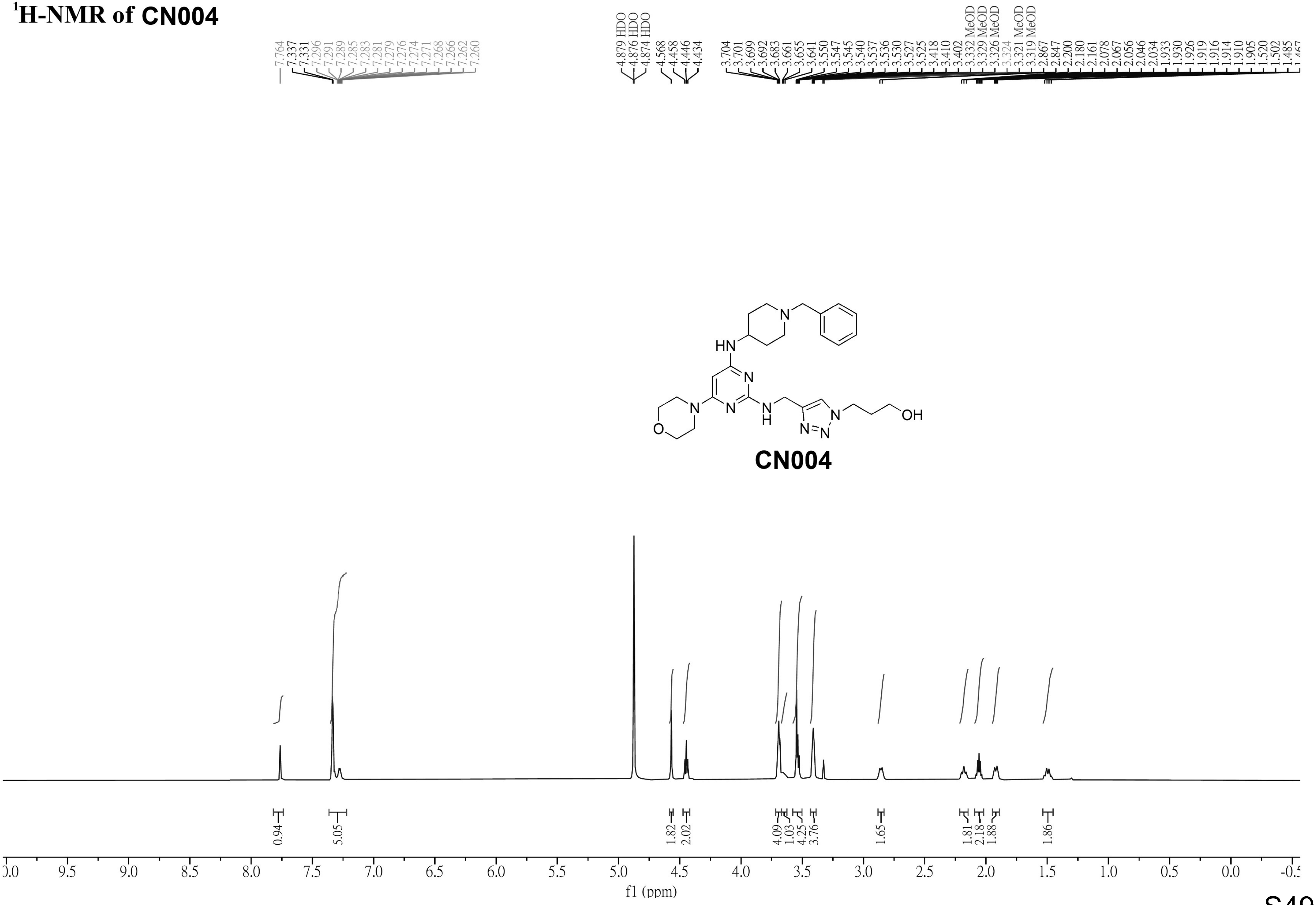

CN004

^{13}C-NMR of CN004

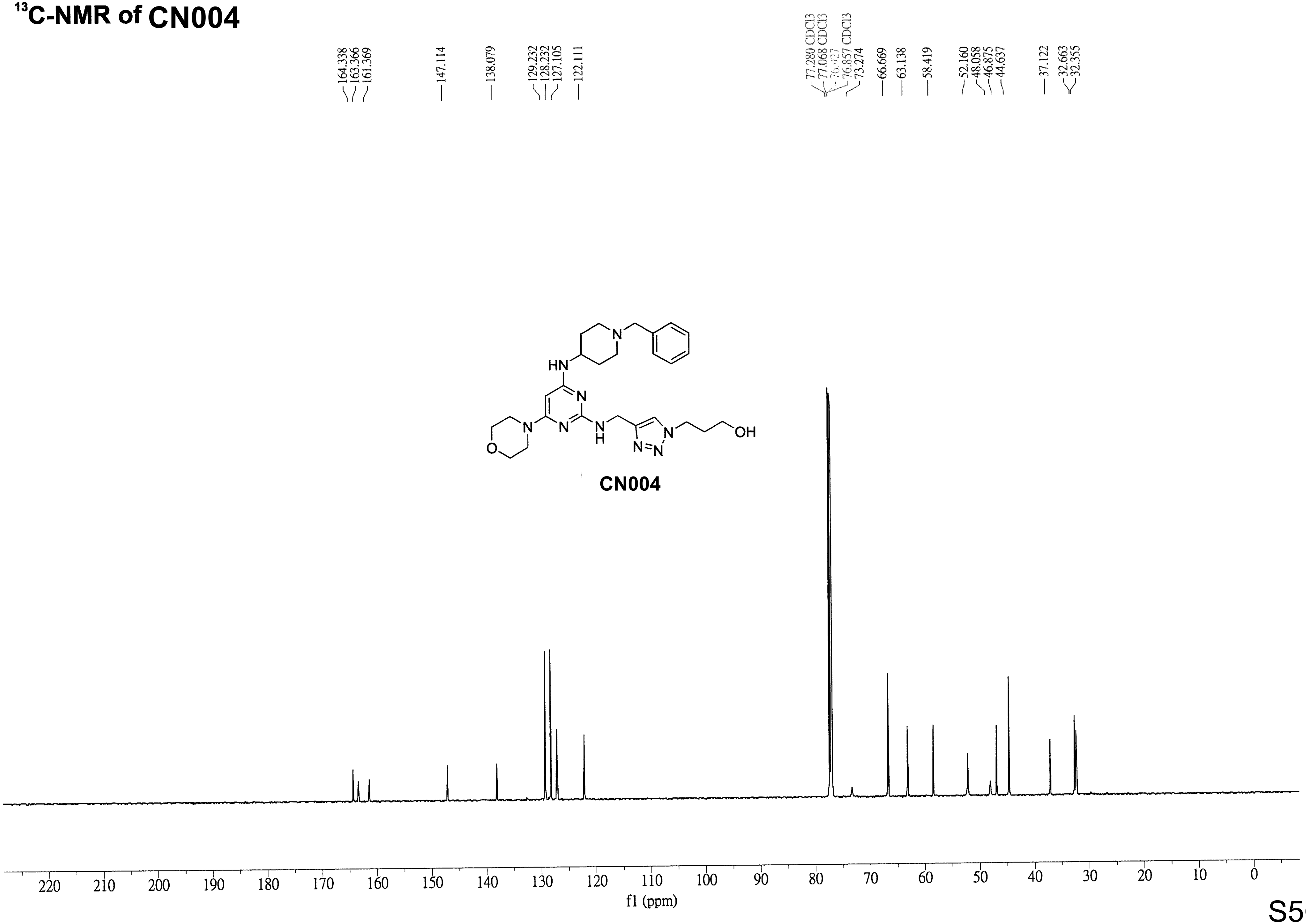

^{1}H-NMR of CN012

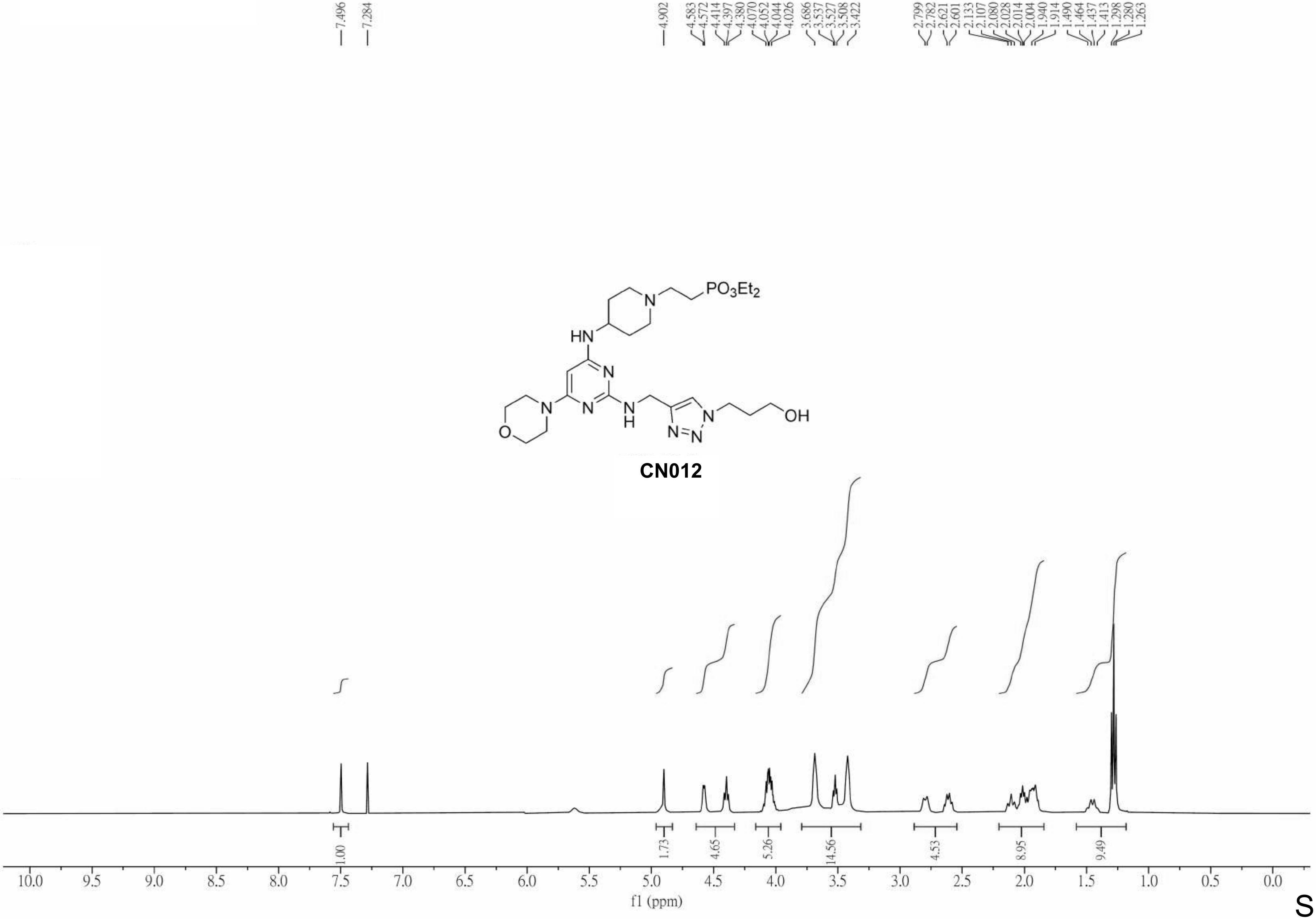

CN012

^{13}C-NMR of CN012

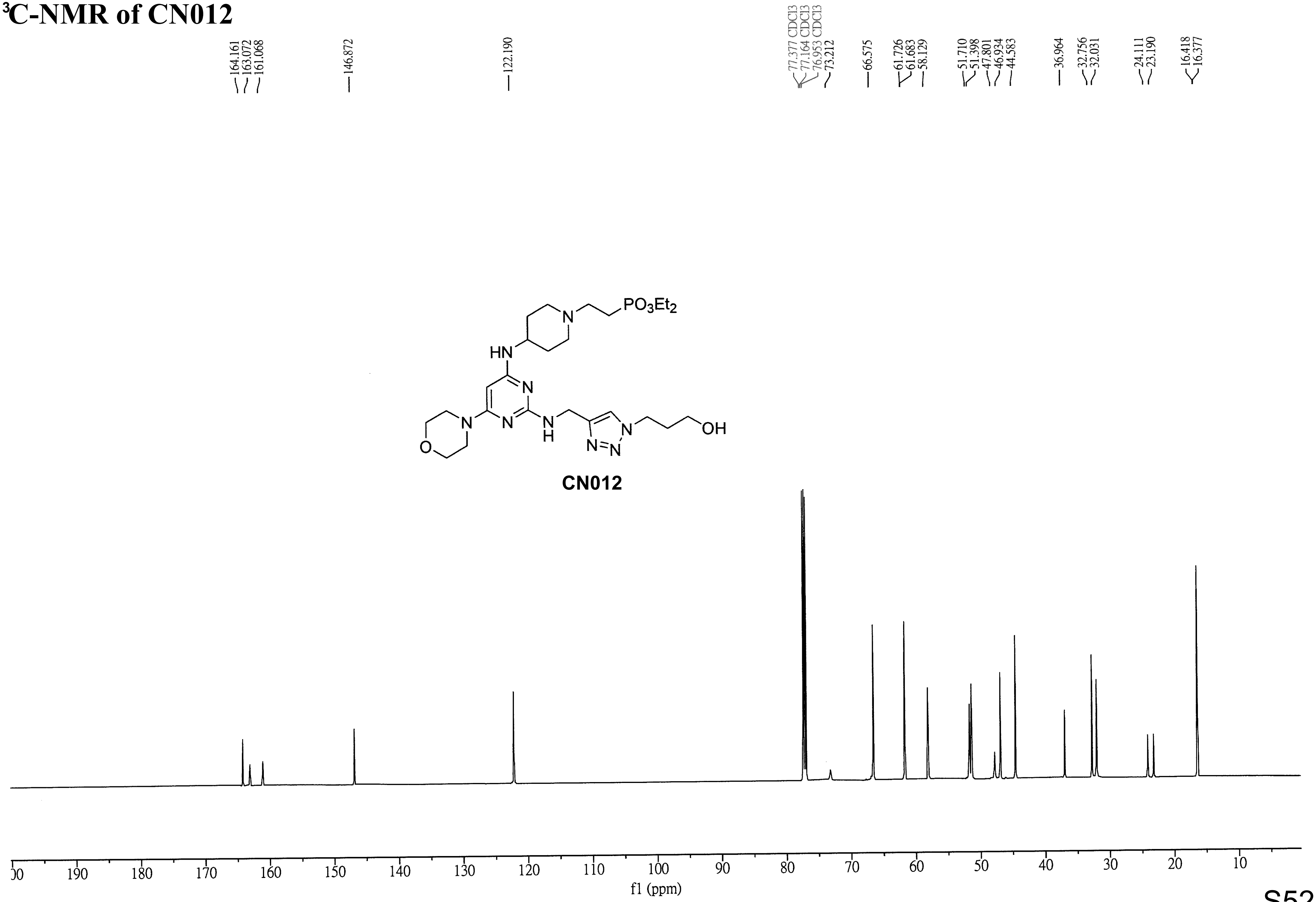

^{1}H-NMR of CN016

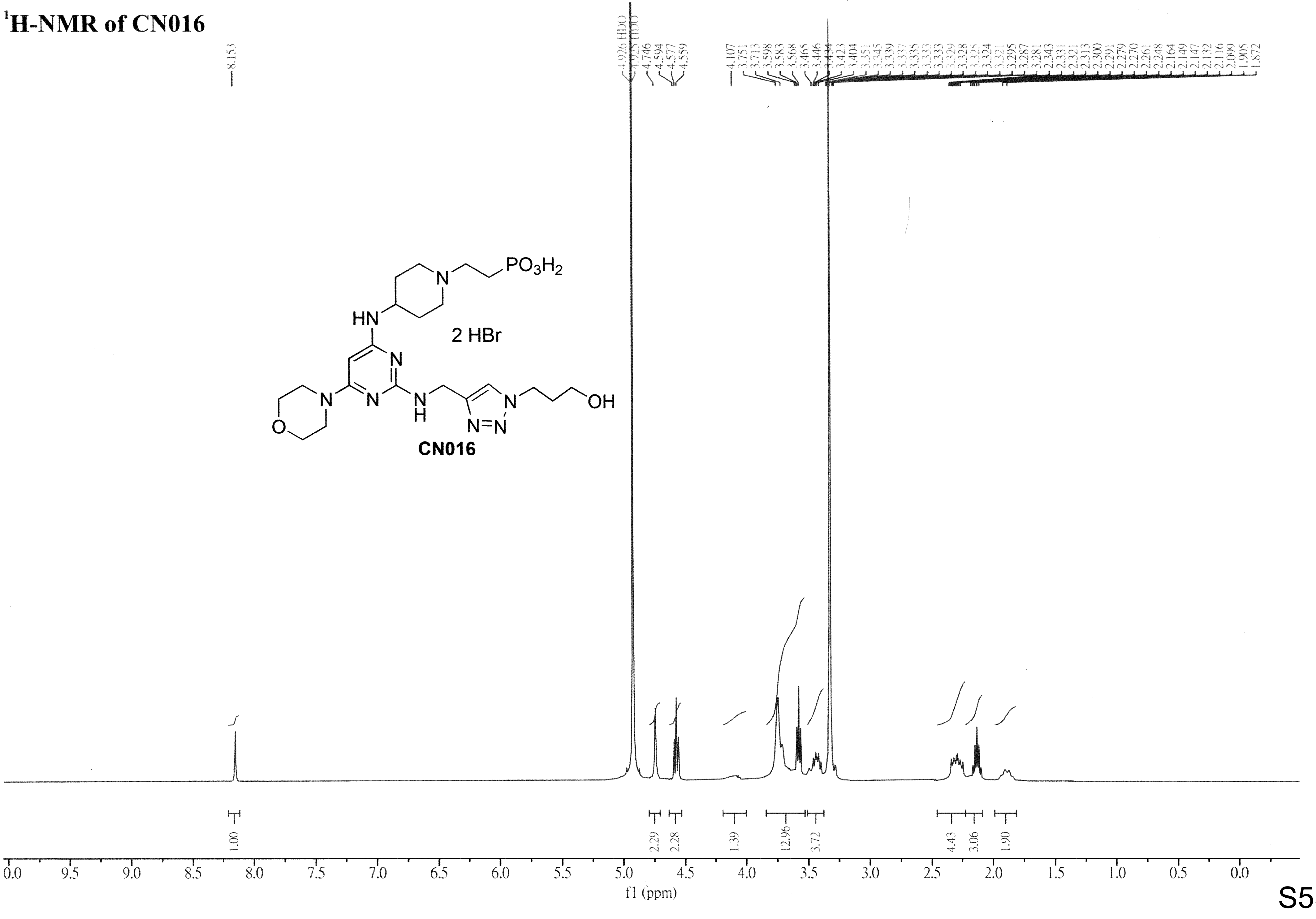

^{13}C-NMR of CN016

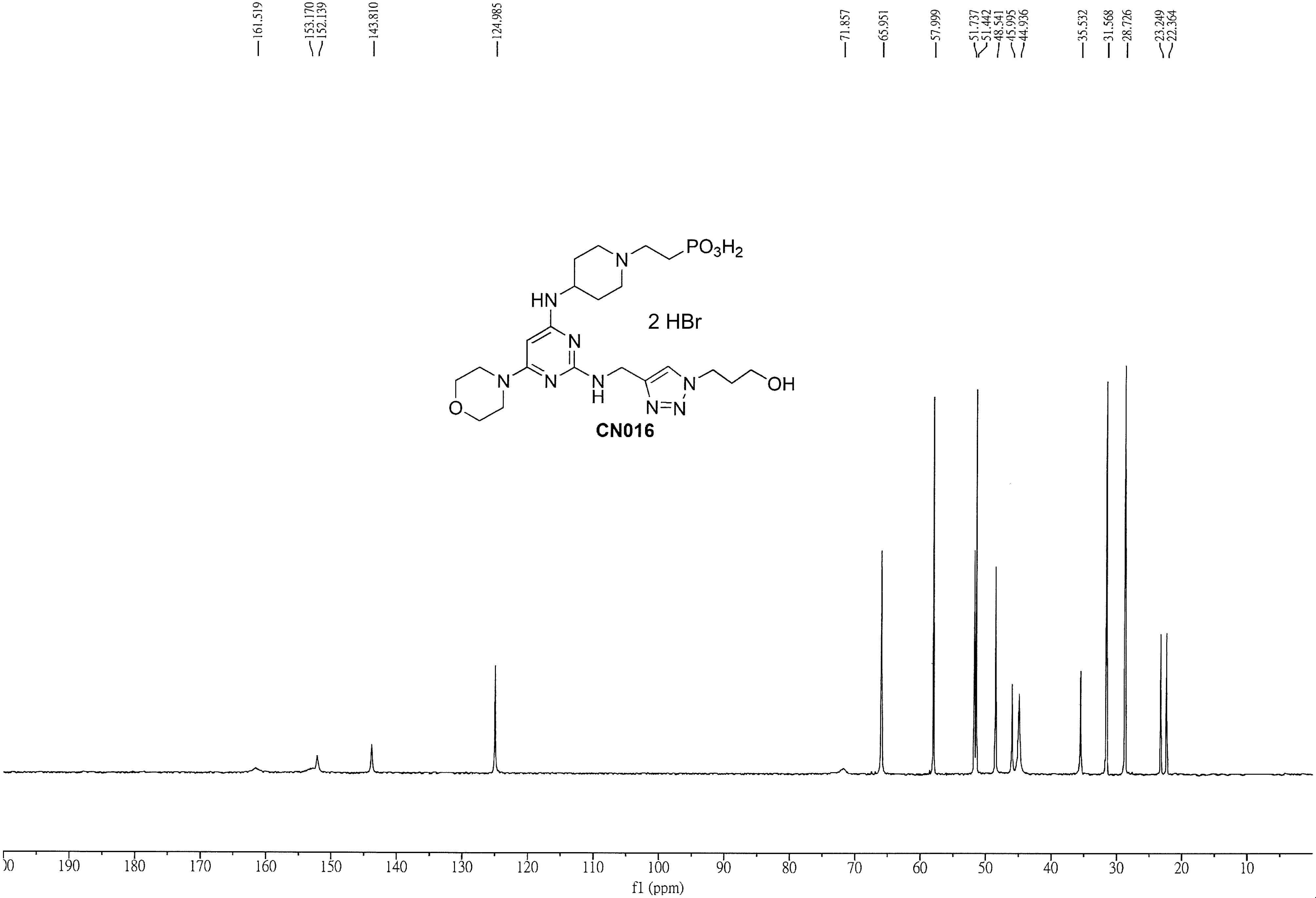

^{1}H-NMR of CN020

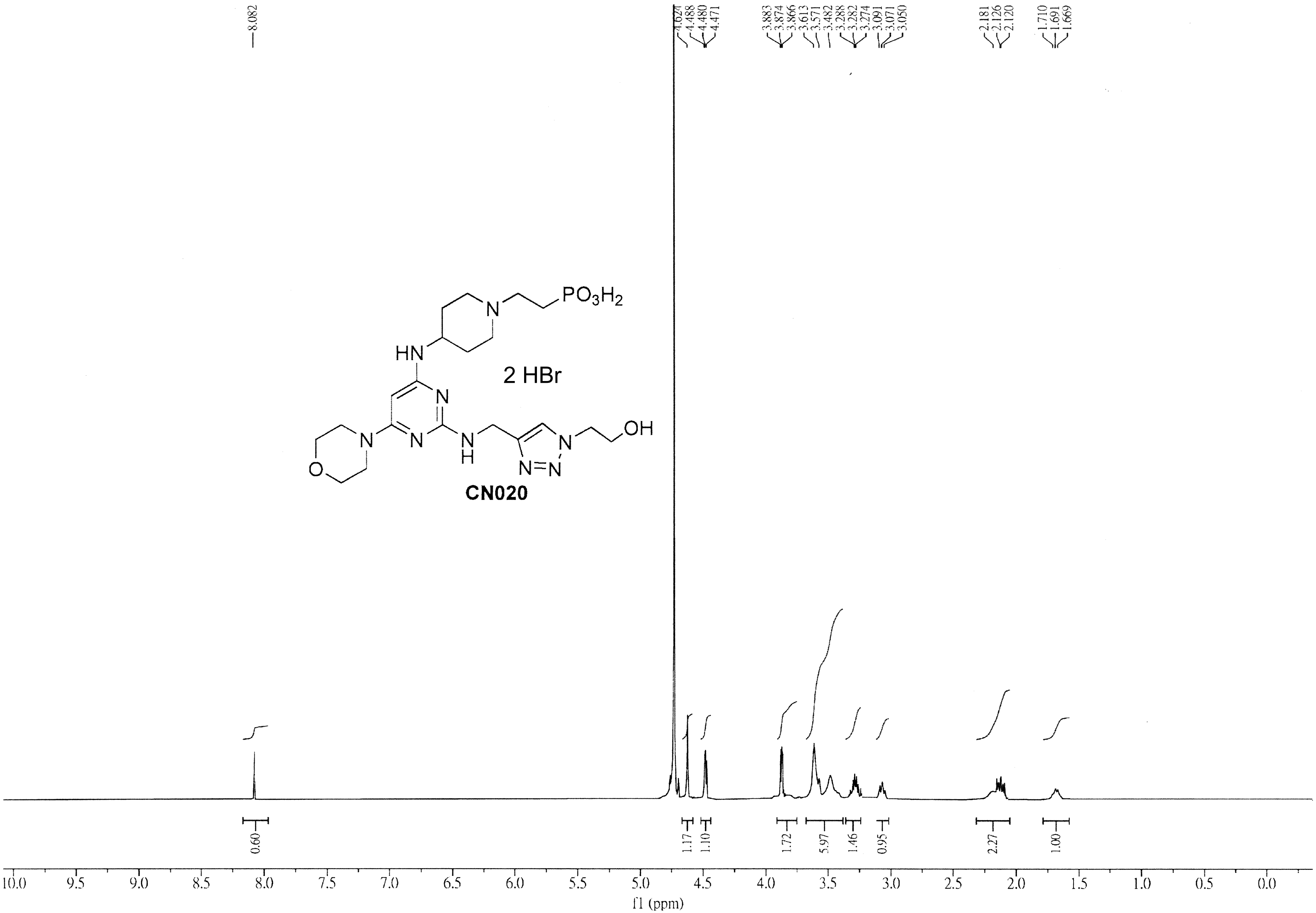

^{13}C-NMR of CN020

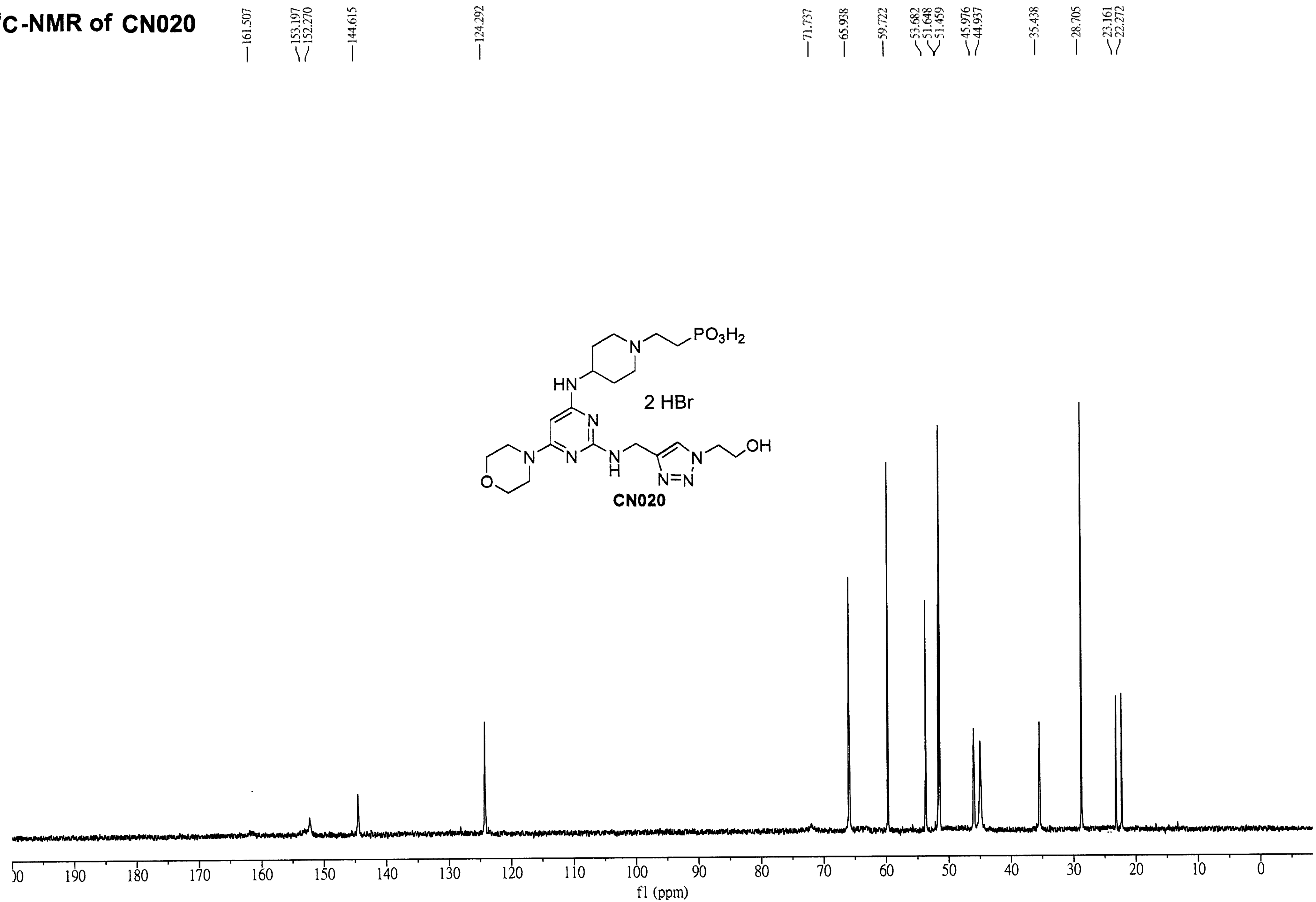

^{1}H-NMR of CN021

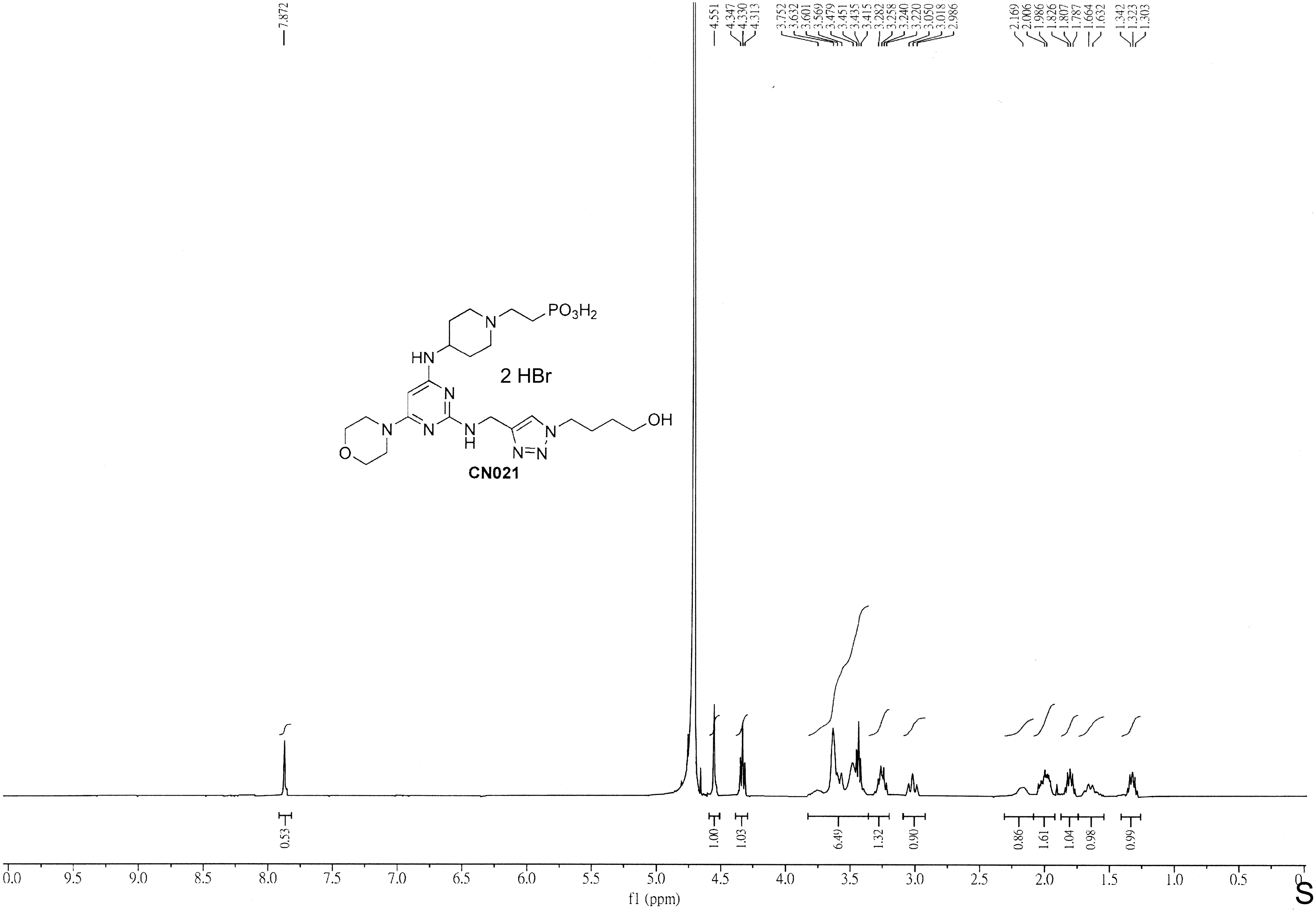

^{13}C-NMR of CN021

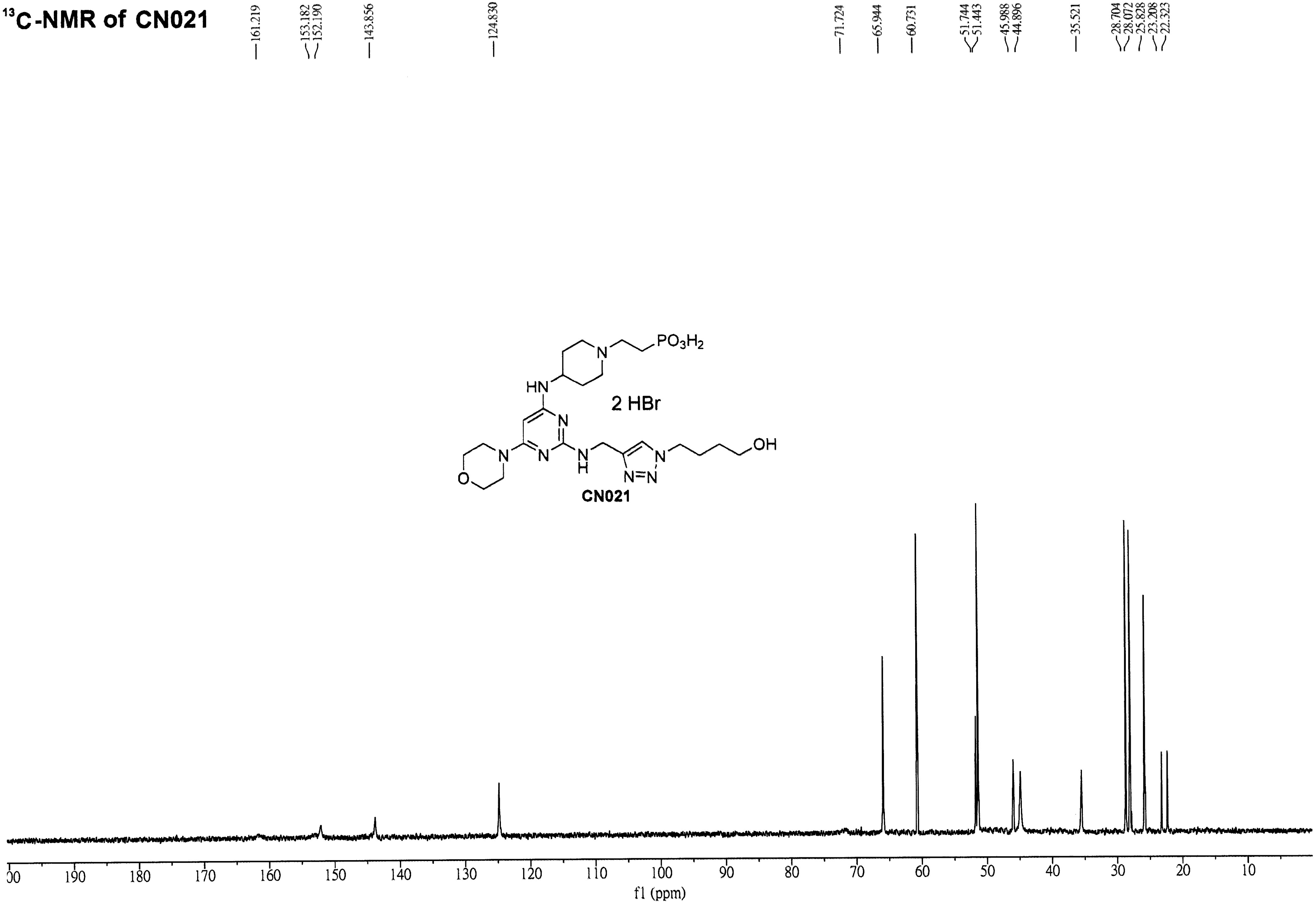

^{1}H-NMR of CN022

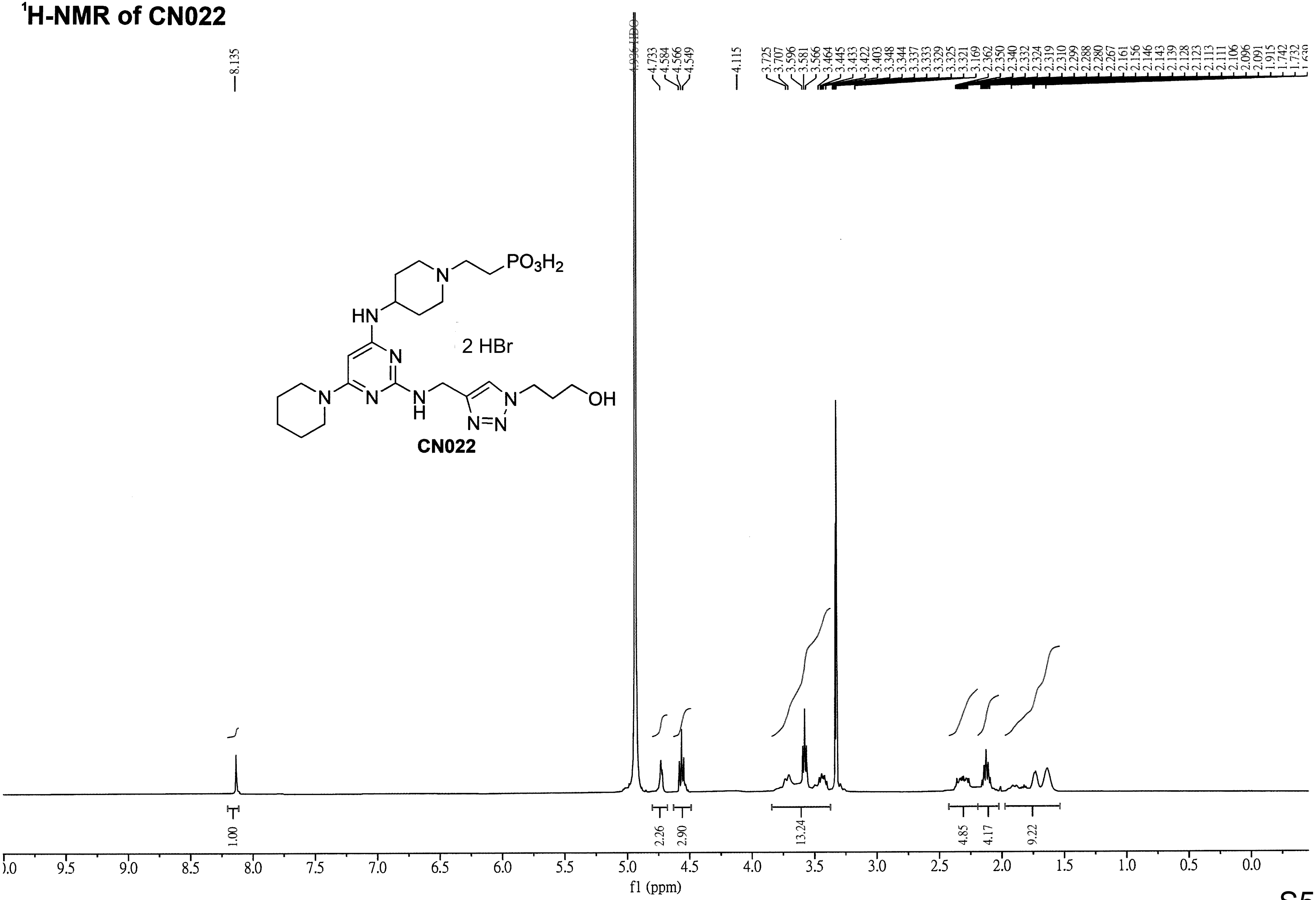

^{13}C-NMR of CN022

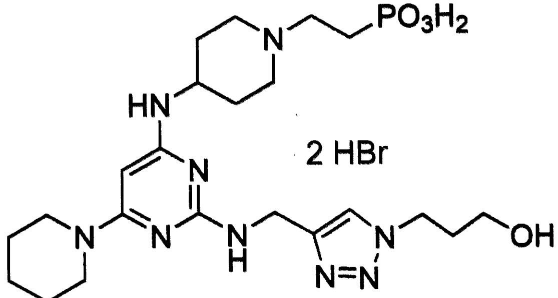

CN022

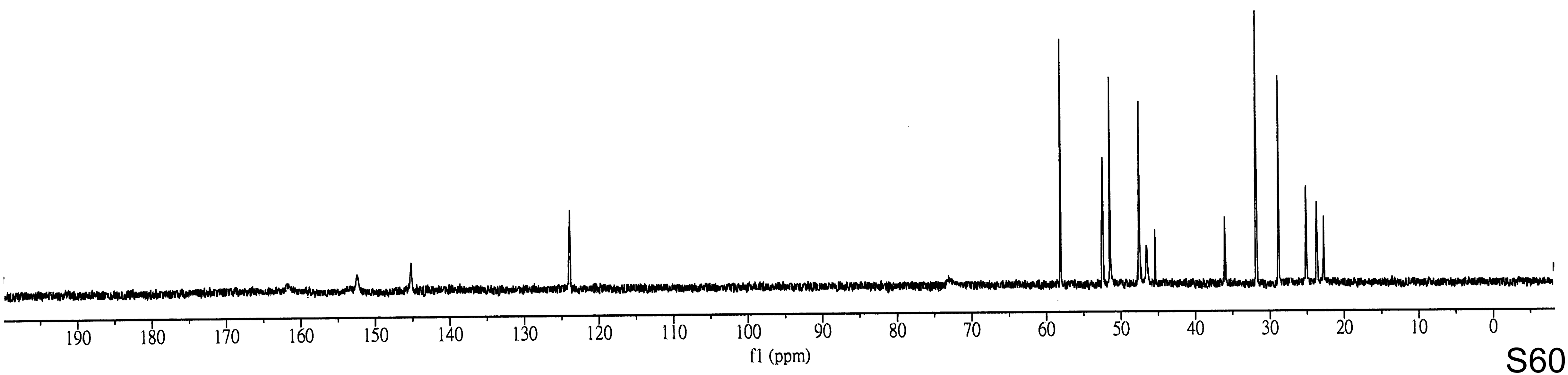

^{1}H-NMR of CN023

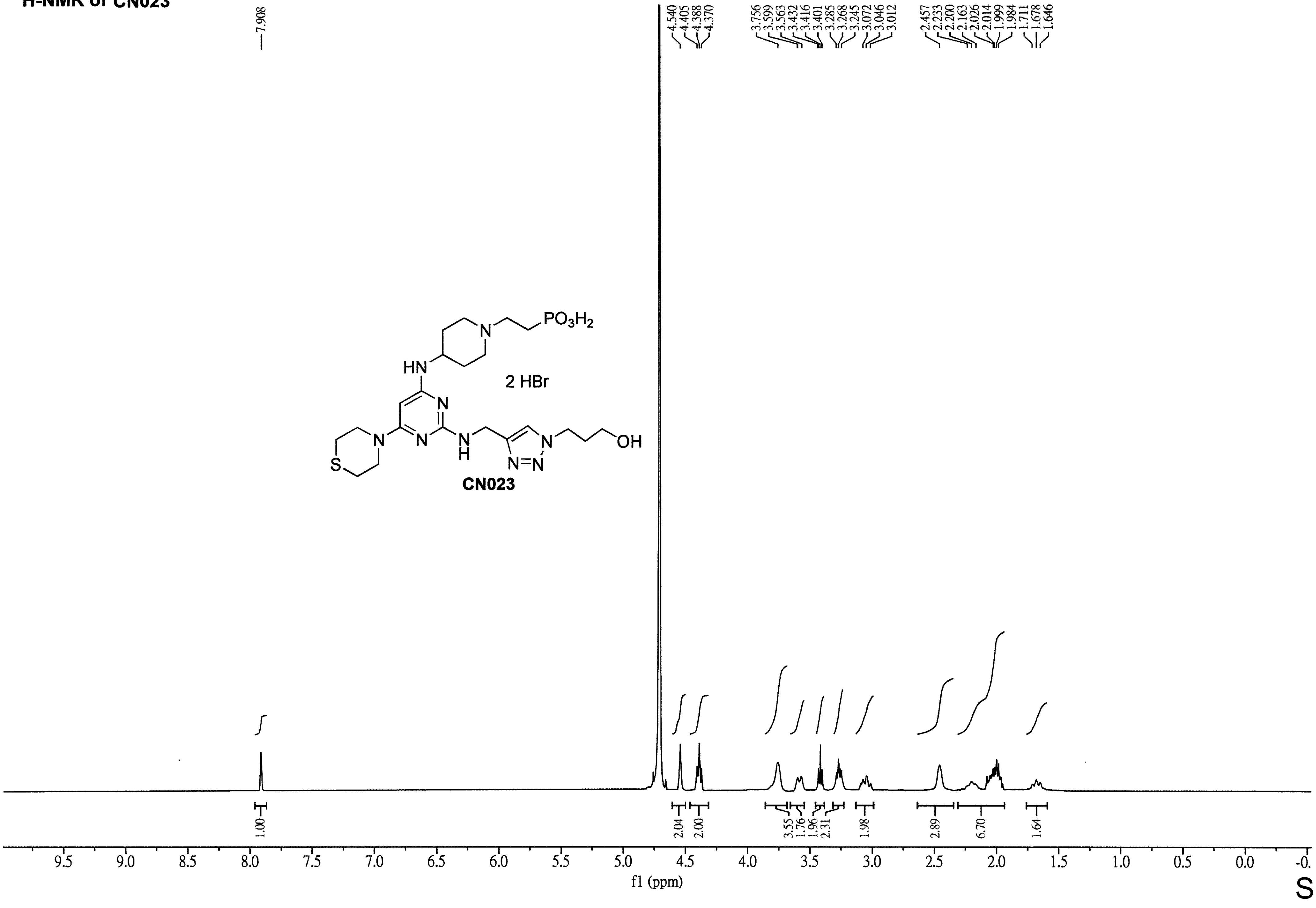

^{13}C-NMR of CN023

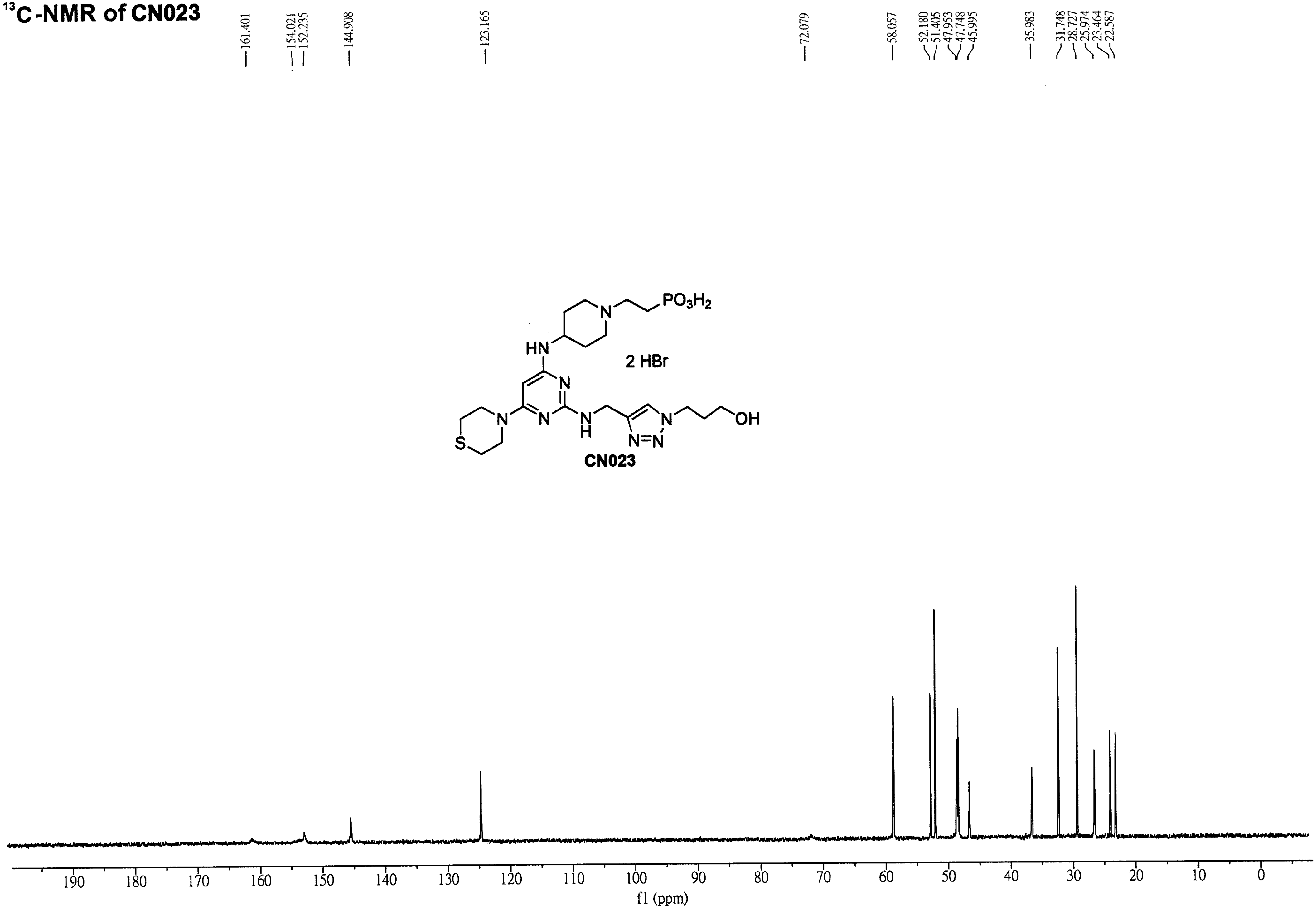

^{1}H-NMR of CN024

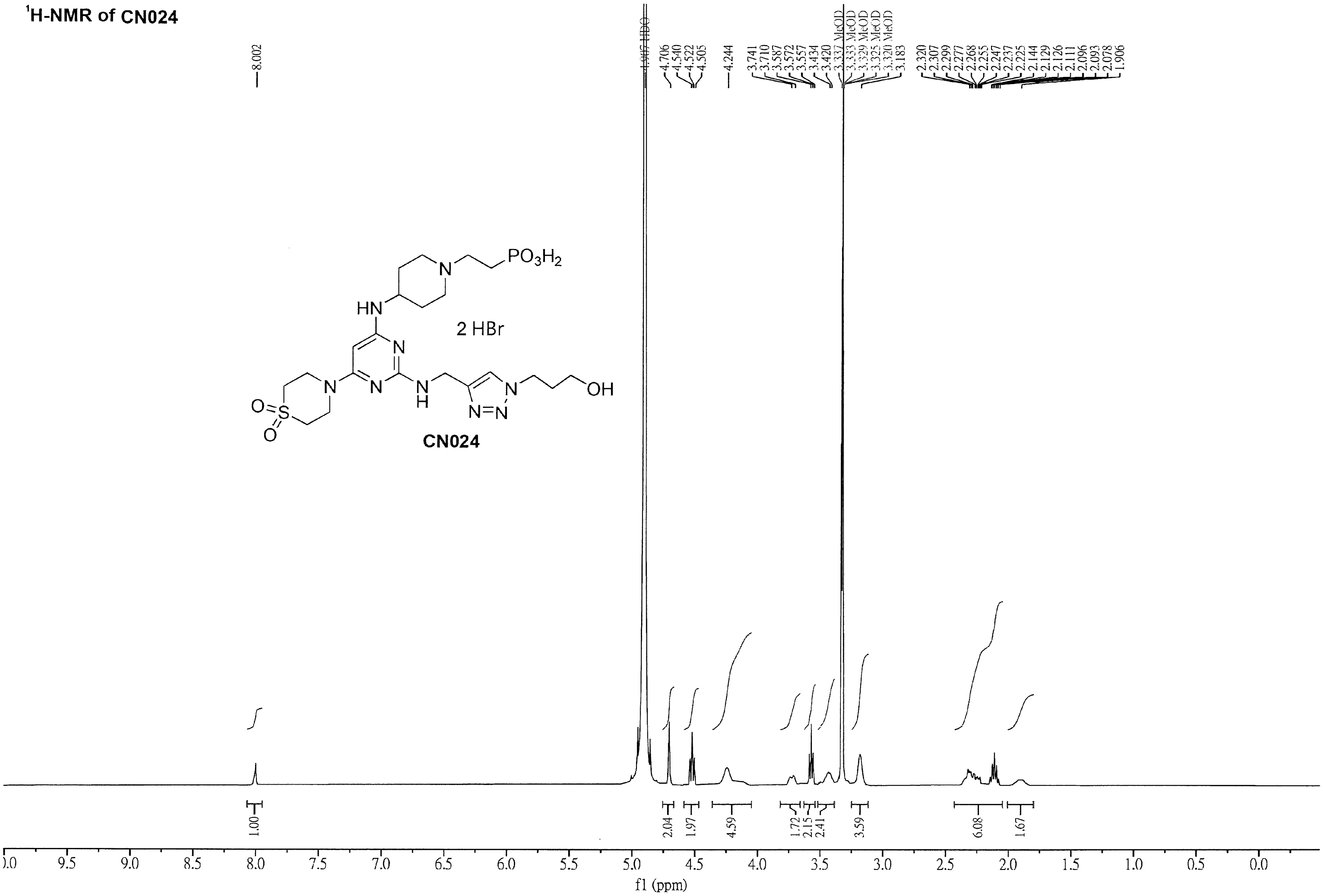

^{13}C-NMR of CN024

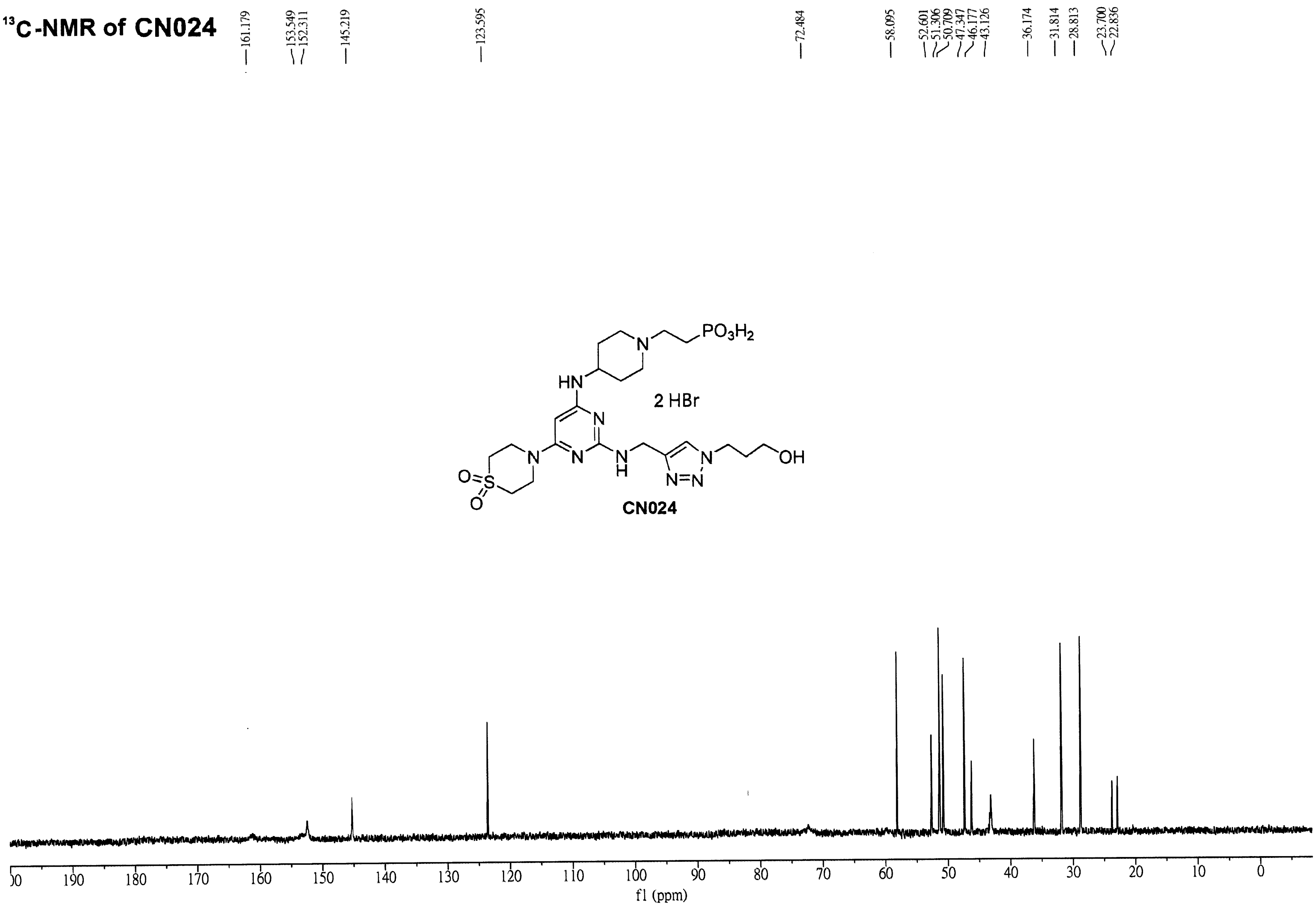

^{1}H-NMR of CN025

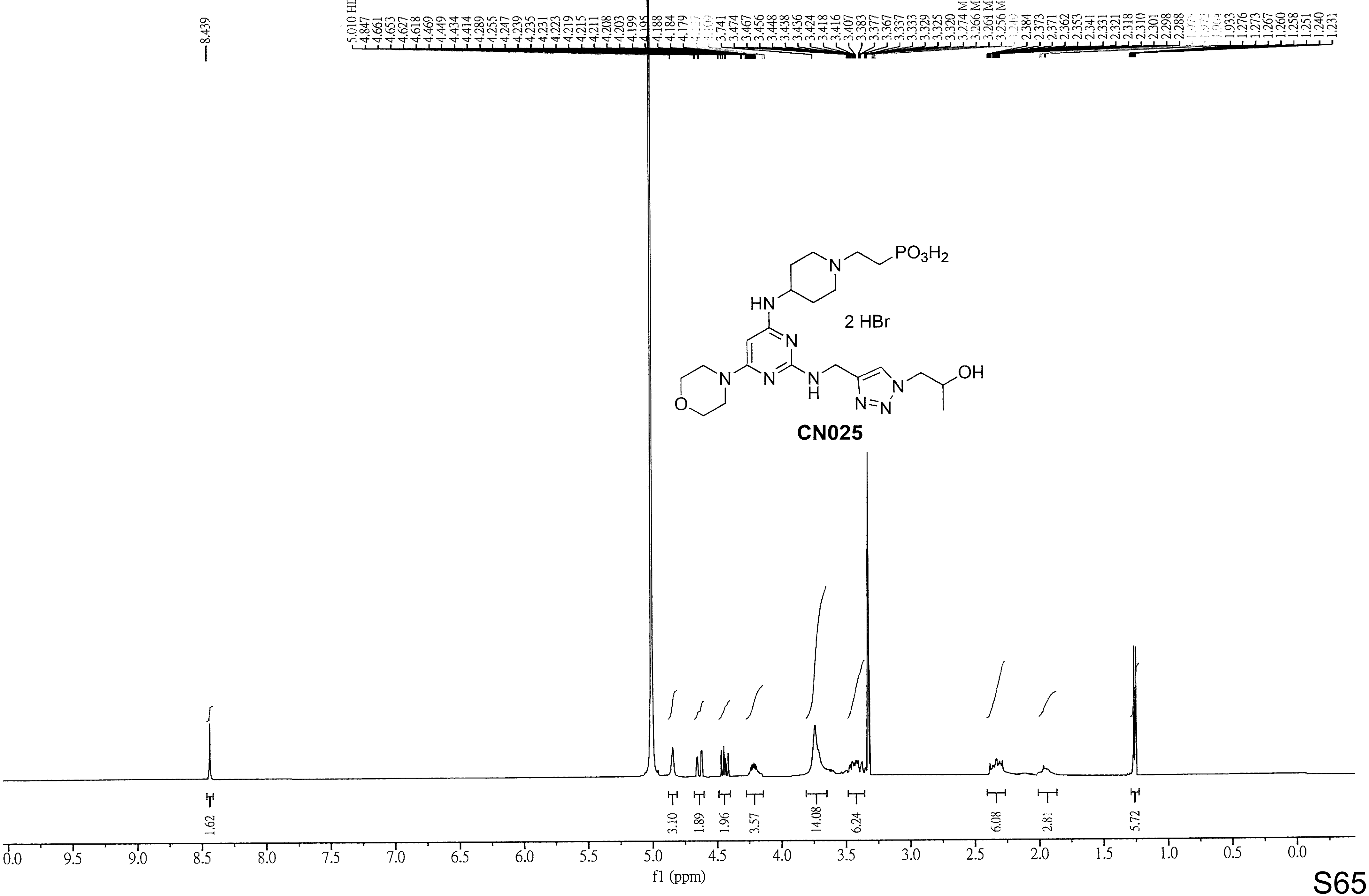

^{13}C-NMR of CN025

161.805, 154.708, 153.598, 144.604, 124.877, 72.332, 66.054, 57.032, 52.086, 51.436, 48.883, 46.002, 44.921, 35.841, 28.725, 23.418, 22.541, 19.116

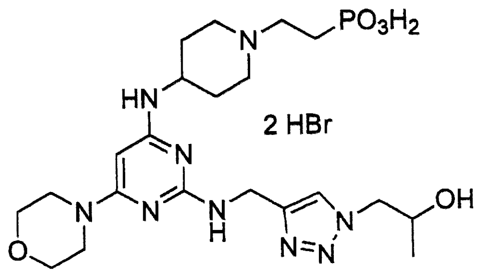

CN025

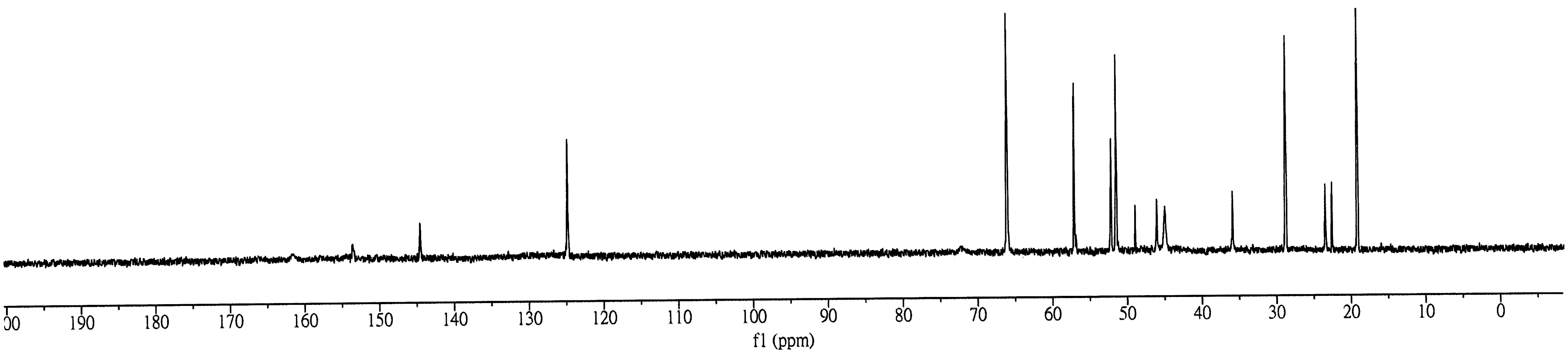

^{1}H-NMR of CN026

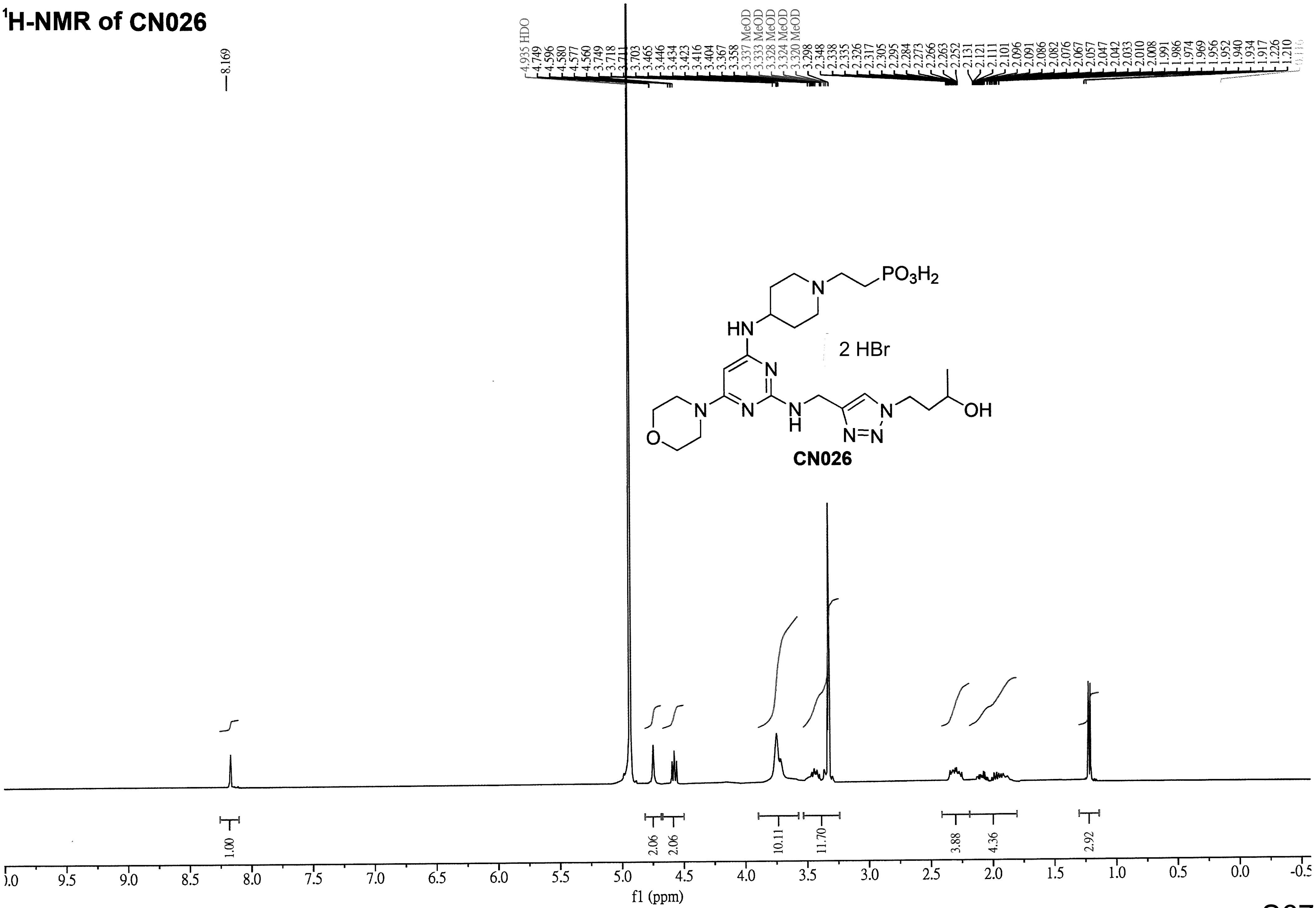

^{13}C-NMR of CN026

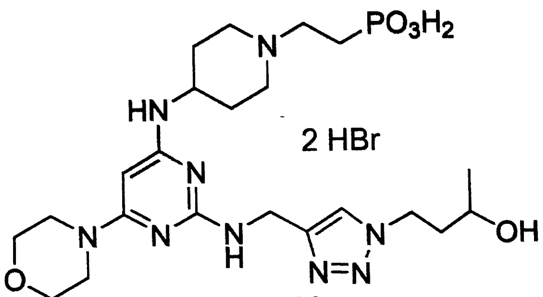

CN026

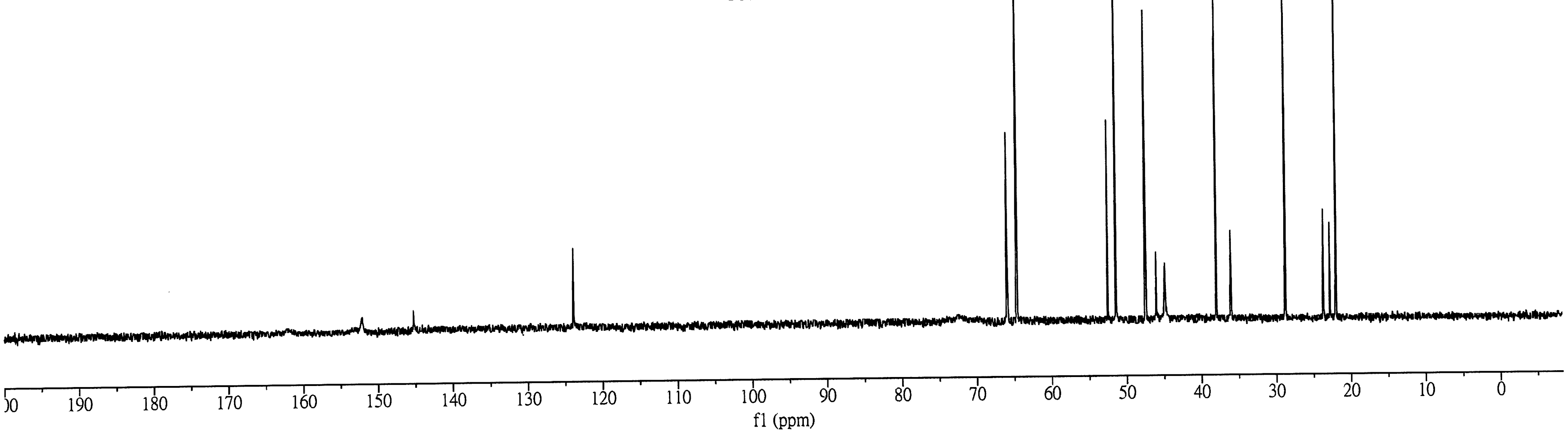

¹H-NMR of CN027

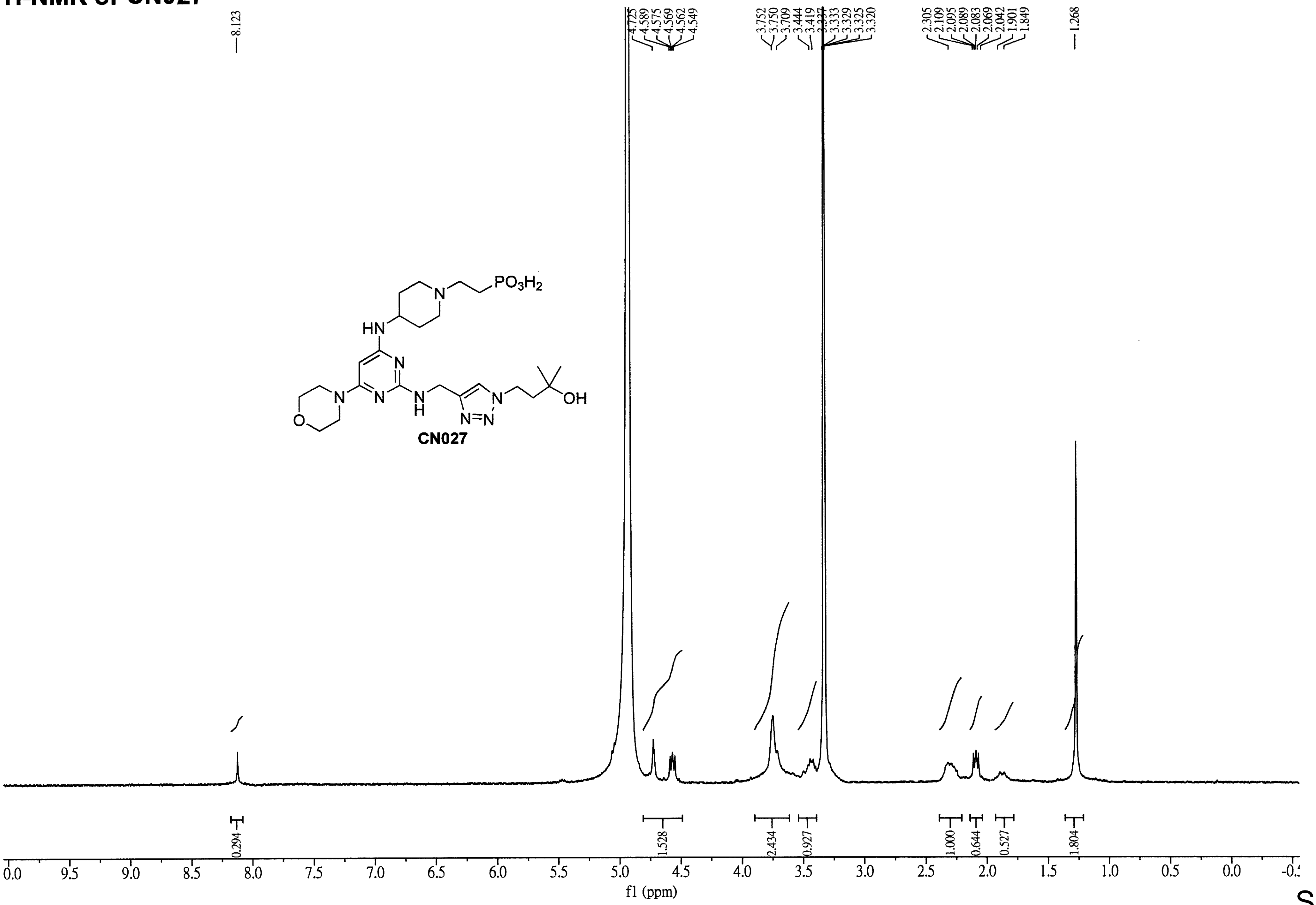

^{13}C-NMR of CN027

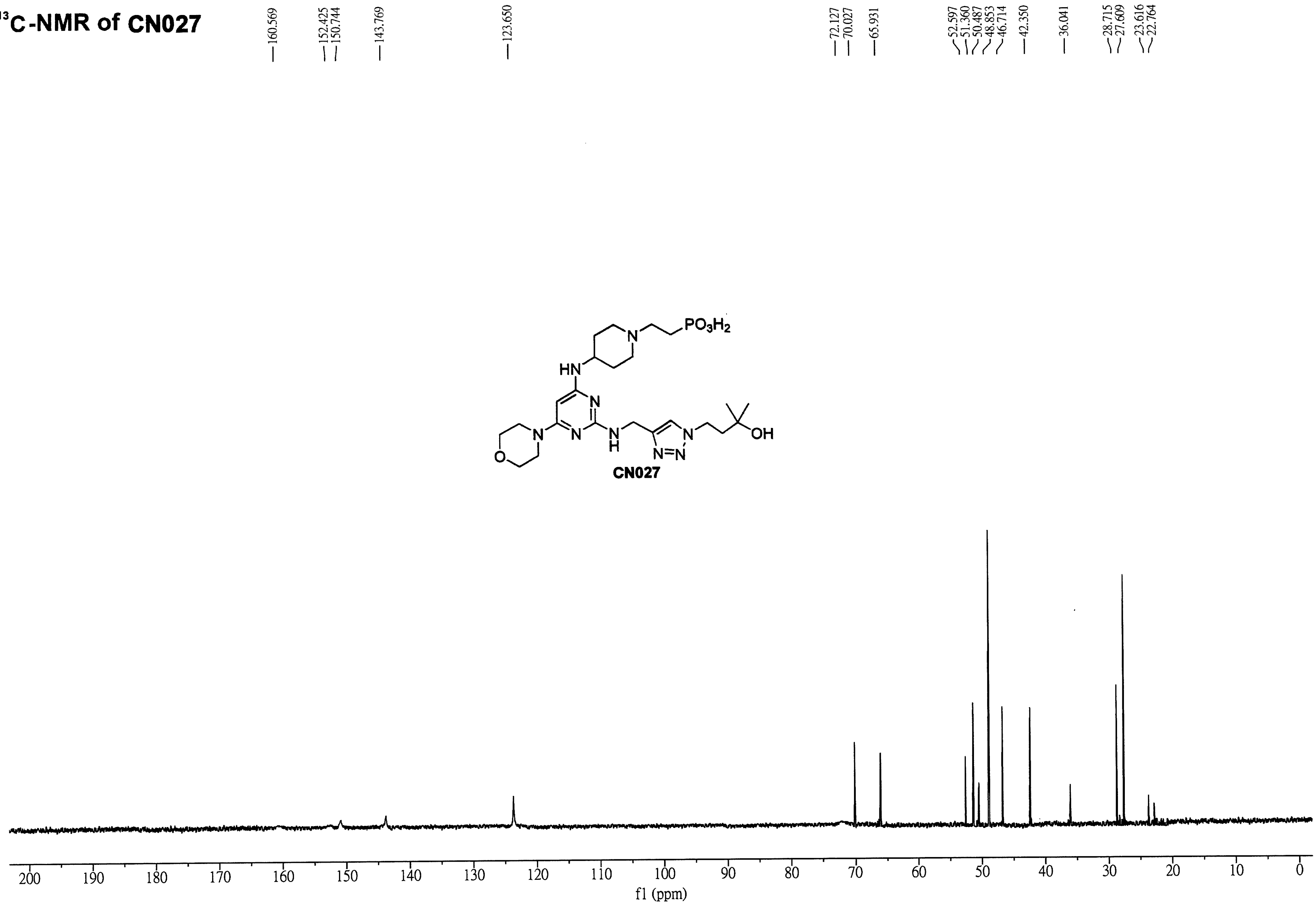

^{1}H-NMR of CN028

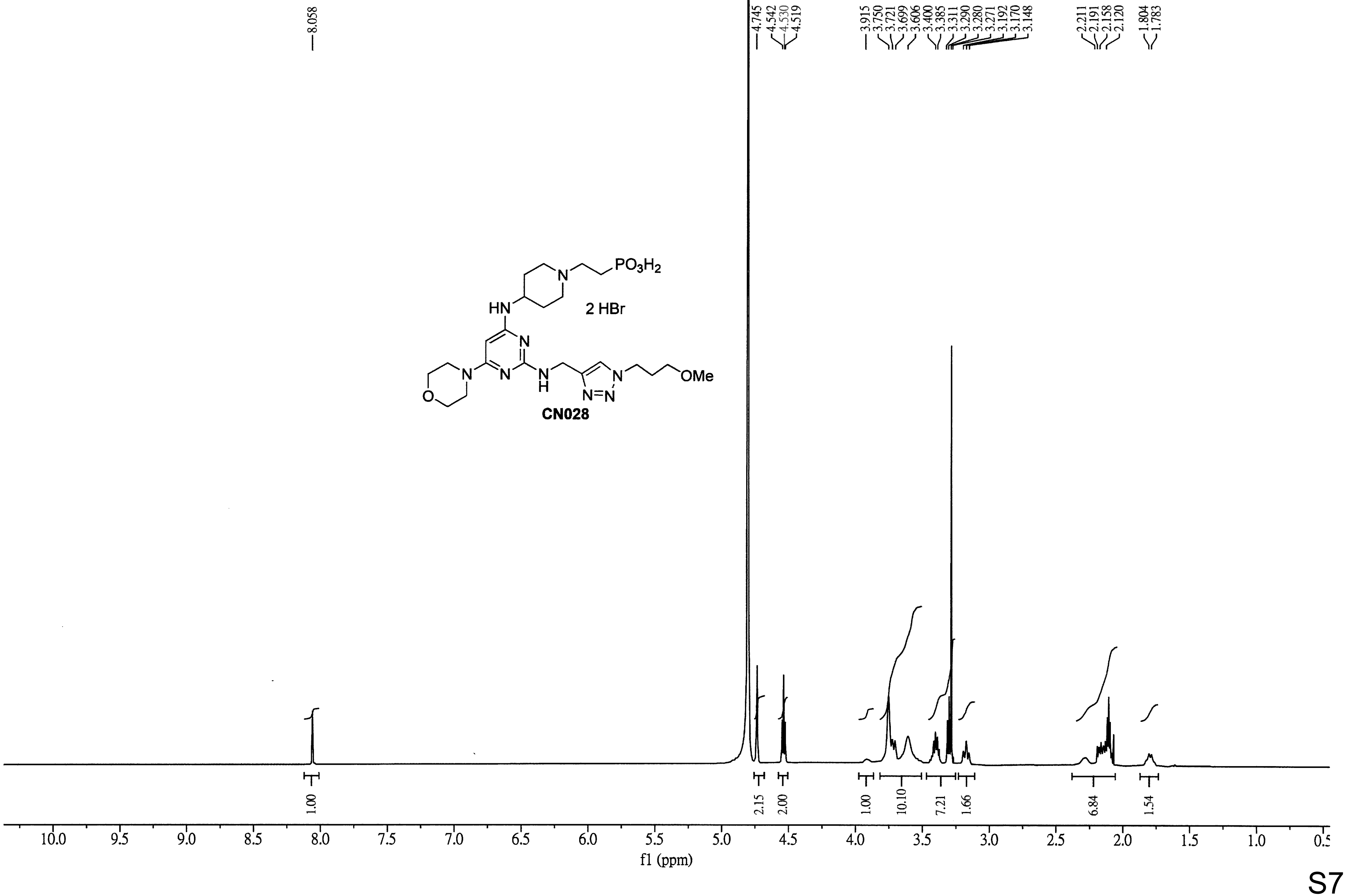

^{13}C-NMR of CN028

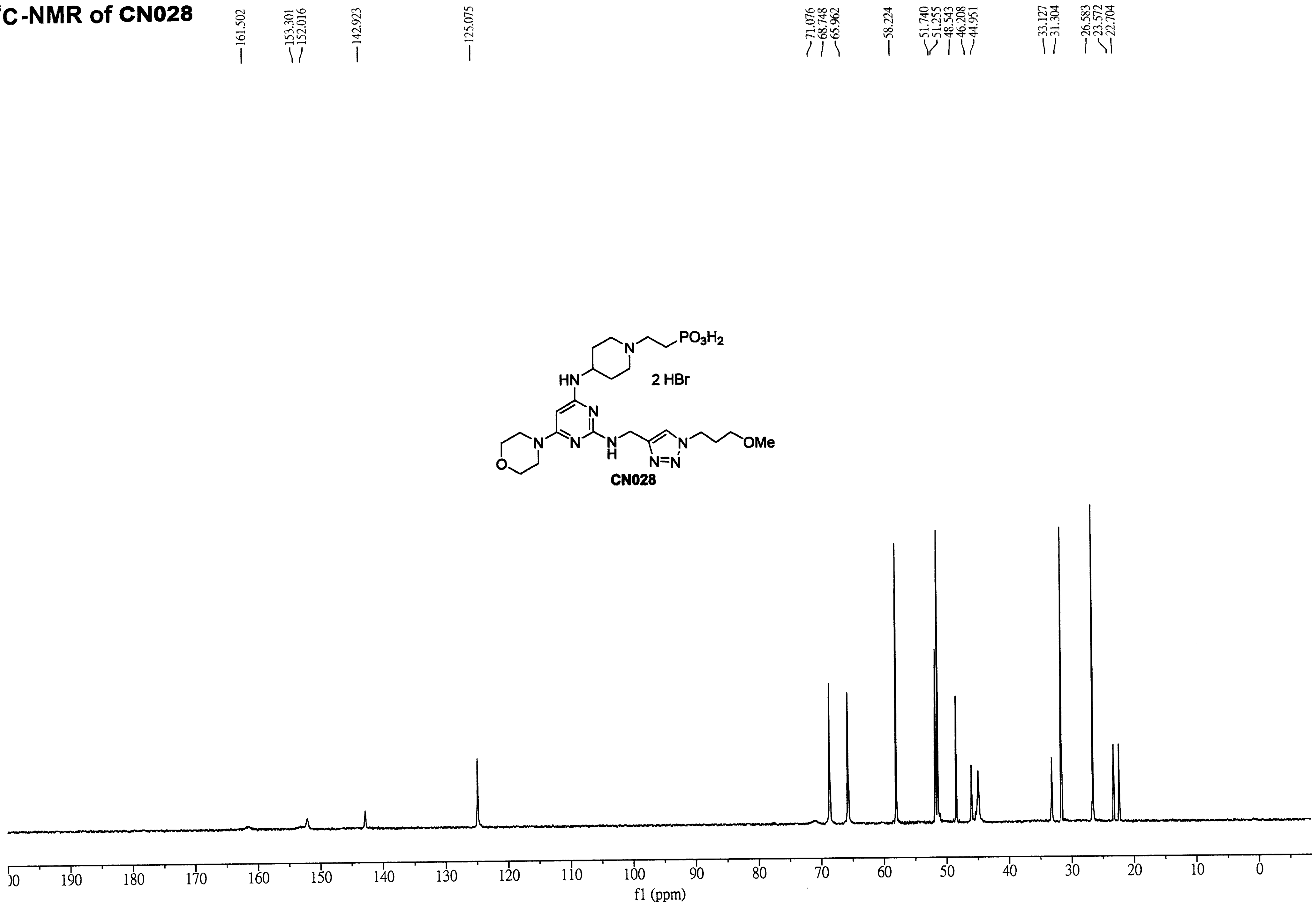

Purity test of CN004 by HPLC

Instrument 1 (offline 2): Data Analysis

File Graphics Integration Calibration Report Batch View Abort Help

Data Analysis | HCN4B.D | ABMEOH25.M | Integration / Report | Short

VWD1 A, Wavelength=254 nm, T

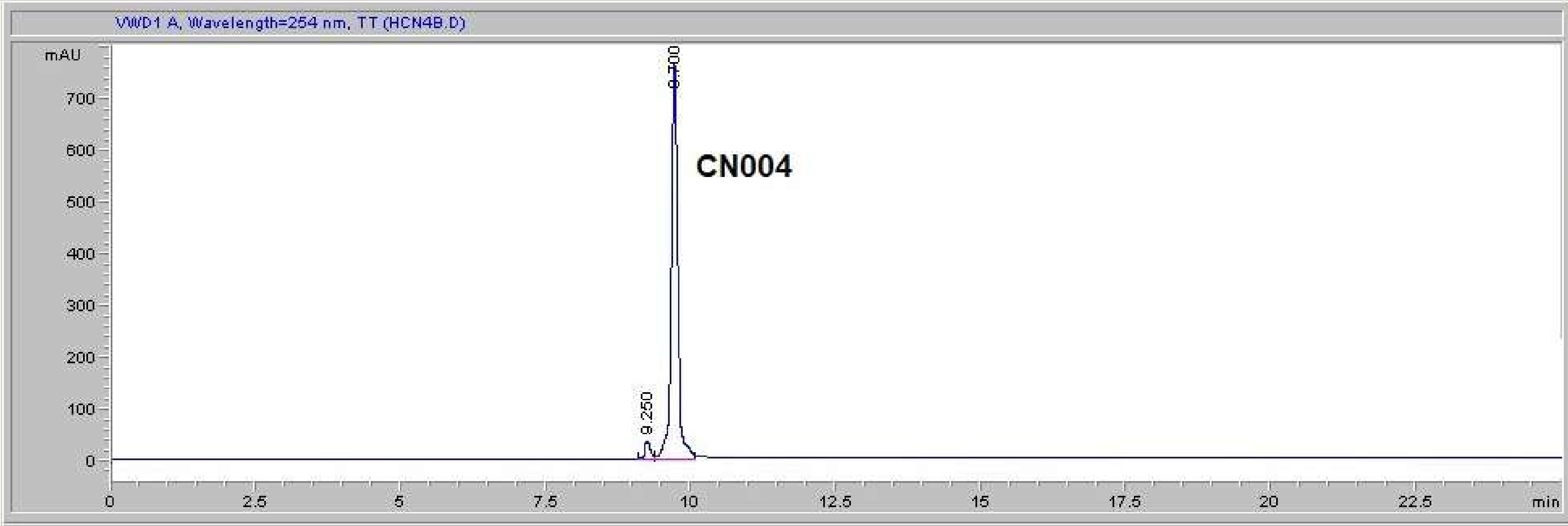

File Information

LC-File	HCN4B.D
File Path	D:\HPLC\DATA\
Date	07-Oct-21, 10:53:51
Sample	CN004
Sample Info	
Barcode	
Operator	
Method	ABMEOH25.M
Analysis Time	24.997 min
Sampling Rate	0.0012 min (0.072 sec), 20603 datapoints

#	Time	Area	Height	Width	Area%	Symmetry
1	9.25	298.3	37.2	0.1227	4.758	0.911
2	9.7	5970.5	761.9	0.1176	95.242	0.828

Purity test of CN012 by HPLC

Instrument 1 (offline 2): Data Analysis

File Graphics Integration Calibration Report Batch View Abort Help

Data Analysis | HCN12A.D | ABMEOH25.M | Integration / Report | Short

VWD1 A, Wavelength=254 nm, T

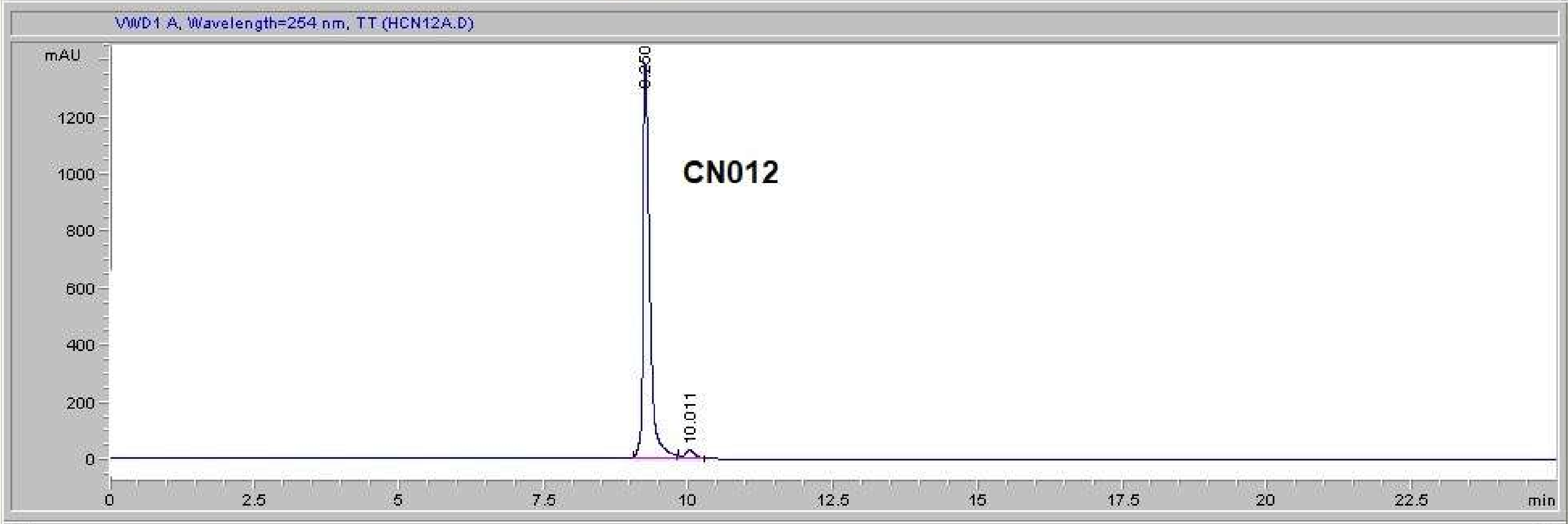

File Information

LC-File	HCN12A.D
File Path	D:\HPLC\DATA\
Date	04-Oct-21, 23:11:56
Sample	HCN12
Sample Info	
Barcode	
Operator	
Method	ABMEOH25.M
Analysis Time	24.996 min
Sampling Rate	0.0012 min (0.072 sec), 20602 datapoints

#	Time	Area	Height	Width	Area%	Symmetry
1	9.25	12078.9	1379.5	0.1342	96.583	0.68
2	10.011	427.3	31.9	0.1942	3.417	0.949

Select Integration Task

Purity test of CN016 by HPLC

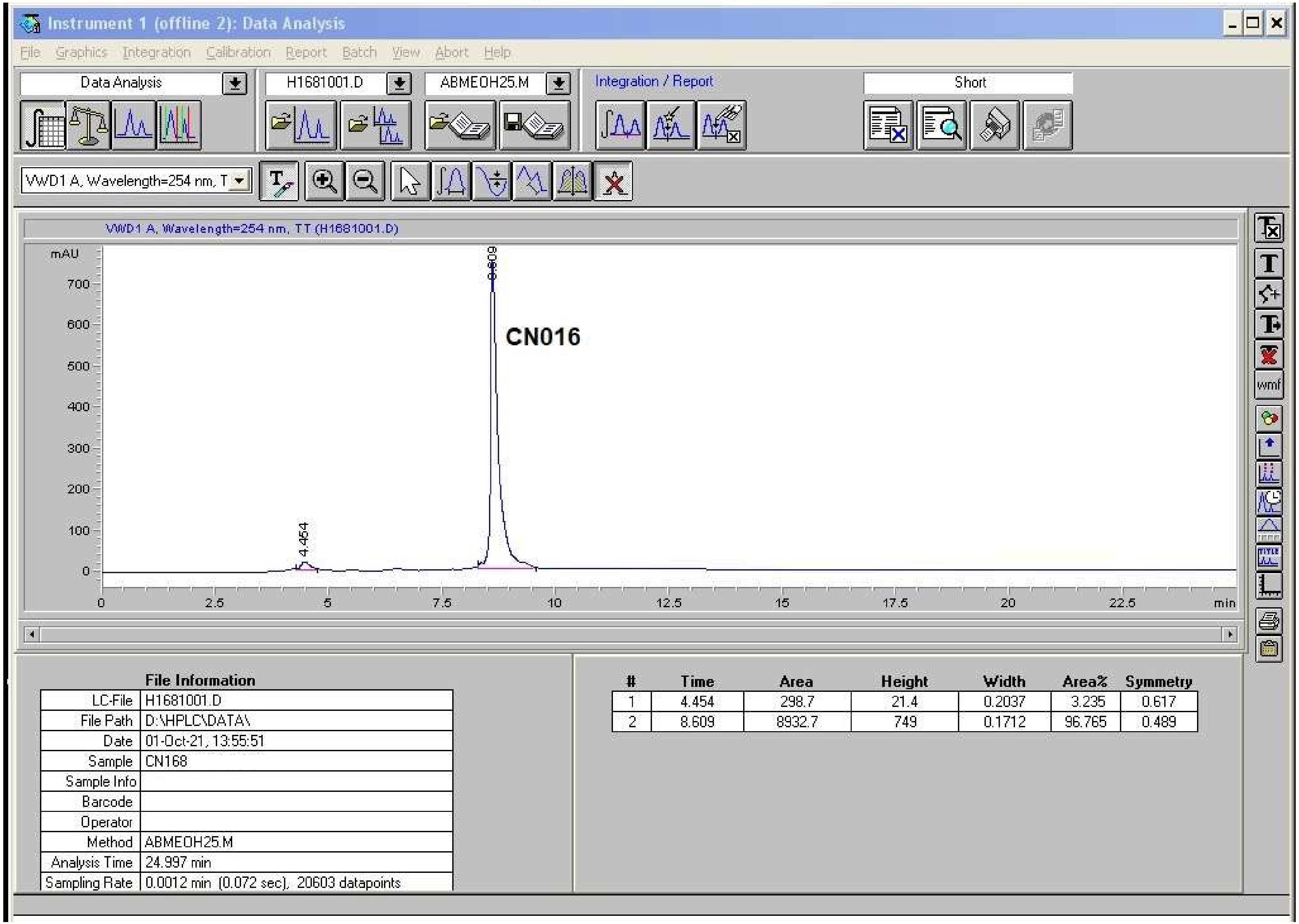

File Information

LC-File	H1681001.D
File Path	D:\HPLC\DATA\
Date	01-Oct-21, 13:55:51
Sample	CN168
Sample Info	
Barcode	
Operator	
Method	ABMEOH25.M
Analysis Time	24.997 min
Sampling Rate	0.0012 min (0.072 sec), 20603 datapoints

#	Time	Area	Height	Width	Area%	Symmetry
1	4.454	298.7	21.4	0.2037	3.235	0.617
2	8.609	8932.7	749	0.1712	96.765	0.489

Purity test of CN020 by HPLC

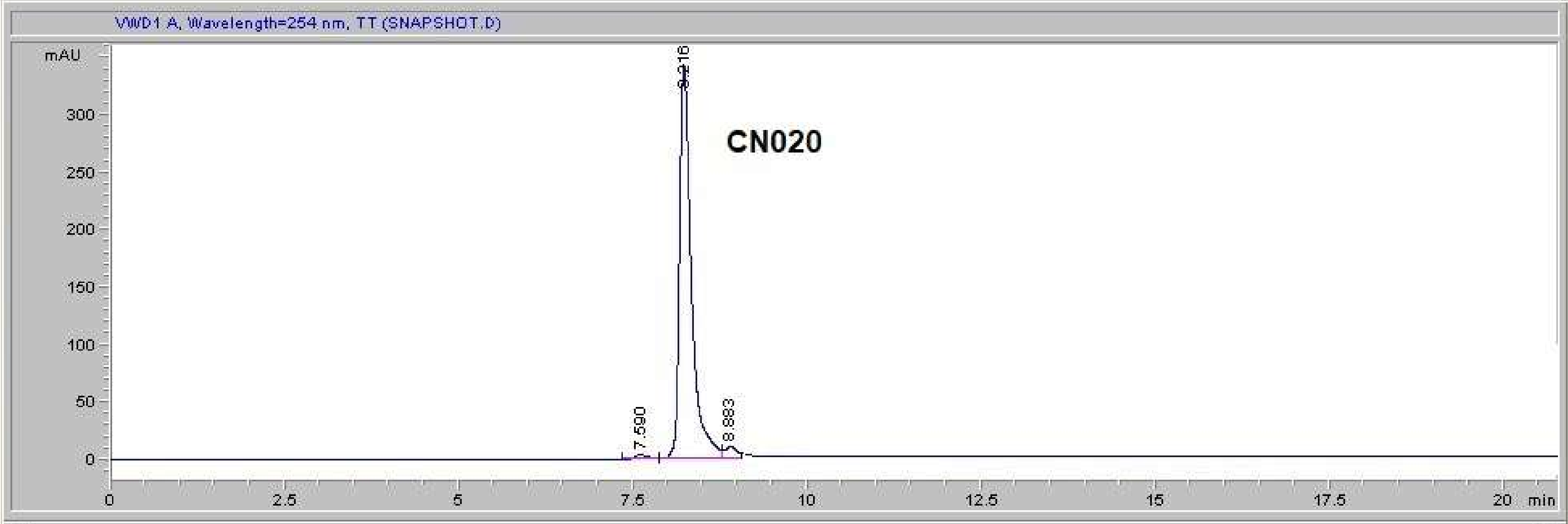

File Information

LC-File	SNAPSHOT.D
File Path	D:\HPLC\DATA\
Date	04-Nov-21, 10:51:13
Sample	CN20
Sample Info	
Barcode	
Operator	
Method	ABMEOH25.M
Analysis Time	20.756 min
Sampling Rate	0.0012 min (0.072 sec), 17108 datapoints

#	Time	Area	Height	Width	Area%	Symmetry
1	7.59	55.8	3.8	0.2151	1.309	0.657
2	8.216	4076.7	342.8	0.1766	95.553	0.54
3	8.883	133.9	10.6	0.1823	3.138	0.733

Purity test of CN021 by HPLC

Instrument 1 (offline 2): Data Analysis

File Graphics Integration Calibration Report Batch View Abort Help

Data Analysis | HCN2105.D | ABMEOH25.M | Integration / Report | Short

VWD1 A, Wavelength=254 nm, T

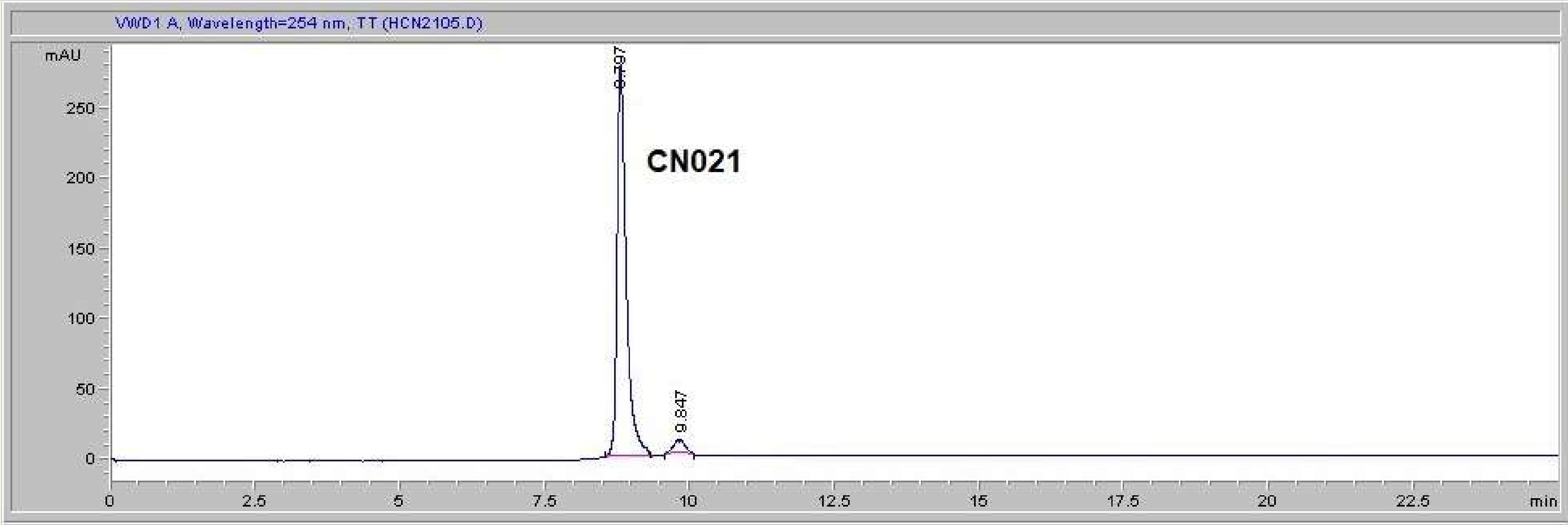

File Information

LC-File	HCN2105.D
File Path	D:\HPLC\DATA\
Date	05-Nov-21, 11:03:54
Sample	cn21
Sample Info	
Barcode	
Operator	
Method	ABMEOH25.M
Analysis Time	24.997 min
Sampling Rate	0.0012 min (0.072 sec), 20603 datapoints

#	Time	Area	Height	Width	Area%	Symmetry
1	8.797	2976.9	278.4	0.1782	95.402	0.543
2	9.847	143.5	10.6	0.226	4.598	1.141

Zoom out active Window

Purity test of CN022 by HPLC

Instrument 1 (offline 2): Data Analysis

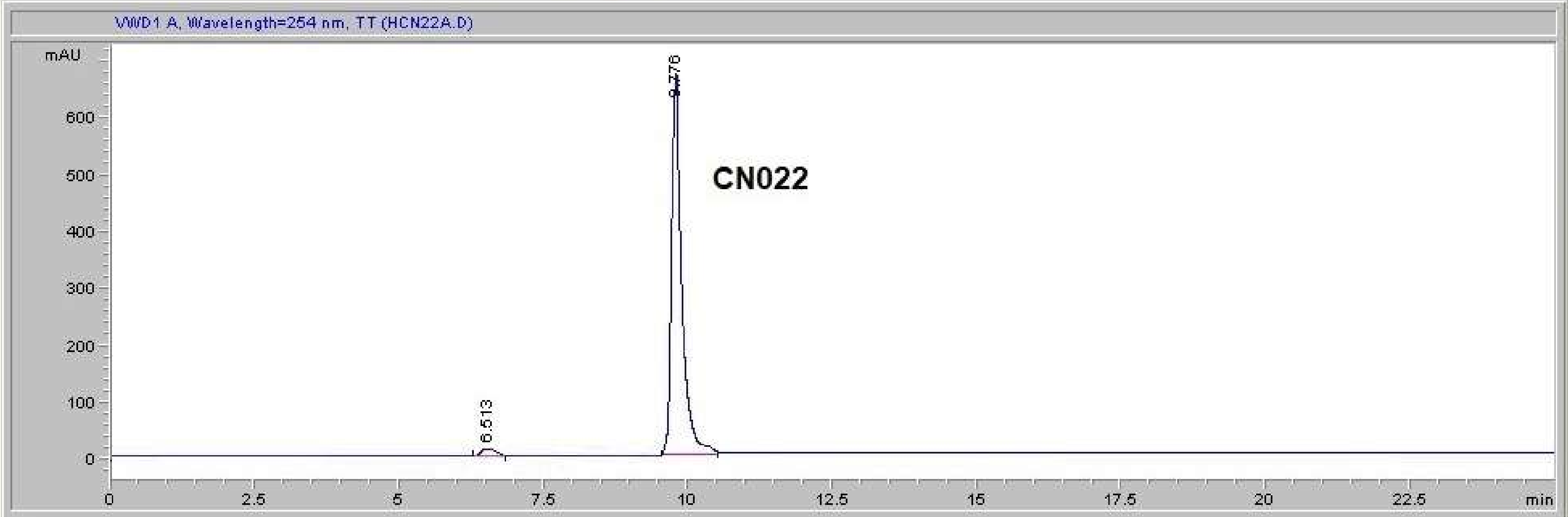

File Information

LC-File	HCN22A.D
File Path	D:\HPLC\DATA\
Date	05-Oct-21, 00:15:55
Sample	HCN22
Sample Info	
Barcode	
Operator	
Method	ABMEOH25.M
Analysis Time	24.997 min
Sampling Rate	0.0012 min (0.072 sec), 20603 datapoints

#	Time	Area	Height	Width	Area%	Symmetry
1	6.513	425.7	24.5	0.4397	4.453	0.596
2	9.776	9123.1	677	0.4038	95.547	0.62

Purity test of CN023 by HPLC

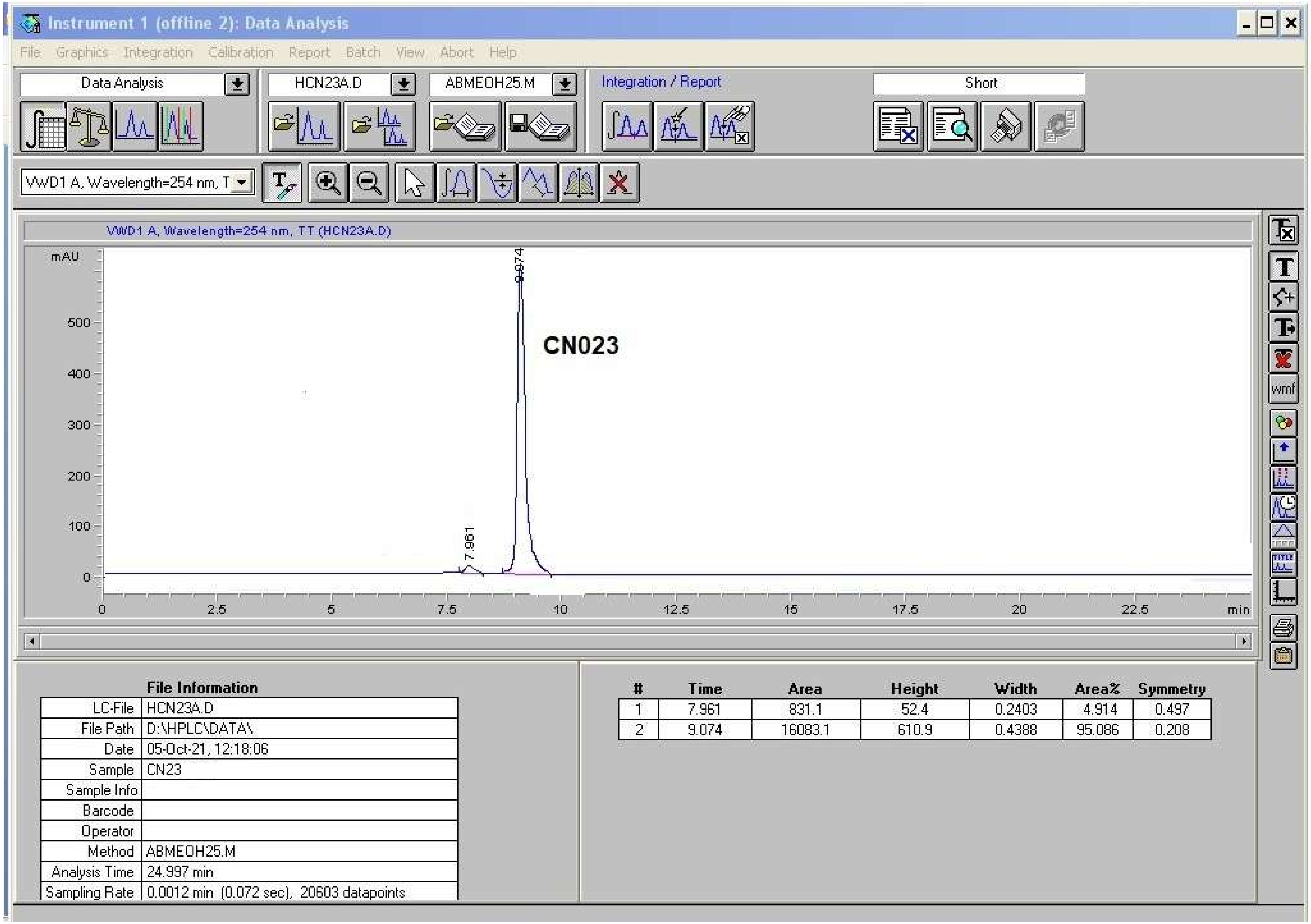

File Information

LC-File	HCN23A.D
File Path	D:\HPLC\DATA\
Date	05-Oct-21, 12:18:06
Sample	CN23
Sample Info	
Barcode	
Operator	
Method	ABMEOH25.M
Analysis Time	24.997 min
Sampling Rate	0.0012 min (0.072 sec), 20603 datapoints

#	Time	Area	Height	Width	Area%	Symmetry
1	7.961	831.1	52.4	0.2403	4.914	0.497
2	9.074	16083.1	610.9	0.4388	95.086	0.208

Purity test of CN024 by HPLC

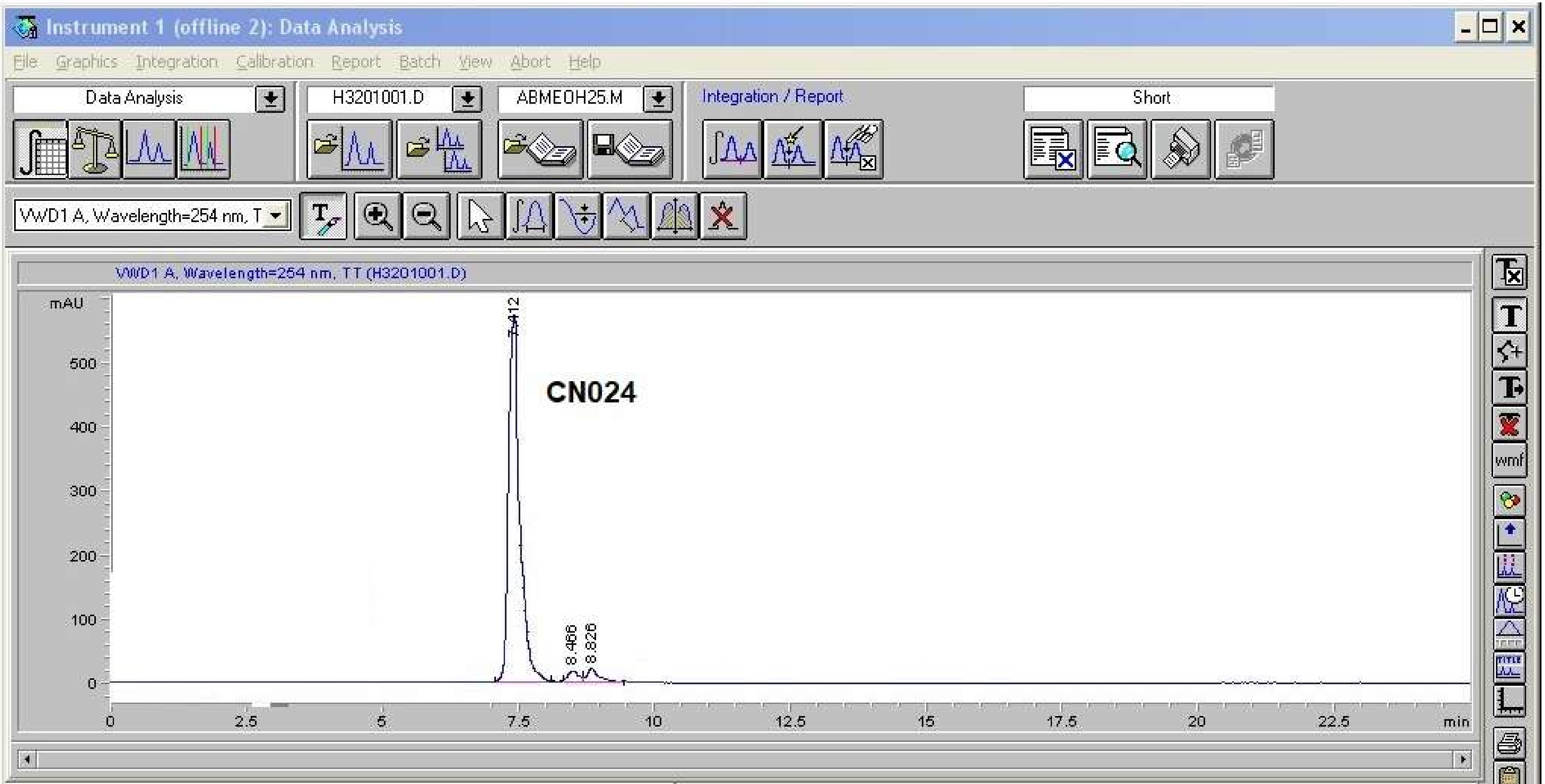

File Information	
LC-File	H3201001.D
File Path	D:\HPLC\DATA\
Date	01-Oct-21, 14:27:51
Sample	CN320
Sample Info	
Barcode	
Operator	
Method	ABMEOH25.M
Analysis Time	24.997 min
Sampling Rate	0.0012 min (0.072 sec), 20603 datapoints

#	Time	Area	Height	Width	Area%	Symmetry
1	7.412	12135.5	577.3	0.3122	95.343	0.462
2	8.466	264	18.7	0.2027	2.074	0.642
3	8.826	328.7	20.9	0.2152	2.582	0.487

Purity test of CN025 by HPLC

Instrument 1 (offline 2): Data Analysis

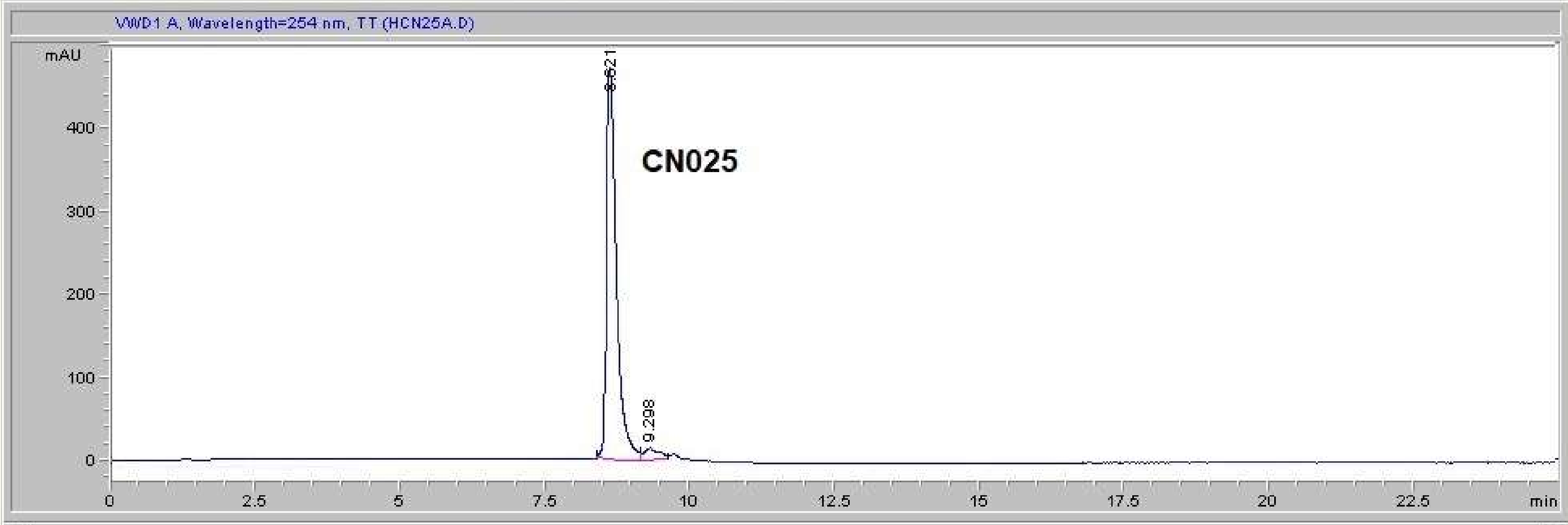

File Information

LC-File	HCN25A.D
File Path	D:\HPLC\DATA\
Date	05-Oct-21, 00:47:55
Sample	HCN25
Sample Info	
Barcode	
Operator	
Method	ABMEOH25.M
Analysis Time	24.997 min
Sampling Rate	0.0012 min (0.072 sec), 20603 datapoints

#	Time	Area	Height	Width	Area%	Symmetry
1	8.621	6088.7	472.2	0.1941	95.548	0.641
2	9.298	283.7	15.1	0.2548	4.452	0.452

Select Integration Task

Purity test of CN026 by HPLC

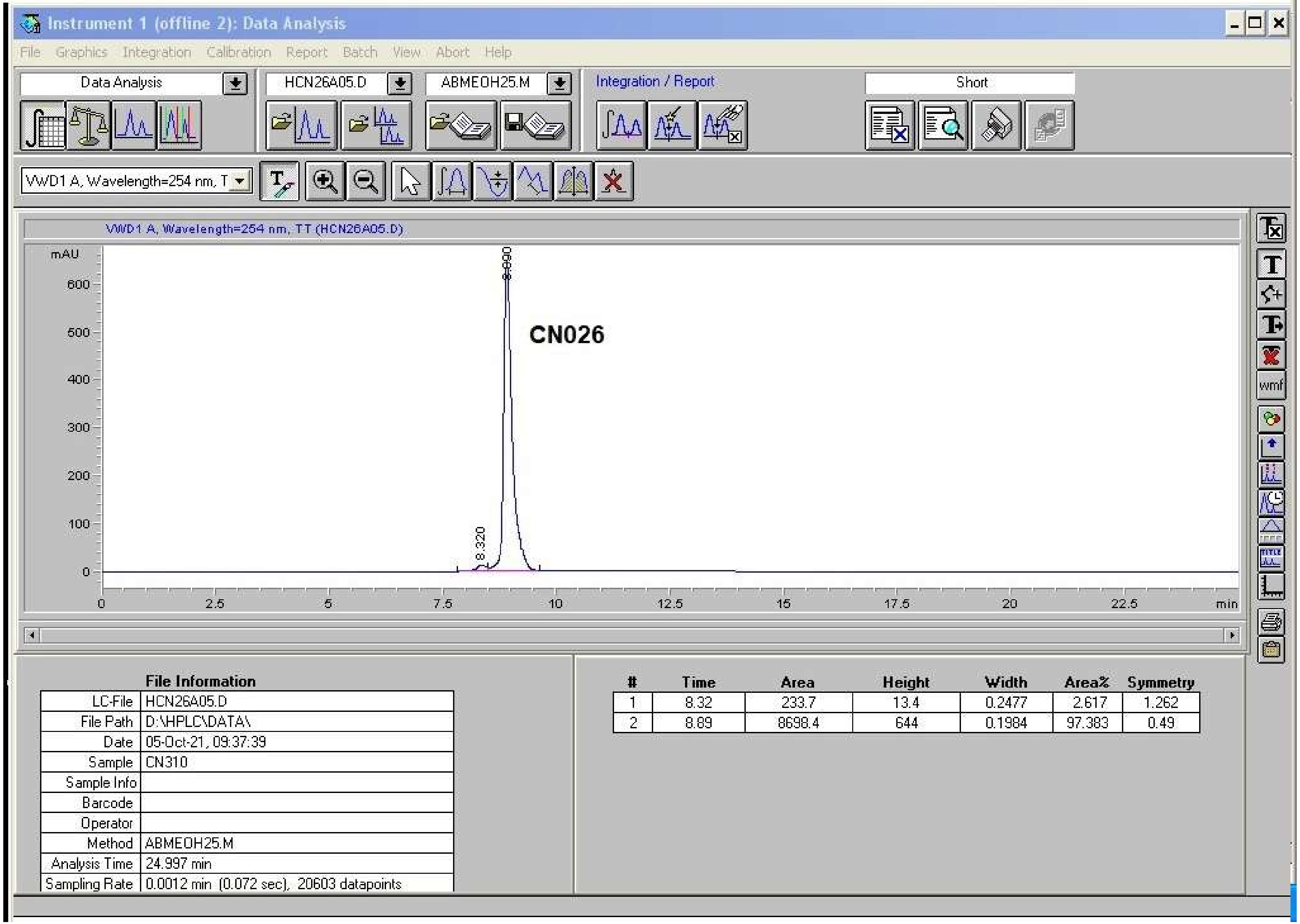

File Information

LC-File	HCN26A05.D
File Path	D:\HPLC\DATA\
Date	05-Oct-21, 09:37:39
Sample	CN310
Sample Info	
Barcode	
Operator	
Method	ABMEOH25.M
Analysis Time	24.997 min
Sampling Rate	0.0012 min (0.072 sec), 20603 datapoints

#	Time	Area	Height	Width	Area%	Symmetry
1	8.32	233.7	13.4	0.2477	2.617	1.262
2	8.89	8698.4	644	0.1984	97.383	0.49

Purity test of CN027 by HPLC

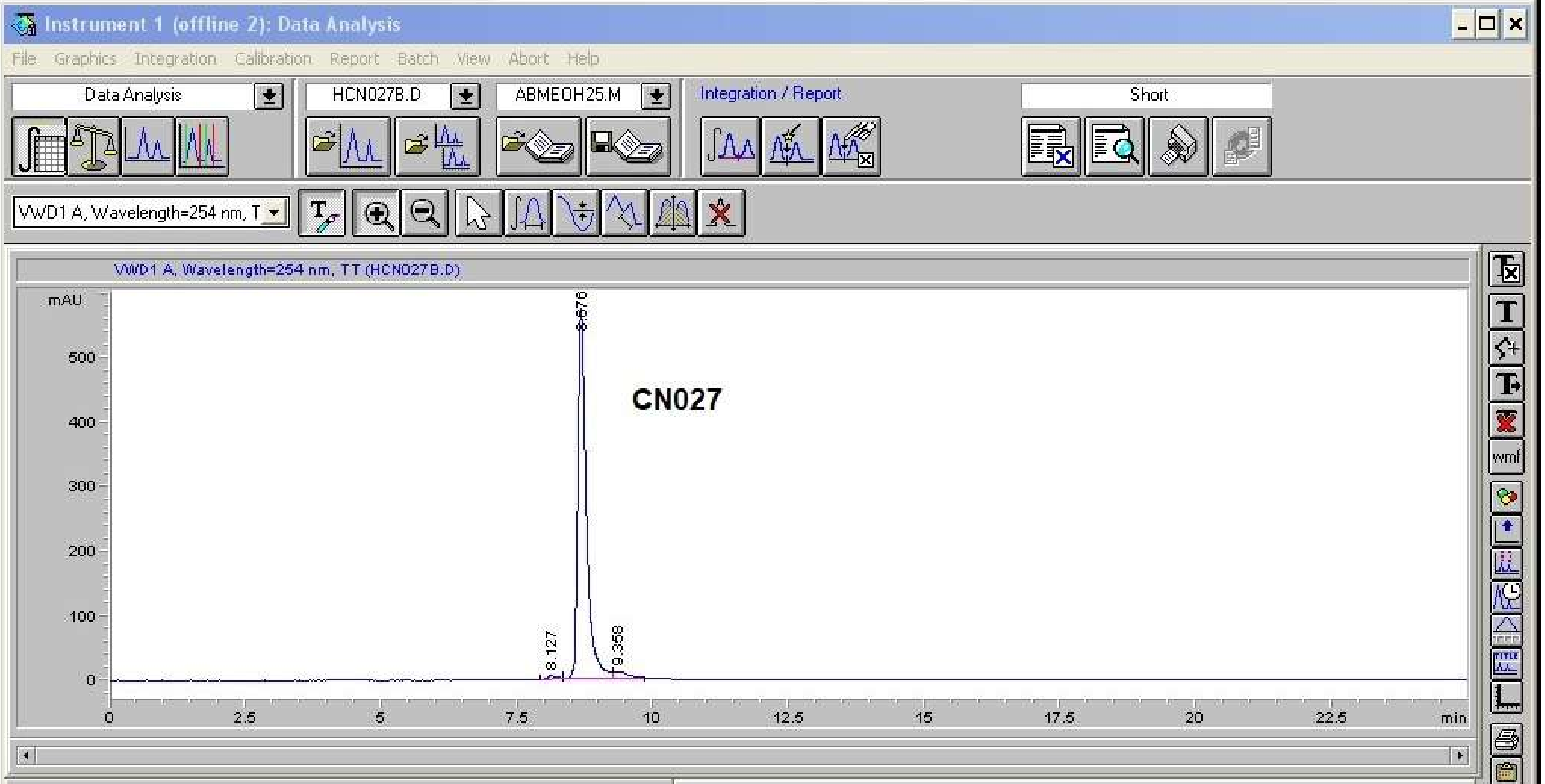

File Information

LC-File	HCN027B.D
File Path	D:\HPLC\DATA\
Date	05-Oct-21, 19:04:30
Sample	CN027(308)
Sample Info	
Barcode	
Operator	
Method	ABMEOH25.M
Analysis Time	24.997 min
Sampling Rate	0.0012 min (0.072 sec), 20603 datapoints

#	Time	Area	Height	Width	Area%	Symmetry
1	8.127	85.7	6.3	0.1992	1.367	0.584
2	8.676	6057.5	573.1	0.1762	96.575	0.662
3	9.358	129.1	9.6	0.2244	2.058	0.705

Purity test of CN028 by HPLC

Instrument 1 (offline 2): Data Analysis

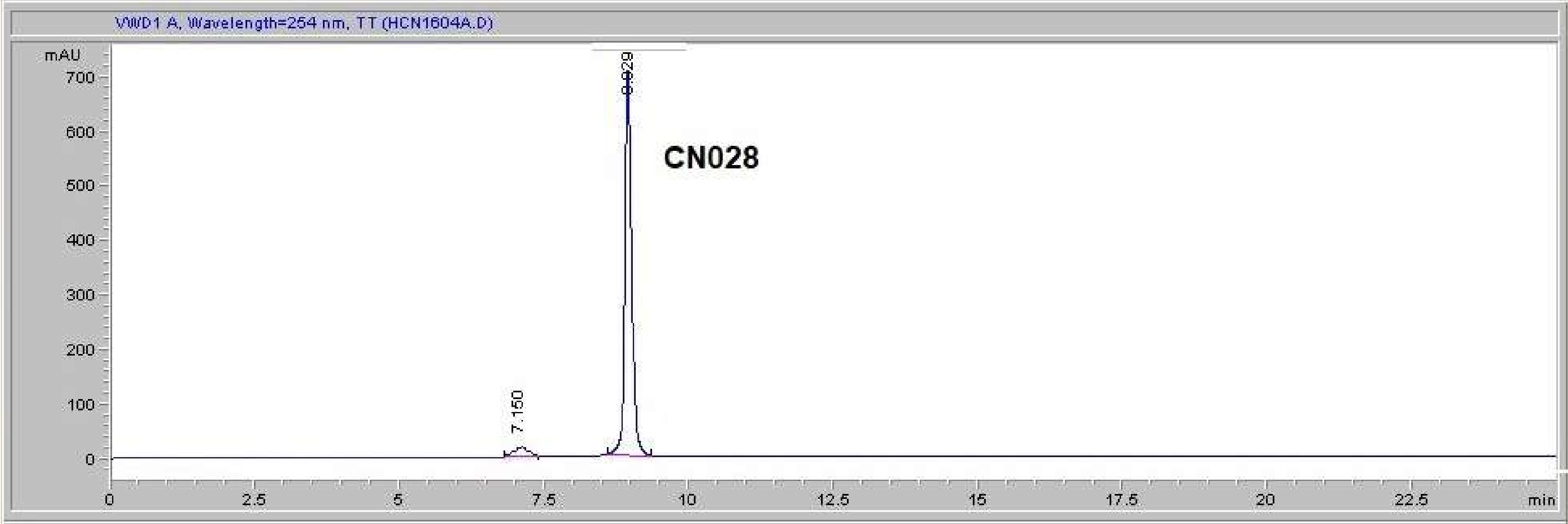

File Information

LC-File	HCN1604A.D
File Path	D:\HPLC\DATA\
Date	04-Nov-21, 09:28:15
Sample	CN28
Sample Info	
Barcode	
Operator	
Method	ABMEOH25.M
Analysis Time	24.997 min
Sampling Rate	0.0012 min (0.072 sec), 20603 datapoints

#	Time	Area	Height	Width	Area%	Symmetry
1	7.15	354.7	12.4	0.4757	4.562	0.982
2	8.929	7420	704.8	0.1755	95.438	0.396

Select Integration Task
